# Assembly and analysis of the first complete mitochondrial genome of *Punica granatum* and the gene transfer from chloroplast genome

**DOI:** 10.3389/fpls.2023.1132551

**Published:** 2023-06-21

**Authors:** Guilong Lu, Kai Zhang, Youxiong Que, Yanfeng Li

**Affiliations:** ^1^Institute of Vegetables, Tibet Academy of Agricultural and Animal Husbandry Sciences, Lhasa, China; ^2^Key Laboratory of Sugarcane Biology and Genetic Breeding, Ministry of Agriculture and Rural Affairs, Fujian Agriculture and Forestry University, Fuzhou, China

**Keywords:** *Punica granatum*, mitochondrial genome, repeat sequences, systematic evolution, RNA editing events

## Abstract

Pomegranate (*Punica granatum* L.) is one of the oldest fruits with edible, medicinal and ornamental values. However, there is no report on the mitochondrial genome of pomegranate. In this study, the mitochondrial genome of *P. granatum* was sequenced, assembled and analyzed in detail, while the chloroplast genome was assembled using the same set of data. The results showed that the *P. granatum* mitogenome had a multi branched structure, using BGI + Nanopore mixed assembly strategy. The total genome length was 404,807 bp, with the GC content of 46.09%, and there were 37 protein coding genes, 20 tRNA genes and three rRNA genes. In the whole genome, 146 SSRs were identified. Besides, 400 pairs of dispersed repeats were detected, including 179 palindromic, 220 forward and one reverse. In the *P. granatum* mitochondrial genome, 14 homologous fragments of chloroplast genome were found, accounting for 0.54% of the total length. Phylogenetic analysis showed that among the published mitochondrial genomes of related genera, *P. granatum* had the closest genetic relationship with *Lagerstroemia indica* of Lythraceae. The 580 and 432 RNA editing sites were predicted on 37 protein coding genes of mitochondrial genome using BEDTools software and online website PREPACT respectively, but all were from C to U, of which *ccmB* and *nad4* gene were most frequently edited, with 47 sites. This study provides a theoretical basis for understanding the evolution of higher plants, species classification and identification, and will also be useful for further utilization of pomegranate germplasm resources.

## Introduction

Pomegranate (*Punica granatum* L.), one of the oldest fruits known to mankind, is a perennial deciduous shrub or arbor fruit tree (Holland et al., 2009). It is native to Iran, Afghanistan and other Central Asian regions (Verma et al., 2010). At present, pomegranate is widely planted in subtropical and tropical regions, with a total planting area of more than 600 kha and an annual total output of about 6.0 million tons. In China, the planting area and output are about 120 kha and 1.7 million tons, respectively, both ranking first in the world (Tao et al., 2019). Pomegranate fruit is rich in punicic acid, quinic acid, flavanoids, phenolics, vitamin C and other characteristic nutrients, and has antioxidant, anti-cancer, anti-inflammatory and other effects (Sreekumar et al., 2014; Akhtar et al., 2015; Passafiume et al., 2019; Paul and Radhakrishnan, 2020). Therefore, it is also known as “super fruit”. Besides, pomegranate flowers, leaves, bark and other parts contain a variety of active substances beneficial to human health, which has the potential for medicinal value development (Amri et al., 2017). In addition, pomegranate is of great ornamental value mostly due to its beautiful tree shape, long flowering period, beautiful fruit shape and long fruit bearing period (Da-Silva et al., 2013). In recent years, the pomegranate industry has developed rapidly, which means that high-quality, high-yield, biotic and abiotic stress resistant varieties are urgently needed in production. The key to achieve this modern breeding goal is how to reasonably develop pomegranate germplasm resources (Gunnaiah et al., 2021). With the development of genetics and bioinformatics, great progress has been made in genome, transcriptome, small RNA analysis and other aspects in pomegranate, laying a scientific foundation for further improving pomegranate traits and ensuring industrial safety (Ophir et al., 2014; Qin et al., 2017; Yuan et al., 2018; Chen et al., 2020).

Mitochondria, the main places for eukaryotes to carry out oxidative metabolism and energy transformation, are a kind of organelles that widely exist in eukaryotic cells. They are also known as the “energy factory” or “power house” of cells (Klingenberg, 2008). Mitochondria are semi-autonomous organelles, and their genetic system is relatively independent of the nucleus (Waltz and Giegé, 2020). Plant mitochondria genome, which has become an important tool for studying the origin, classification and phylogeny of species (Chen et al., 2017; Sibbald et al., 2021; Fan et al., 2022), are often characterized by extreme variation in genome size, very sparse gene distribution, a large number of non-coding sequences, rich repetitive sequences, ability to incorporate foreign DNA, highly conservative gene sequences, and a large number of RNA editing (Petersen et al., 2020; Li et al., 2021; Yang et al., 2022; Yu et al., 2022). Recently, a large number of studies have shown that mitochondria also play a unique and important role in plant growth and development, and are closely related to agronomic traits such as cytoplasmic male sterility (Kubo et al., 2011; Liao et al., 2020), disease resistance and plant growth vigor (Liberatore et al., 2016; Chevigny et al., 2020). However, due to the complexity of plant mitochondrial genome structure, its research lags largely behind chloroplast and plastid genomes. Up to now, 9,479 chloroplast and 1,253 plastid genomes have been published in the NCBI database, while only 560 plant mitochondrial genomes have been released (2022, December, 25th, https://www.ncbi.nlm.nih.gov/genome/browse/#!/organelles/), and there is no report on the mitochondrial genome of pomegranate.

*Punica* genera has only two species, namely, *P. protopunica* Balf. and *P. granatum* L. Among them, *P. protopunica* is only found in Socotra archipelago in the northwestern Indian Ocean, and is considered to be the ancestor of pomegranate, while *P. granatum* is the main cultivated specie in the world (Guerrero-Solano et al., 2020). In the present study, *P. granatum* was selected as the research object to sequence, assemble and analyze the mitochondrial genome. Besides, the chloroplast genome was assembled using the same set of data, and its homology with the mitochondrial genome was also analyzed. At the same time, phylogenetic analysis was conducted with related species, and RNA editing events in their mitochondrial genomes were predicted. The present study aims to set up the theoretical basis for understanding the evolution, classification and identification of pomegranate, which should also be useful for further utilization of pomegranate germplasm resources.

## Materials and methods

### Plant material and sample preparation

The tender leaves of pomegranate plant (Qingpi Sweet) were collected from Laixi Fine Variety Breeding Farm, Qingdao (120˚29’34’’ E, 36˚51’39’’ N). After the leaves were washed and dried with DEPC-treated water, they were immediately cooled in liquid nitrogen and stored in an ultra-low temperature refrigerator at -80°C. The plant genomic DNA Kit (TianGen Biotech, Beijing, China) was used to extract the total DNA, and the CTAB method was used to extract the total RNA. NanoDrop One Microvolume UV Vis Spectrophotometer (Thermo Fisher Scientific, Massachusetts, USA) was used to detect the DNA/RNA quality. The DNA of OD_260_/OD_280 = _1.7~1.9, OD_260_/OD_230 = _2.3~2.5, and RNA of OD_260_/OD_280 = _1.8~2.1, OD_260_/OD_230 = _2.3~2.5 were assumed to be high-quality. They were placed in dry ice medium, and send to Wuhan Benagen Tech Solutions Company Limited (Wuhan, China) for sequencing.

### Sequencing and database building

The short reads sequencing was completed by DNBSEQ-T7 (Shenzhen Huada Intelligent Technology Co., Ltd., Shenzhen, China), and the fastp software was used to perform data quality control filtering on the original reads (Chen et al., 2018a). The long reads sequencing was completed by Nanopore PromethION sequencer (Oxford Nanopore Technologies, Oxford, UK), which used NanoPack to evaluate and control the quality of original reads (De Coster et al., 2018). The long non-coding RNA was sequenced by MGISEQ-2000, and the original reads were filtered by software SOAPnuke v2.0 for quality control (Chen et al., 2018b).

### Mitochondrial genome assembly and structure prediction

The mitochondrial genome of *P. granatum* was assembled using a hybrid assembly strategy. The GetOrganelle v1.7.1 (Jin et al., 2020) was utilized to obtain the short reads belonging to the mitochondrial genome. Then the short reads were assembled into a unitig graph using GetOrganelle and was visualized with Bandage v0.8.1 (Wick et al., 2015). To ensure the integrity of mitochondrial genome, chloroplast-derived uniting nodes were manually removed if they met two criteria simultaneously: 1) much higher coverage than the mitochondrial genome contig, and 2) both contigs connected at either end of this unusually high coverage contig were also unusually high coverage contigs. Nuclear-derived uniting nodes were manually removed if their nodes were much lower than those of mitochondrial contigs. The mitochondrial genome was treated with Unicycler v0.4.7 (The University of Melbourne, Victoria, Australia) (Wick et al., 2017) to resolve repetitive regions.

### Genome annotation and codon preference analysis

The protein-coding genes (PCGs) of the *P. granatum* mitochondrial genome were annotated using Geseq (https://chlorobox.mpimp-golm.mpg.de/geseq.html) (Tillich et al., 2017) with reference genomes under the same Order, including *Eucalyptus grandis* (NC_040010.1), *Oenothera biennis* (MZ934756.1), *Lagerstroemia indica* (NC_035616.1), *Medinilla magnifica* (MT043351.1), *Oenothera elata* (MZ934757.1), and *Oenothera villaricae* (MZ934755.1). The tRNA of mitochondrial genome was annotated with tRNAscan-SE v2.0 (Lowe and Eddy, 1997). The rDNA of mitochondrial genome was annotated using BLASTN software (Chen et al., 2015). Apollo v1.11.8 was used to manually correct those annotation errors of mitochondrial genome (Lewis et al., 2002).

PhyloSuite v1.1.12 (Zhang et al., 2020) was used to extract the PCG sequence of *P. granatum* mitochondrial genome. Mega v7.0.26 (Kumar et al., 2016) was applied to analyze the relative synonymous codon usage (RSCU) of PCG and calculate the RSCU value. If RSCU = 1, there is no preference for the use of this codon, and if RSCU > 1, the codon is used preferentially by amino acids, while if RSCU < 1, the codon usage is contrary.

### Repetitive sequence and homology analysis

The simple sequence repeat (SSR) is a special tandem repeat sequence, which generally does not exceed 6 bp. Our study used MISA (https://webblast.ipk-gatersleben.de/misa/) to identify SSRs with default parameters (Beier et al., 2017). The tandem repeats with repeat unit > 6 bp were detected using TRF (https://tandem.bu.edu/trf/trf.unix.help.html) with the parameters ‘2 7 7 80 10 50 500 -f -d -m’ (Benson, 1999). The dispersed repeats were detected using REPuter web server (https://bibiserv.cebitec.uni-bielefeld.de/reputer/) with the minimal repeat size set to 30 bp (Kurtz et al., 2001).

GetOrganelle software was used to assemble the *P. granatum* chloroplast genome (Jin et al., 2020), and then CPGAVAS2 (http://47.96.249.172:16019/analyzer/annotate) was applied to annotate the genome (Shi et al., 2019). Homologous DNA fragments were discovered between chloroplast genome and mitochondrial genome of *P. granatum* by BLASTN with the e-value of 1*e*-6 (Chen et al., 2015), and ECircos package (Zhang et al., 2013) was used to visualize the results.

### Phylogenetic evolution and collinearity analysis

The published mitochondrial genomes of 29 species that are closely related to *P. granatum* were downloaded from NCBI for phylogenetic analysis (**Supplementary Table 1**), and *Zygophylllum fabago* (MK431827.1) and *Tribulus terrestris* (MK431825.1) of Zygophyllales were set as the outgroup. PhyloSuite software was used to extract common genes of these mitochondrial genomes (Zhang et al., 2020), MAFFT v7.450 for multiple sequence alignment (Katoh and Standley, 2013), IQ-TREE v1.6 for phylogenetic analysis (Nguyen et al., 2015) with “GTR+F+I+I+R2” model was chosen based on BIC, and finally iTOL v6 (https://itol.embl.de/) was applied to visualize the maximum likelihood tree (Letunic and Bork, 2019).

The mitochondrial genome of *P. granatum* was compared with the mitochondrial genome sequence information of six related species published under Myrtales, and the BLASTN results of mitochondrial genome pairwise comparison were obtained based on BLASTN program. Homologous sequences with a length of more than 500 bp were retained as conservative collinear blocks, and the source program of MCscanX (https://help.rc.ufl.edu/doc/MCScanX) was used to plot the Multiple Syntony Plot (Wang et al., 2012).

### RNA editing prediction and validation

Based on transcriptome sequences data, the transcripts from *P. granatum* mitochondrial genome were obtained by filtering, and mapped to mitochondrial DNA sequences by using TopHat2 (Kim et al., 2013). Possible RNA editing sites were identified by BEDTools v2.30.0 software (Quinlan and Hall, 2010) and positions with coverage depth >250× and editing frequency >0.1 were selected. At the same time, the RNA editing events was predicted based on the online website PREPACT v3.12.0 (http://www.prepact.de/) (Lenz et al., 2018), and the setting standard is: cutoff value = 0.001.

Primer-BLAST (https://www.ncbi.nlm.nih.gov/tools/primer-blast) was used to design primers for the RNA editing sites, as detailed in **Supplementary Table S2**. cDNA was reverse transcribed from the extracted RNA using HiScript III 1st Strand cDNA Synthesis Kit (Vazyme, Nanjing, China). The PCR amplification was performed with 2 μL of template, 2 μL of upstream and downstream primers respectively, 25 μL of 2 × Taq Master Mix and 19 μL of ddH_2_O, with the program of pre-denaturation at 95°C for 3 min, denaturation at 95°C for 15 s, annealing at 55°C for 15 s, extension at 72°C for 15 s, 35 cycles, and the final extension at 72°C for 5 min. PCR products were subjected to 1% agarose gel electrophoresis, and those with correct band size were subjected to Sanger sequencing (Sino, Beijing, China).

## Results

### Mitochondrial genome assembly and structure characterization

**Figure 1:**
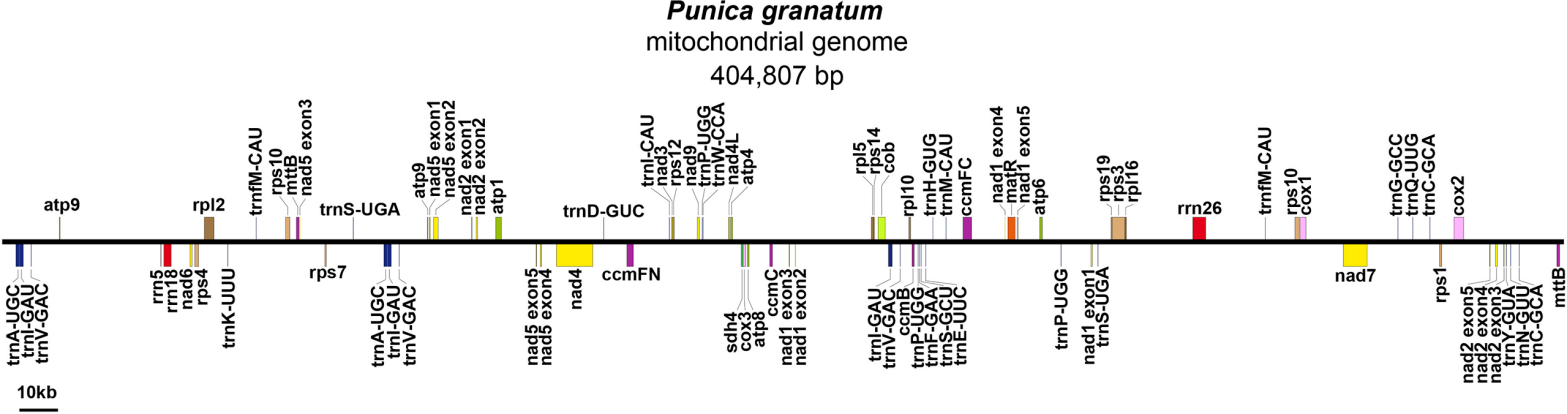
Mitochondrial genome map of *P. granatum*.

The mitochondrial genome showed a complex conformation, suggesting a multi branched structure, with a large number of repetitive sequences (**Supplementary Figure 1A**). Once those repeated areas have been excluded from the Nanopore data, a linear contig was obtained (**Supplementary Figure 1B**). In other words, a BGI + Nanopore mixed assembly strategy was adopted to simplify the genome structure into a single linear molecule (**Figure 1**), and a total length of 404,807 bp was finally obtained, with the GC content of 46.09%.

### Annotation of *P. granatum* mitochondrial genome

The mitochondrial genome of *P. granatum* was annotated, and 37 unique PCGs (**Figure 1**; **Table 1**) were characterized, including 24 unique mitochondrial core genes and thirteen variable genes, 20 tRNA genes (including seven tRNAs with multiple copies), and three rRNA genes. The core genes included five ATP synthase genes (*atp1*, *atp4*, *atp6*, *atp8* and *atp9*), nine NADH dehydrogenase genes (*nad1*, *nad2*, *nad3*, *nad4*, *nad4L*, *nad5*, *nad6*, *nad7* and *nad9*), four ubiquinol cytochrome c reductase genes (*ccmB*, *ccmC*, *ccmFc* and *ccmFn*), three cytochrome c oxidase genes (*cox1*, *cox2* and *cox3*), one membrane transporter gene (*mttB*), one maturases gene (*matR*) and one panthenol cytochrome c reductase gene (*cob*). Variable genes contained four ribosomal large subunit genes (*rpl2*, *rpl5*, *rpl10*, *rpl16*), eight ribosomal small subunit genes (*rps1*, *rps3*, *rps4*, *rps7*, *rps10*, *rps12*, *rps14*, *rps19*), and one succinate dehydrogenase gene (*sdh4*).

**Table 1:** Encoding genes of *P. granatum* mitochondrial genome.

Group of genes		Name of genes
Core genes	ATP synthase	*atp1*, *atp4*, *atp6*, *atp8*, *atp9* (×2)
	NADH dehydrogenase	*nad1*, *nad2*, *nad3*, *nad4*, *nad4L*, *nad5*, *nad6*, *nad7*, *nad9*
	Cytochrome c biogenesis	*cob*
	Ubiquinol cytochrome c reductase	*ccmB*, *ccmC*, *ccmFC*, *ccmFN*
	Cytochrome c oxidase	*cox1*, *cox2*, *cox3*
	Maturases	*matR*
	Transport membrane protein	*mttB* (×2)
Variable genes	Large subunit of ribosome	*rpl2*, *rpl5*, *rpl10*, *rpl16*
	Small subunit of ribosome	*rps1*, *rps3*, *rps4*, *rps7*, *rps10* (×2), *rps12*, *rps14*, *rps19*
	Succinate dehydrogenase	*sdh4*
rRNA genes	Ribosome RNA	*rrn5*, *rrn18*, *rrn26*
tRNA genes	Transfer RNA	*trnA-UGC* (×2), *trnC-GCA* (×2), *trnD-GUC*, *trnE-UUC*, *trnF-GAA*, *trnfM-CAU* (×2)*, trnG-GCC*, *trnH-GUG*, *trnI-CAU*, *trnI-GAU* (×3), *trnK-UUU*, *trnM-CAU*, *trnN-GUU*, *trnP-UGG* (×3), *trnQ-UUG, trnS-GCU*, *trnS-UGA* (×2), *trnV-GAC* (×3), *trnW-CCA*, *trnY-GUA*

The number in brackets represents the copy number of the gene.

### Codon preference

**Figure 2:**
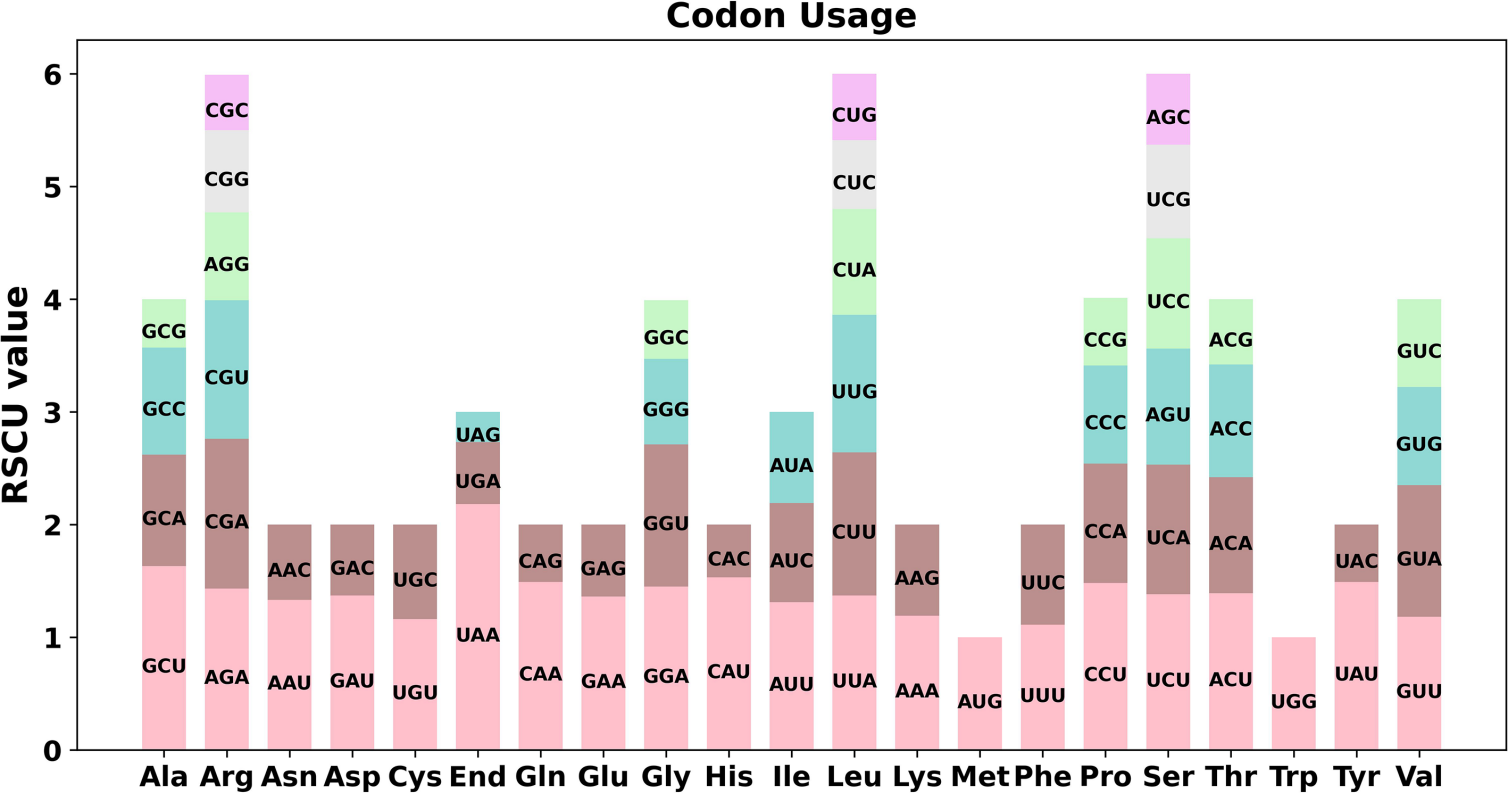
Codon preference of *P. granatum* mitochondrial genome.

Codon preference analysis was conducted on 37 unique PCGs of *P. granatum* mitochondria genome. As showed in **Figure 2** and **Supplementary Table 3**, except that the RSCU values of the initial codon (AUG) and tryptophan (UGG) were both 1, the PCGs of *P. granatum* mitochondria genome also had a common codon use preference. For example, the termination codon had a high preference for the use of UAA, and its RSCU value was the highest among mitochondrial genome PCGs, reaching 2.18, followed by alanine (Ala)’s preference for the use of GCU, and its RSCU value was 1.63. It is worth noting that the maximum RSCU values of cysteine (Cys), lysine (Lys), phenylalanine (Phe) and valine (Val) were all less than 1.2, and there was no strong codon preference.

### Repetitive sequences and their migration

**Figure 3:**
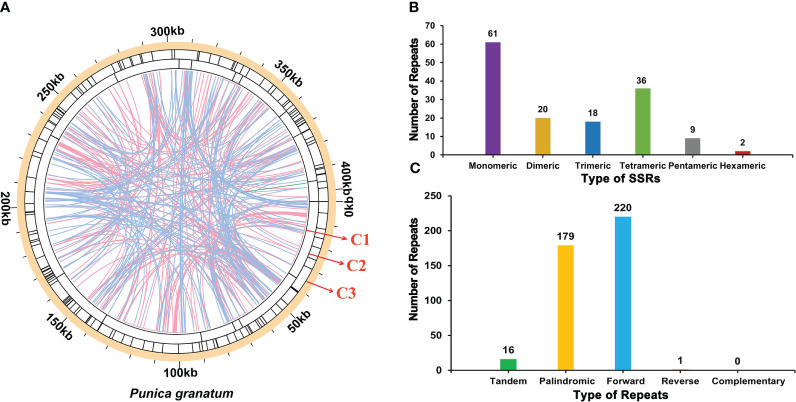
The repeat of *P. granatum* mitochondrial genome. **(A)** The repeat sequence identified in the mitogenome. The color line on the C1 circle connects two repeated dispersed repeat. The blue, pink, and green lines represent palindromic repeat, forward repeats, and reverse repeats, respectively. The C2 circle shows the tandem repeat as short bars. The C3 circle shows the SSRs. **(B)** Type and number of SSRs. The purple, yellow, blue, green, gray and red legend indicates monomer, dimer, trimer, tetramer, pentamer and hexamer SSRs, respectively. **(C)** The type and number of repeats. The green, yellow, blue and red legend indicates tandem repeats, palindromic repeats, forward repeats and reverse repeats, respectively.

There were a large number of repetitive sequences in the mitochondrial genome of *P. granatum*, and the specific distribution was showed in **Figure 3A**. A total of 146 SSRs (**Supplementary Table 4**; **Figure 3B**) were found in the whole genome, of which the SSRs in the form of monomers and dimers accounted for 55.48% of the total SSRs. It should be emphasized that adenine (A) monomer repeats accounted for 44.26% (No.27) of 61 monomer SSRs, and AT, GA and TA repeats were the most common types, accounting for 60.00% of dimer SSRs. However, only two hexamer SSRs were observed in the mitochondrial genome. At the same time, in the *P. granatum* mitochondrial genome, 16 tandem repeat sequences (**Supplementary Table 5**) were detected with a matching degree of more than 81% and a length of 12~29 bp. In addition, there were 400 pairs of dispersed repeats with a length greater than or equal to 30 bp in the mitochondrial genome, including 179 pairs of palindromic repeats, 220 pairs of forward repeats, and one pair of reverse repeats (**Supplementary Table 6**; **Figure 3C**). Among them, the longest palindromic repeat is 2,073 bp, and the longest forward repeat is 31,296 bp. Interestingly, no complementary repeat was detected.

**Figure 4:**
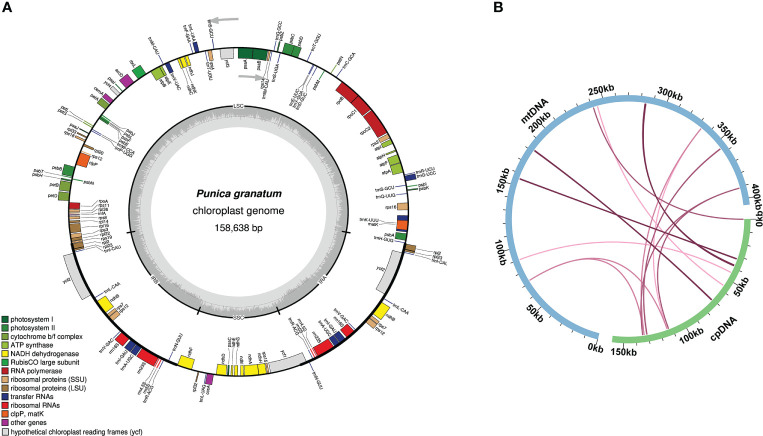
Genome map **(A)** and sequence migration **(B)** of *P. granatum* chloroplast The blue arc in the figure represents the mitochondrial genome, the green arc represents the chloroplast.

The size of the *P. granatum* chloroplast genome assembled here was 158,638 bp (**Figure 4A**). According to sequence similarity, 14 DNA fragments homologous to the chloroplast genome (**Figure 4B**; **Supplementary Table 7**) were observed in the *P. granatum* mitochondrial genome, with a total length of 2,200 bp, accounting for 0.54% of the length of the whole mitochondrial genome. Among them, there were seven complete genes, all of which were tRNA genes (*trnD-GUC*, *trnH-GUG*, *trnM-CAU*, *trnN-GUU*, *trnP-UGG*, *trnS-UGA*, *trnW-CCA*). The longest fragment was 716 bp.

### Phylogenetic evolution and sequence collinearity

**Figure 5:**
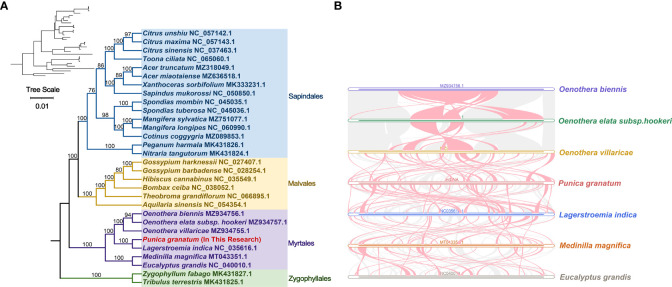
Phylogenetic evolution s **(A)** and sequence collinearity **(B)**. **(A)** displays the bootstrap support values, and the colors correspond to the families of each Order. **(B)** depicts data for seven species, with the purple, green, yellow, red, blue, orange, and gray legend representing *Oenothera biennis*, *Oenothera elata* subsp. hookeri, *Oenothera villaricae*, *Punica granatum*, *Lagerstroemia indica*, *Medinilla magnifica*, and *Eucalyptus grandis*, respectively. The red arc area represents the area where inversion occurs, and the gray area represents the area with good homology.

Based on the DNA sequences of 18 conservative mitochondrial PCGs, namely, *atp6*, *atp8*, *ccmB*, *ccmC*, *ccmFc*, *cob*, *cox1*, *cox3*, *nad2*, *nad4*, *nad5*, *nad6*, *nad7*, *nad9*, *rpl5*, *rpl16*, *rps3*, *sdh4*, the phylogenetic analysis of 30 species in four Orders including *P. granatum* was carried out (**Figure 5A**). The results showed that the *P. granatum* was closely related to *Lagerstroemia indica* of Lythraceae family of Myrtales, and the topological structure of phylogeny based on mitochondrial DNA was consistent with the latest classification of Angiosperm Phylogeny Group (APG).

In this study, we analyzed the collinearity in seven Myrtales species. Detailed results can be found in **Figure 5B** and **Supplementary Table 8**. Our findings showed that while many homologous collinear blocks were detected in *P. granatum* and other Myrtles, the length of these blocks was relatively short, indicating a high degree of non-conservation in the mitochondrial genome sequences. Furthermore, the arrangement order of the collinear blocks in Myrtles’ mitochondrial genomes was inconsistent, primarily due to the widespread genome reorganization in *P. granatum* and related species. Additionally, we identified unique blank regions in these species that lacked homology with other species.

### RNA editing events and validation

**Figure 6:**
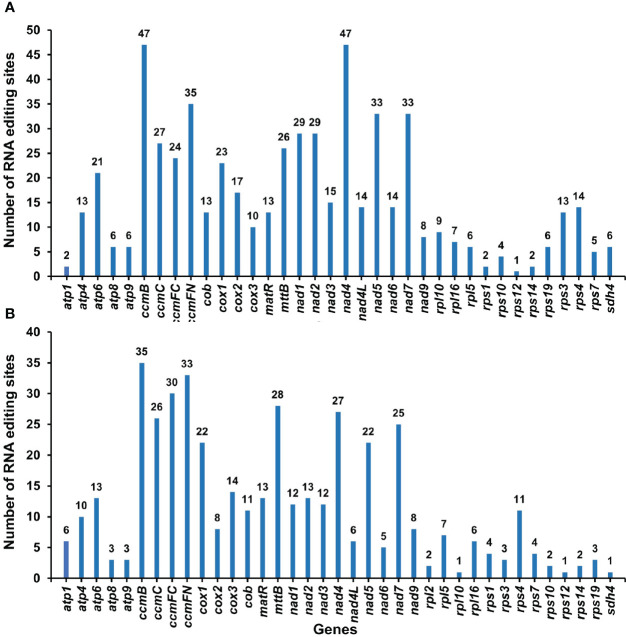
The RNA editing sites in PCGs of *P. granatum* mitochondrial genome predicted by the BEDTools software **(A)** and the online website PREPACT **(B)**.

By comparing the differences between DNA and RNA sequences of *P. granatum* mitochondrial genome with BEDTools software, 580 potential RNA editing sites were identified on 37 mitochondrial PCGs, all of which were C-U edits (**Supplementary Table 9**; **Figure 6A**). Among them, 47 sites were identified in *ccmB* and *nad4* gene, with the most editing times in all mitochondrial genes. The second was *ccmFN* gene, which had 35 RNA editing events, while *rps12* gene predicted only one RNA editing event respectively. Surprisingly, among the mitochondrial genes that predicted RNA editing events, *rps12* had the least number of edits. Furthermore, in order to confirm the correctness of RNA editing sites, 432 RNA editing sites were predicted on the same 37 mitochondrial PCGs using the online website PREPACT, which were also C to U edits (**Supplementary Table 10**; **Figure 6B**). Moreover, one editing event from ACG (threonine) to AUG (start codon) was predicted in *cox2* and *nad7* genes, respectively.

**Figure 7:**
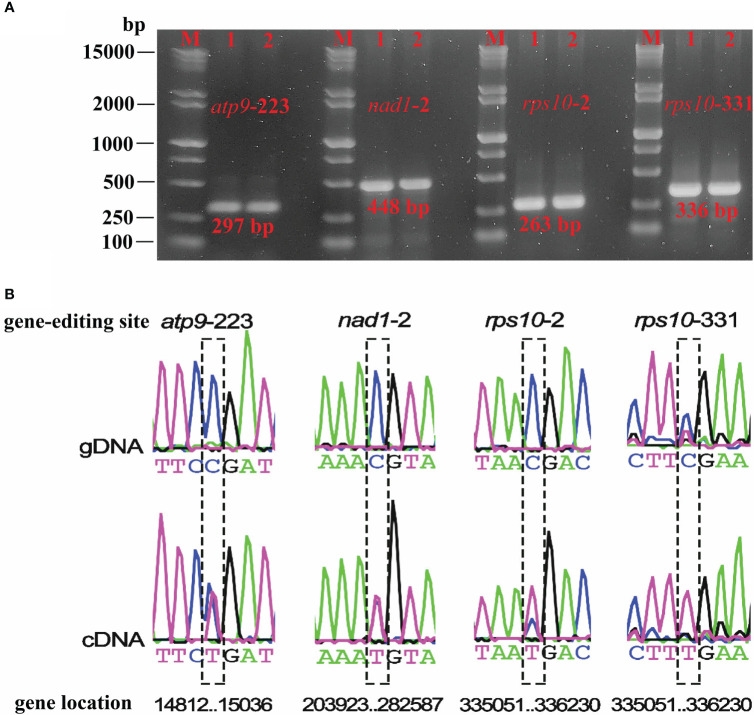
Electrophoresis plots of PCR products for the four editing sites **(A)** and sequencing editing site comparison of gDNA and cDNA **(B)**. M denotes Maker15000 + 2000, 1 denotes gDNA, 2 denotes cDNA.

Based on the RNA-seq mapping results, we verified the RNA editing sites which created the start and stop codon (*atp9*-stop-CGA, *nad1*-start-ACG, *rps10*-start-ACG, *rps10*-stop-CGA) (**Supplementary Table 9**). The RNA-seq mapping results (bam file) were provided in figshare platform (doi: 10.6084/m9.figshare.21599349). Finally, we validated four RNA editing sites that could create the start and stop codon. The PCR products and sequence comparison results were shown in **Figure 7**, and the Sanger sequencing results were provided in the **Supplementary File 1**. They were the stop codon of *atp9* (DNA: CGA, cDNA: TGA), the start codon of *nad1* (DNA: ACG, cDNA: ATG), the start (DNA: ACG, cDNA: ATG), and the stop codon (DNA: CGA, cDNA: TGA) of *rps10*. It is clear that, in plant mitochondrial genomes, RNA editing events led to changes in the start and stop codon, which could affect mitochondrial function and cellular metabolism, however further investigation in *P. granatum* was still needed.

## Discussion

A large number of studies have confirmed that the amplification of angiosperm mitochondrial genome can be attributed to the accumulation of different repetitive sequences and the incorporation of foreign sequences through horizontal or intracellular transfer (Alverson et al., 2011; Donnelly et al., 2017). Due to the highly frequent recombination, the structure and organization of angiosperm mitochondria are also diverse, such as linear, circular, highly branched, sigma-like, and networked (Chen et al., 2017). In this study, a high-quality mitochondrial genome sequence of *P. granatum* was sequenced and assembled. It had a multi branched structure, similar to the main structure of the mitochondrial genome of sitka spruce (*Picea sitchensis*) (Jackman et al., 2020). The length of *P. granatum* mitochondrial genome was 404,807 bp, and its genome size differed greatly from *Rhopalocnemis phalloides* (130,713 bp) (Yu et al., 2022), mango (*Mangifera indica*) (714.4~750.9 kb) (Niu et al., 2022), strawberry (*Fragaria*) (275.2~351.2 kb) (Fan et al., 2022). The GC content of *P. granatum* mitochondrial genome was 46.09%, which was very similar to those above species, indicating that the GC content of angiosperms was very conservative during evolution.

There are a large number of repetitive sequences in plant mitochondrial genome, among which SSR markers have important applications in species identification and genetic diversity evaluation (Khera et al., 2015). In this study, 146 SSRs were obtained from the mitochondrial genome of *P. granatum*, providing a large number of reference markers for the evaluation of pomegranate germplasm diversity and species identification. The results will be more accurate and reliable if molecular markers from nuclear gene and chloroplast gene were combined, since the evolution rate of mitochondrial sequence is slower than that of nuclear and chloroplast gene (Qiu et al., 2010). Dispersed repeats, also known as transposable elements (TE), can regulate gene expression and affect plant phenotype (Lisch, 2013), such as color variation in Japanese morning glory (Clegg and Durbin, 2000), fruit shape in tomato (Xiao et al., 2008), plant height and ear height in make (Li et al., 2020). In our study, a total of 400 pairs of dispersed repeats were detected in the mitochondrial genome of *P. granatum*, but their effects on the phenotypic traits of pomegranate need to be further studied.

In this study, we found 14 chloroplast genome homologous fragments in *P. granatum* mitochondrial genome, with a total length of 2,200 bp, accounting for 0.54% of the total mitochondrial genome length. The number and proportion of homologous fragments were slightly equal to mango (*Mangifera indica*) (7~10 homologous fragments, accounting for 0.51~0.61%) (Niu et al., 2022), but significantly less than sweet potato (*Ipomoea batatas*) (No.27, 7.35%) (Yang et al., 2022), *Suaeda glauca* (No.32, 5.18%) (Cheng et al., 2021), and *Tolypanthus maclurei* (No.31, 9.10%) (Yu et al., 2021). It may indicate that sequence migration from chloroplast varied greatly among different plant mitochondrial genomes. Among these 14 homologous sequences, seven were complete genes, all of which were tRNA genes, similar to the results of Cheng et al. (2021) and Yang et al. (2022). It should be noted that since most of these migration sequences lost their integrity during the evolutionary process, only some of them can be found in the mitochondrial genome now. However, compared with the sequence migration of protein coding genes, tRNA genes were more conservative in the mitochondrial genome, suggesting their indispensable roles in mitochondria.

For a long time, the scientific community has been controversial about the classification status of *Punica* genera (Berger et al., 2016). Previously, it was considered to belong to Punicaceae alone (Rana et al., 2010), but molecular (Berger et al., 2016; Yuan et al., 2018) and morphological (Graham and Graham, 2014) evidence in recent studies, as well as the new classification in the APG IV system (Byng et al., 2016), suggests that instead it should be a member of Lythraceae family in Myrtales. Here in our study, the analysis of mitochondrial genome evolution showed that *P. granatum* was closely related to *Lagerstroemia indica* of Lythraceae. Although there are only seven species or genera of the whole Myrtales, our study provides valuable resources for solving the previously disputed taxonomic location of *Punica* genera. In addition, it is demonstrated in collinearity analysis that the mitochondrial genome sequences of the seven species of Myrtle were extremely non-conservative in order, indicating that they have experienced extremely frequent genome recombination in the long evolutionary process, which also provides an effective way to understand species evolution.

In addition, this study also predicted RNA editing events in *P. granatum* mitochondrial genome. Based on the analysis of transcriptome sequences data, we found 580 RNA editing sites using BEDTools software, which were very similar to other terrestrial plants (Li et al., 2022; Yang et al., 2022; Yu et al., 2022). Besides, the RNA editing events discovered by the two methods were both C to U, which may affect the start or end points of PCG. Quiñones et al. (1995) discovered that the *cox1* starting codon was generated by RNA editing in potato (*Solanum tuberosum*) mitochondria, and the editing event from C to U occurred, which enabled ACG to be edited to AUG, and then used as the starting point of gene transcription. Surprisingly, Zandueta-Criado and Bock (2004) observed that ACG can be directly used as the starting point of transcription without editing in organelle genome. Moreover, we used the online website PREPACT to predict an editing event from ACG to AUG in the *cox2* and *nad7* genes respectively. However, whether it was due to the phenomenon that editing event was activated at the starting position, no support was obtained from transcriptome comparison reads, and further experimental verification was still needed.

## Conclusions

In this study, the mitochondrial genome of *P. granatum* was successfully sequenced and assembled by using BGI + Nanopore mixed assembly strategy. The *P. granatum* mitochondria were mainly multi branched, with a total genome length of 404,807 bp, and 37 unique PCGs were annotated. In the mitochondrial genome of *P. granatum*, 146 SSRs were identified. Besides, 400 pairs of dispersed repeats were detected, including 179 palindromic, 220 forward and one reverse. Interestingly, there were 14 homologous fragments with chloroplast genome, with a total length of 2,200 bp, accounting for only 0.54% of the total length of mitochondrial genome. Phylogenetic analysis demonstrated that *P. granatum* had the closest genetic relationship with *Lagerstroemia indica* of Lythraceae. The identified RNA editing events were all C-U editing, and one editing event from ACG to AUG was predicted in *cox2* and *nad7* genes respectively. This study provided a theoretical basis for understanding the evolution of higher plants and the utilization of pomegranate germplasm resources, and also proved the genetic diversity of plant mitochondrial genome.

## Data availability statement

The datasets presented in this study can be found in online repositories. The names of the repository/repositories and accession number(s) can be found in the article/**Supplementary Material**. The mitogenome sequence data of P. granatum are available in NCBI Nucleotide Database under the GenBank accessions: OP874592. The BGI and Nanopore sequencing data of P. granatum have been deposited in the Figshare platform: doi: 10.6084/m9.figshare.21599349. The raw transcriptome sequencing data of P. granatum have been submitted to the Sequence Read Archive (SRA) repository under SRR22890448. All the other mitogenome sequences used in this study are available in the NCBI Genome Database (https://www.ncbi.nlm.nih.gov/genome/organelle/) under the GenBank accessions listed in **Supplementary Table 1**.

## Author contributions

GL: Methodology, data curation, formal analysis, software, visualization, writing - original draft. KZ: Data curation, formal analysis. YQ: Conceptualization, methodology, visualization, supervision, writing - review & editing. YL: Conceptualization, funding acquisition, supervision, resources, project administration. All authors contributed to manuscript revision, read, and approved the submitted version. All authors contributed to the article and approved the submitted version.
