# Supplementary material for: Assembly and analysis of the first complete mitochondrial genome of Punica granatum and the gene transfer from chloroplast genome
Source: Front Plant Sci. 2023 Jun 21;14:1132551. doi: 10.3389/fpls.2023.1132551 (PMC10320729; doi:10.3389/fpls.2023.1132551)
Supplement: Supplementary file 2 [file DataSheet_2.pdf]

Supplementary Material

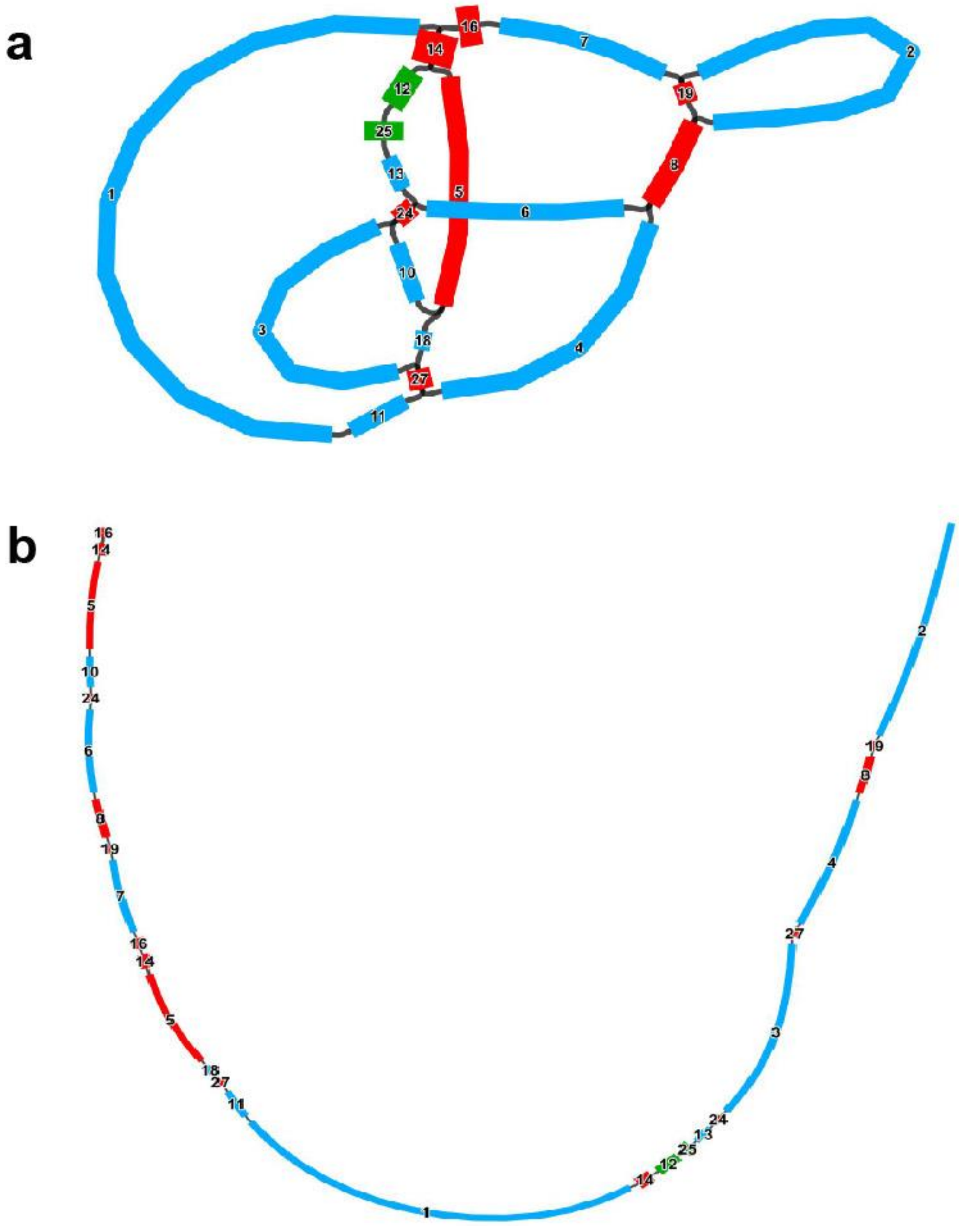

Figure S1. Sketch map of *Punica granatum* mitochondrial genome.

a. *Punica granatum* mitochondrial genome sketch; b. Expanded mitochondrial sketch.

Note: The red node is the predicted repeat region, the green node is the predicted sequence of chloroplast genome migration to mitochondrial genome, and the blue represents a linear contig single copy region obtained after the duplicate region is resolved based on Nanopore data. Path selection for each node (repeating area) based on Nanopore data, 2-19-8-4-27-3-24-13-25-12-14-1-11-27-18-5-14-16-7-19-8-6-24-10-5-14-16.

Table S1. Related genera of *Punica granatum* and their complete mitochondrial genome in GenBank

Order	Genus	GenBank
Myrtales	*Punica granatum*	-
	Eucalyptus grandis	NC_040010.1
	Lagerstroemia indica	NC_035616.1
	Oenothera biennis	MZ934756.1
	Oenothera villaricae	MZ934755.1
	Oenothera elata subsp. hookeri	MZ934757.1
	Medinilla magnifica	MT043351.1
Sapindales	*Mangifera longipes*	NC_060990.1
	Citrus unshiu	NC_057142.1
	Citrus maxima	NC_057143.1
	Sapindus mukorossi	NC_050850.1
	Spondias mombin	NC_045035.1
	Spondias tuberosa	NC_045036.1
	Citrus sinensis	NC_037463.1
	Toona ciliata	NC_065060.1
	Mangifera sylvatica	MZ751077.1
	Cotinus coggygria	MZ089853.1
	Acer miaotaiense	MZ636518.1
	Nitraria tangutorum	MK431824.1
	Peganum harmala	MK431826.1
	Acer truncatum	MZ318049.1
	Xanthoceras sorbifolium	MK333231.1
Malvales	*Aquilaria sinensis*	NC_054354.1
	Gossypium harknessii	NC_027407.1
	Hibiscus cannabinus	NC_035549.1
	Bombax ceiba	NC_038052.1
	Gossypium barbadense	NC_028254.1
	Theobroma grandiflorum	NC_066895.1
Zygophyllales	*Zygophyllum fabago*	MK431827.1
	Tribulus terrestris	MK431825.1

Table S2. Primer sequences used for RNA editing

**Gene Name-<br>Editing Site**	**Forward primer (5'-3')**	**Reverse primer (5'-3')**
atp9-223	TTGGAAACGTCTTCAGTT	CGGGGACTAGCTCTTTA
nad1-2	TACTTGGTGAAATGGGT	AGGTAGCTTGCTTACTCT
rps10-2	GAGTACAATCCGATGCC	AATCCAATCTTCCGTGT
rps10-331	GCAAGACTCGTTCGGATAA	AGCATGTGCCCATCACT

Table S3. Relative synonymous codon usage (RSCU) of each amino acid pair in the mitochondrial genome of *Punica granatum*

Amino	Codon 1 RSCU	Codon 2 RSCU	Codon 3 RSCU	Codon 4 RSCU	Codon 5 RSCU	Codon 6 RSCU
Ala	GCU	GCA	GCC	GCG		
	1.63	0.99	0.95	0.43		
Arg	AGA	CGA	CGU	AGG	CGG	CGC
	1.43	1.33	1.23	0.78	0.73	0.49
Asn	AAU	AAC				
	1.33	0.67				
Asp	GAU	GAC				
	1.37	0.63				
Cys	UGU	UGC				
	1.16	0.84				
End	UAA	UGA	UAG			
	2.18	0.55	0.27			
Gln	CAA	CAG				
	1.49	0.51				
Glu	GAA	GAG				
	1.36	0.64				
Gly	GGA	GGU	GGG	GGC		
	1.45	1.26	0.76	0.52		
His	CAU	CAC				
	1.53	0.47				
Ile	AUU	AUC	AUA			
	1.31	0.88	0.81			
Leu	UUA	CUU	UUG	CUA	CUC	CUG
	1.37	1.27	1.22	0.94	0.61	0.59
Lys	AAA	AAG				
	1.19	0.81				
Met	AUG					
	1					
Phe	UUU	UUC				
	1.11	0.89				

Pro	CCU	CCA	CCC	CCG		
	1.48	1.06	0.87	0.6		
Ser	UCU	UCA	AGU	UCC	UCG	AGC
	1.38	1.15	1.03	0.98	0.83	0.63
Thr	ACU	ACA	ACC	ACG		
	1.39	1.03	1	0.58		
Trp	UGG					
	1					
Tyr	UAU	UAC				
	1.49	0.51				
Val	GUU	GUA	GUG	GUC		
	1.18	1.17	0.87	0.78		

Table S4. SSRs in the mitochondrial genome of *Punica granatum*

SSR nr.	SSR type	SSR	size	start	end
1	p1	(A)10	10	5006	5015
2	p4	(TAAA)3	12	11878	11889
3	p5	(CTTCG)3	15	16051	16065
4	p3	(TCA)4	12	20340	20351
5	p3	(TTA)4	12	25701	25712
6	p1	(T)10	10	29854	29863
7	p2	(GA)5	10	40974	40983
8	p4	(CGCC)3	12	41291	41302
9	p3	(ATA)4	12	41483	41494
10	p4	(ATGA)3	12	48202	48213
11	p1	(T)12	12	51376	51387
12	p1	(T)10	10	54664	54673
13	p4	(ACTC)3	12	59986	59997
14	p4	(TCTA)3	12	60027	60038
15	p1	(T)10	10	61234	61243
16	p1	(T)10	10	62634	62643
17	p1	(A)10	10	63559	63568
18	p2	(AT)5	10	67277	67286
19	p1	(T)11	11	67772	67782
20	p4	(AGAT)3	12	68757	68768
21	p1	(A)10	10	71616	71625
22	p1	(G)10	10	74648	74657
23	p5	(ATTTC)3	15	78084	78098
24	p2	(TC)5	10	83187	83196
25	p4	(ATTC)3	12	83375	83386
26	p2	(TA)5	10	84613	84622
27	p4	(AGTA)4	16	87774	87789
28	p4	(GAAA)3	12	89864	89875
29	p2	(TC)5	10	91052	91061
30	p4	(ACAA)3	12	94129	94140
31	p1	(A)10	10	100390	100399
32	p4	(TAAA)3	12	107262	107273

33	p5	(CTTCG)3	15	111435	111449
34	p3	(TCA)4	12	115724	115735
35	p3	(TTA)4	12	121085	121096
36	p1	(T)10	10	125238	125247
37	p1	(T)13	13	129559	129571
38	p1	(G)12	12	130860	130871
39	p1	(T)10	10	136556	136565
40	p1	(A)10	10	136765	136774
41	p3	(CAT)4	12	140092	140103
42	p4	(AAGA)3	12	142266	142277
43	p4	(AAAG)3	12	142745	142756
44	p2	(TC)5	10	142888	142897
45	p6	(ACTCGG)3	18	148076	148093
46	p4	(GCTT)3	12	149194	149205
47	p2	(AG)5	10	151684	151693
48	p1	(A)10	10	153397	153406
49	p2	(CT)5	10	153533	153542
50	p1	(A)10	10	154382	154391
51	p1	(T)10	10	154463	154472
52	p1	(A)12	12	155008	155019
53	p4	(CTTT)3	12	155639	155650
54	p1	(T)14	14	156031	156044
55	p4	(TGAC)3	12	164630	164641
56	p3	(TAG)4	12	165903	165914
57	p6	(CCTTCG)3	18	166856	166873
58	p1	(A)10	10	167584	167593
59	p1	(T)10	10	167765	167774
60	p1	(A)10	10	168526	168535
61	p5	(GAAAT)3	15	168877	168891
62	p3	(AAG)4	12	169844	169855
63	p1	(T)13	13	171074	171086
64	p4	(TAAG)3	12	171384	171395
65	p3	(AGT)4	12	172372	172383
66	p4	(AAGT)3	12	172490	172501

67	p4	(GCCT)3	12	177466	177477
68	p1	(A)10	10	183130	183139
69	p1	(C)11	11	184666	184676
70	p5	(ACTTT)3	15	185465	185479
71	p1	(A)10	10	188108	188117
72	p1	(G)10	10	189310	189319
73	p1	(A)10	10	196731	196740
74	p1	(A)10	10	197726	197735
75	p1	(A)10	10	197966	197975
76	p4	(CTTT)3	12	201533	201544
77	p1	(A)12	12	206725	206736
78	p4	(CTTA)3	12	212997	213008
79	p5	(TTTTA)3	15	222386	222400
80	p1	(T)10	10	224336	224345
81	p1	(A)11	11	224371	224381
82	p1	(A)10	10	230238	230247
83	p1	(A)15	15	235601	235615
84	p2	(AG)5	10	238475	238484
85	p1	(T)10	10	239049	239058
86	p4	(AAGG)3	12	241875	241886
87	p1	(A)10	10	242753	242762
88	p3	(TTA)4	12	243826	243837
89	p2	(GA)5	10	244927	244936
90	p1	(T)11	11	245217	245227
91	p4	(TTTC)3	12	246252	246263
92	p3	(AAG)4	12	254050	254061
93	p1	(T)10	10	257075	257084
94	p3	(GAA)4	12	257985	257996
95	p1	(T)10	10	259945	259954
96	p4	(AATG)3	12	262875	262886
97	p1	(G)10	10	262956	262965
98	p2	(AT)5	10	265149	265158
99	p2	(CT)5	10	275032	275041
100	p1	(T)14	14	277924	277937

101	p3	(CAG)4	12	278611	278622
102	p1	(C)14	14	279846	279859
103	p2	(TA)7	14	283363	283376
104	p1	(T)11	11	283885	283895
105	p1	(A)10	10	287800	287809
106	p1	(A)11	11	288388	288398
107	p3	(TCC)5	15	293008	293022
108	p1	(T)11	11	301680	301690
109	p4	(TGAT)3	12	302859	302870
110	p5	(CAAGA)3	15	312912	312926
111	p4	(AAGC)3	12	313548	313559
112	p4	(GAGG)3	12	318458	318469
113	p5	(GAAAT)3	15	322586	322600
114	p2	(AT)5	10	328899	328908
115	p1	(T)11	11	329394	329404
116	p4	(AGAT)3	12	330379	330390
117	p1	(A)10	10	333238	333247
118	p1	(G)10	10	336270	336279
119	p2	(AT)5	10	343674	343683
120	p4	(TTAT)3	12	348393	348404
121	p2	(CG)5	10	350095	350104
122	p1	(A)10	10	354652	354661
123	p1	(A)11	11	357411	357421
124	p1	(A)10	10	358277	358286
125	p2	(TA)5	10	360270	360279
126	p4	(TAGC)3	12	362048	362059
127	p1	(C)11	11	362858	362868
128	p4	(ACCG)3	12	364008	364019
129	p4	(TCAT)3	12	365065	365076
130	p2	(GA)5	10	366696	366705
131	p3	(AGC)4	12	367328	367339
132	p3	(ATA)4	12	368290	368301
133	p1	(G)13	13	369262	369274
134	p2	(TA)5	10	369441	369450

135	p1	(T)13	13	372612	372624
136	p3	(GAA)4	12	372795	372806
137	p1	(T)10	10	376218	376227
138	p5	(TCAAG)5	25	377800	377824
139	p4	(TCGC)3	12	378932	378943
140	p1	(T)10	10	388725	388734
141	p1	(A)13	13	390013	390025
142	p4	(TGAA)3	12	390506	390517
143	p4	(GTCA)3	12	392710	392721
144	p4	(GCTG)3	12	392946	392957
145	p2	(GA)5	10	396138	396147
146	p3	(TTC)4	12	398946	398957

Table S5. Tandem repeat sequences in the mitochondrial genome of *Punica granatum*

Indices	Period Size	Copy Number	Consensus Size	Percent Matches	Percent Indels	Score	A	C	G	T	Entropy (0-2)
36479--36534	29	1.9	29	85	0	84	25	23	21	30	1.99
69874--69899	13	2	13	100	0	52	30	15	23	30	1.95
73404--73439	16	2.2	16	85	0	51	55	30	11	2	1.49
89504--89541	20	2	20	84	5	55	31	34	26	7	1.85
136808--136840	14	2.4	14	90	5	52	27	15	0	57	1.38
165910--165950	15	2.7	15	84	0	61	31	29	19	19	1.96
233699--233736	18	2.1	18	90	0	62	60	5	7	26	1.46
233693--233739	23	2	23	84	8	64	59	6	8	25	1.5
274364--274392	14	2.1	14	93	0	51	27	24	17	31	1.97
304836--304877	21	2	22	90	4	70	11	47	14	26	1.78
310642--310684	18	2.4	18	81	18	54	18	20	25	34	1.96
331496--331521	13	2	13	100	0	52	30	15	23	30	1.95
335026--335061	16	2.2	16	85	0	51	55	30	11	2	1.49
360577--360617	20	2	20	81	9	52	34	17	36	12	1.87
372618--372642	12	2.1	12	100	0	50	8	16	0	76	1.02
395312--395353	20	2.2	19	82	4	54	30	19	2	47	1.62

Table S6. Dispersed repeat sequences in the mitochondrial genome of *Punica granatum*

The repeat length of the first part	The starting site of the first part	Matching direction	The repeat length of the second part	The starting site of the second part	Interval distance of repeats	E-value
31296	1264	F	31296	96648	0	0.00E+00
10745	64384	F	10745	326006	0	0.00E+00
3726	3850	F	3726	229082	0	0.00E+00
3726	99234	F	3726	229082	0	0.00E+00
2073	75129	P	2073	402734	0	0.00E+00
318	41378	P	318	243624	0	1.62E-181
248	0	P	248	238602	0	2.25E-139
164	130214	F	164	290830	0	8.43E-89
125	57189	P	125	378907	-2	1.78E-60
110	14286	F	110	308494	0	2.74E-56
110	109670	F	110	308494	0	2.74E-56
109	307530	F	109	377575	-1	3.58E-53
114	243623	P	114	288645	-3	6.94E-52
113	41583	F	113	288645	-3	2.70E-51
101	307538	F	101	377583	0	7.17E-51
99	41597	F	99	288659	-2	5.01E-45
102	57177	P	102	378942	-3	8.31E-45
97	14559	P	97	86765	-2	7.69E-44
97	86765	P	97	109943	-2	7.69E-44
89	180030	F	89	268734	0	1.20E-43
88	237363	F	88	274435	0	4.81E-43
99	173397	F	99	292013	-3	4.86E-43
91	41605	F	91	288667	-1	2.05E-42
86	161653	P	86	224372	-1	1.99E-39
89	191555	F	89	384630	-2	4.24E-39
81	14575	P	81	86765	0	7.88E-39
84	84189	P	84	228114	-1	3.10E-38
78	52268	F	78	287269	0	5.05E-37
79	130674	P	79	236284	-1	2.99E-35
81	253821	F	81	344344	-2	2.30E-34

83	224263	F	83	376145	-3	1.22E-33
72	41753	F	72	147693	0	2.07E-33
79	224273	F	79	376155	-3	2.69E-31
77	136488	P	77	168525	-3	3.99E-30
75	53964	F	75	148537	-3	5.89E-29
75	130793	F	75	291856	-3	5.89E-29
63	41633	F	63	288695	0	5.42E-28
69	208501	F	69	295130	-2	2.79E-27
71	53947	F	71	148520	-3	1.28E-26
60	140592	P	60	287361	0	3.47E-26
67	213757	F	67	215773	-2	4.21E-26
70	6358	F	70	42853	-3	4.89E-26
70	42853	F	70	101742	-3	4.89E-26
70	42853	F	70	231590	-3	4.89E-26
63	348971	F	63	378417	-1	1.02E-25
59	52109	P	59	193901	0	1.39E-25
59	211752	F	59	343042	0	1.39E-25
62	136503	P	62	168525	-1	4.03E-25
57	51063	P	57	74152	0	2.22E-24
57	51063	P	57	335774	0	2.22E-24
55	71981	P	55	207259	0	3.55E-23
55	207259	P	55	333603	0	3.55E-23
62	48949	P	62	75277	-2	3.69E-23
62	48949	F	62	404597	-2	3.69E-23
62	84264	P	62	227131	-2	3.69E-23
65	6936	F	65	43427	-3	3.99E-23
65	43427	F	65	102320	-3	3.99E-23
65	43427	F	65	232168	-3	3.99E-23
65	206726	P	65	376162	-3	3.99E-23
54	39864	F	54	213622	0	1.42E-22
54	208516	F	54	295145	0	1.42E-22
57	192923	P	57	259177	-1	3.79E-22
60	15965	F	60	288255	-2	5.52E-22
60	111349	F	60	288255	-2	5.52E-22

53	84220	P	53	228114	0	5.68E-22
63	161551	P	63	224491	-3	5.81E-22
62	161536	P	62	224507	-3	2.21E-21
51	53822	F	51	148474	0	9.09E-21
50	92426	P	50	193789	0	3.64E-20
59	161449	P	59	224587	-3	1.22E-19
52	317364	F	52	319489	-1	3.54E-19
48	27372	P	48	204126	0	5.82E-19
48	122756	P	48	204126	0	5.82E-19
48	130705	P	48	236284	0	5.82E-19
51	224301	F	51	376183	-1	1.39E-18
46	173465	P	46	301211	0	9.31E-18
49	50937	P	49	187852	-1	2.14E-17
53	6392	F	53	42887	-3	3.59E-16
53	42887	F	53	101776	-3	3.59E-16
53	42887	F	53	231624	-3	3.59E-16
53	161419	P	53	224623	-3	3.59E-16
46	57268	P	46	378907	-1	1.28E-15
49	243688	P	49	288645	-2	1.54E-15
51	161692	P	51	376256	-3	5.11E-15
48	6953	F	48	43444	-2	5.91E-15
48	43444	F	48	102337	-2	5.91E-15
48	43444	F	48	232185	-2	5.91E-15
41	15355	F	41	75236	0	9.53E-15
41	15355	P	41	404659	0	9.53E-15
41	75236	F	41	110739	0	9.53E-15
41	110739	P	41	404659	0	9.53E-15
50	173482	F	50	292098	-3	1.92E-14
44	10412	P	44	323308	-1	1.97E-14
44	105796	P	44	323308	-1	1.97E-14
44	206726	P	44	224301	-1	1.97E-14
44	289968	F	44	349436	-1	1.97E-14
47	65740	P	47	293847	-2	2.26E-14
47	173327	F	47	287238	-2	2.26E-14

47	293847	P	47	327362	-2	2.26E-14
40	51030	F	40	51896	0	3.81E-14
40	338012	P	40	385290	0	3.81E-14
49	1411	P	49	311024	-3	7.23E-14
49	96795	P	49	311024	-3	7.23E-14
49	161436	P	49	224610	-3	7.23E-14
46	32558	F	46	128437	-2	8.67E-14
39	228217	F	39	228325	0	1.52E-13
39	390323	P	39	397915	0	1.52E-13
48	25497	F	48	180030	-3	2.72E-13
48	25497	F	48	268734	-3	2.72E-13
48	120881	F	48	180030	-3	2.72E-13
48	120881	F	48	268734	-3	2.72E-13
45	1629	P	45	310812	-2	3.32E-13
45	65569	F	45	72118	-2	3.32E-13
45	65569	F	45	333740	-2	3.32E-13
45	72118	F	45	327191	-2	3.32E-13
45	97013	P	45	310812	-2	3.32E-13
45	147647	P	45	372159	-2	3.32E-13
45	327191	F	45	333740	-2	3.32E-13
38	317378	F	38	319503	0	6.1E-13
47	75463	P	47	84858	-3	1.02E-12
47	84858	F	47	404426	-3	1.02E-12
41	84285	P	41	227131	-1	1.17E-12
41	91088	F	41	173801	-1	1.17E-12
44	41332	P	44	74171	-2	1.27E-12
44	41332	P	44	335793	-2	1.27E-12
44	365330	P	44	394333	-2	1.27E-12
37	241279	F	37	315667	0	2.44E-12
46	206711	P	46	376196	-3	3.81E-12
40	177650	P	40	364032	-1	4.57E-12
36	51111	F	36	349027	0	9.76E-12
36	136529	P	36	168525	0	9.76E-12
36	139801	F	36	141511	0	9.76E-12

36	242207	P	36	291939	0	9.76E-12
45	747	P	45	311676	-3	1.43E-11
45	224374	F	45	376262	-3	1.43E-11
39	149111	F	39	240289	-1	1.78E-11
39	293855	P	39	327362	-1	1.78E-11
35	51063	P	35	348988	0	3.9E-11
35	51063	P	35	378434	0	3.9E-11
35	57220	P	35	361836	0	3.9E-11
35	72016	F	35	308295	0	3.9E-11
35	74174	F	35	348988	0	3.9E-11
35	74174	F	35	378434	0	3.9E-11
35	90090	F	35	310236	0	3.9E-11
35	308295	F	35	333638	0	3.9E-11
35	335796	F	35	348988	0	3.9E-11
35	335796	F	35	378434	0	3.9E-11
35	361836	F	35	378966	0	3.9E-11
44	65460	P	44	207235	-3	5.33E-11
44	207235	P	44	327082	-3	5.33E-11
38	65721	F	38	287250	-1	6.95E-11
38	228199	F	38	376847	-1	6.95E-11
38	287250	F	38	327343	-1	6.95E-11
38	288667	F	38	391474	-1	6.95E-11
41	191641	F	41	384718	-2	7.03E-11
41	226266	F	41	278368	-2	7.03E-11
41	287035	F	41	341181	-2	7.03E-11
34	57280	P	34	378907	0	1.56E-10
34	202595	P	34	354114	0	1.56E-10
34	351565	F	34	388361	0	1.56E-10
43	161473	P	43	224579	-3	1.98E-10
43	173308	F	43	287219	-3	1.98E-10
40	41603	F	40	391472	-2	2.68E-10
40	243677	P	40	391472	-2	2.68E-10
37	41662	P	37	143139	-1	2.71E-10
37	74775	P	37	206607	-1	2.71E-10

37	147655	P	37	372159	-1	2.71E-10
37	176581	F	37	211686	-1	2.71E-10
37	206607	P	37	336397	-1	2.71E-10
33	240904	P	33	391539	0	6.25E-10
42	173497	F	42	292113	-3	7.39E-10
39	54000	F	39	148573	-2	1.02E-09
39	185306	P	39	223822	-2	1.02E-09
36	15989	F	36	288279	-1	1.05E-09
36	111373	F	36	288279	-1	1.05E-09
36	180996	P	36	247269	-1	1.05E-09
41	73190	F	41	228778	-3	2.74E-09
41	202657	P	41	303475	-3	2.74E-09
41	228778	F	41	334812	-3	2.74E-09
38	41338	F	38	51063	-2	3.86E-09
35	288768	F	35	391623	-1	4.1E-09
35	372810	F	35	372939	-1	4.1E-09
31	32573	F	31	128452	0	9.99E-09
31	42537	P	31	310473	0	9.99E-09
31	60944	F	31	156777	0	9.99E-09
31	65523	F	31	72072	0	9.99E-09
31	65523	F	31	333694	0	9.99E-09
31	65721	F	31	173339	0	9.99E-09
31	72072	F	31	327145	0	9.99E-09
31	171591	F	31	261416	0	9.99E-09
31	173339	F	31	327343	0	9.99E-09
31	228150	F	31	228291	0	9.99E-09
31	288625	F	31	309271	0	9.99E-09
31	327145	F	31	333694	0	9.99E-09
37	25508	F	37	180041	-2	1.46E-08
37	25508	F	37	268745	-2	1.46E-08
37	51991	P	37	146001	-2	1.46E-08
37	120892	F	37	180041	-2	1.46E-08
37	120892	F	37	268745	-2	1.46E-08
34	41338	P	34	348989	-1	1.59E-08

34	41338	P	34	378435	-1	1.59E-08
34	48977	P	34	75277	-1	1.59E-08
34	48977	F	34	404625	-1	1.59E-08
34	143142	F	34	243624	-1	1.59E-08
34	143142	P	34	288724	-1	1.59E-08
39	143152	P	39	380212	-3	3.76E-08
30	14293	P	30	207140	0	0.00000004
30	41667	P	30	42488	0	0.00000004
30	51971	F	30	203742	0	0.00000004
30	74726	P	30	347531	0	0.00000004
30	109677	P	30	207140	0	0.00000004
30	143317	P	30	309275	0	0.00000004
30	207140	P	30	308501	0	0.00000004
30	288675	F	30	391482	0	0.00000004
30	336348	P	30	347531	0	0.00000004
33	37321	P	33	302983	-1	6.18E-08
33	42641	F	33	131561	-1	6.18E-08
33	147646	F	33	180973	-1	6.18E-08
33	173397	P	33	199804	-1	6.18E-08
33	199804	P	33	292013	-1	6.18E-08
33	203729	F	33	391464	-1	6.18E-08
33	253869	F	33	344392	-1	6.18E-08
35	6245	F	35	42741	-2	0.000000209
35	42741	F	35	101629	-2	0.000000209
35	42741	F	35	231477	-2	0.000000209
35	57464	P	35	348981	-2	0.000000209
35	57464	P	35	378427	-2	0.000000209
35	288605	F	35	397898	-2	0.000000209
35	372865	F	35	372922	-2	0.000000209
32	5810	F	32	41907	-1	0.00000024
32	41907	F	32	101194	-1	0.00000024
32	41907	F	32	231042	-1	0.00000024
32	73199	F	32	228787	-1	0.00000024
32	180974	P	32	372172	-1	0.00000024

32	228787	F	32	334821	-1	0.00000024
32	350335	F	32	359772	-1	0.00000024
37	43727	P	37	72138	-3	0.000000512
37	43727	P	37	333760	-3	0.000000512
34	84871	F	34	404439	-2	0.000000788
34	143298	P	34	247284	-2	0.000000788
34	145238	P	34	377334	-2	0.000000788
34	161584	P	34	224487	-2	0.000000788
34	173505	F	34	292121	-2	0.000000788
34	199803	P	34	259244	-2	0.000000788
34	235762	F	34	251602	-2	0.000000788
31	15392	P	31	206508	-1	0.000000929
31	84206	P	31	228291	-1	0.000000929
31	110776	P	31	206508	-1	0.000000929
31	208564	F	31	295199	-1	0.000000929
36	6081	F	36	42223	-3	0.00000188
36	42223	F	36	101465	-3	0.00000188
36	42223	F	36	231313	-3	0.00000188
33	206758	P	33	376162	-2	0.00000297
33	213982	F	33	238017	-2	0.00000297
30	2876	P	30	309181	-1	0.0000036
30	5912	F	30	42056	-1	0.0000036
30	6791	F	30	43280	-1	0.0000036
30	10128	F	30	42735	-1	0.0000036
30	37348	P	30	302954	-1	0.0000036
30	41346	F	30	51071	-1	0.0000036
30	41346	P	30	74171	-1	0.0000036
30	41346	P	30	335793	-1	0.0000036
30	41613	F	30	391482	-1	0.0000036
30	42056	F	30	101296	-1	0.0000036
30	42056	F	30	231144	-1	0.0000036
30	42468	F	30	203679	-1	0.0000036
30	42488	F	30	143141	-1	0.0000036
30	42488	F	30	243623	-1	0.0000036

30	42488	P	30	288729	-1	0.0000036
30	42735	F	30	105512	-1	0.0000036
30	43280	F	30	102175	-1	0.0000036
30	43280	F	30	232023	-1	0.0000036
30	51027	P	30	361937	-1	0.0000036
30	51941	F	30	203717	-1	0.0000036
30	72014	F	30	213609	-1	0.0000036
30	98260	P	30	309181	-1	0.0000036
30	207249	P	30	327082	-1	0.0000036
30	213609	F	30	333636	-1	0.0000036
30	247266	F	30	341438	-1	0.0000036
30	249638	P	30	261207	-1	0.0000036
30	271183	F	30	373191	-1	0.0000036
35	143311	F	35	390314	-3	0.0000069
35	202917	F	35	248241	-3	0.0000069
32	41412	F	32	58653	-2	0.0000112
32	58653	P	32	243876	-2	0.0000112
32	130627	P	32	236376	-2	0.0000112
32	159791	F	32	199560	-2	0.0000112
32	215609	P	32	306580	-2	0.0000112
32	238327	F	32	372207	-2	0.0000112
34	6518	F	34	43009	-3	0.0000252
34	6527	F	34	43018	-3	0.0000252
34	43009	F	34	101902	-3	0.0000252
34	43009	F	34	231750	-3	0.0000252
34	43018	F	34	101911	-3	0.0000252
34	43018	F	34	231759	-3	0.0000252
34	171678	P	34	368765	-3	0.0000252
34	173396	F	34	259243	-3	0.0000252
31	12735	P	31	303324	-2	0.0000418
31	22384	F	31	321275	-2	0.0000418
31	51070	F	31	57464	-2	0.0000418
31	51896	P	31	361933	-2	0.0000418
31	57464	P	31	74171	-2	0.0000418

31	57464	P	31	335793	-2	0.0000418
31	108119	P	31	303324	-2	0.0000418
31	117768	F	31	321275	-2	0.0000418
31	143151	F	31	243633	-2	0.0000418
31	143151	P	31	288718	-2	0.0000418
31	262740	P	31	378151	-2	0.0000418
31	348522	F	31	367490	-2	0.0000418
33	1439	P	33	311012	-3	0.000092
33	6118	F	33	42260	-3	0.000092
33	42260	F	33	101502	-3	0.000092
33	42260	F	33	231350	-3	0.000092
33	96823	P	33	311012	-3	0.000092
33	143313	P	33	397928	-3	0.000092
33	259244	F	33	292013	-3	0.000092
33	292094	P	33	301211	-3	0.000092
33	395771	R	33	395781	-3	0.000092
30	32340	P	30	194105	-2	0.000157
30	41823	P	30	203648	-2	0.000157
30	44415	P	30	68812	-2	0.000157
30	44415	P	30	330434	-2	0.000157
30	48525	P	30	200774	-2	0.000157
30	53429	P	30	350734	-2	0.000157
30	54010	F	30	148583	-2	0.000157
30	54043	P	30	379086	-2	0.000157
30	54338	P	30	262737	-2	0.000157
30	58615	F	30	92449	-2	0.000157
30	127724	P	30	194105	-2	0.000157
30	161486	P	30	224579	-2	0.000157
30	161713	P	30	376256	-2	0.000157
30	168122	F	30	193943	-2	0.000157
30	202861	P	30	389610	-2	0.000157
30	208739	P	30	363043	-2	0.000157
30	213611	F	30	308295	-2	0.000157
30	243688	P	30	391471	-2	0.000157

30	243914	F	30	391476	-2	0.000157
30	314000	P	30	353729	-2	0.000157
30	361904	F	30	372129	-2	0.000157
32	41346	F	32	57465	-3	0.000335
32	41346	P	32	348983	-3	0.000335
32	41346	P	32	378429	-3	0.000335
32	52304	F	32	341493	-3	0.000335
32	84881	F	32	404449	-3	0.000335
32	209452	P	32	221300	-3	0.000335
32	287305	F	32	341493	-3	0.000335
31	2891	P	31	309165	-3	0.00121
31	3172	P	31	308753	-3	0.00121
31	16000	F	31	288290	-3	0.00121
31	41378	P	31	391473	-3	0.00121
31	50351	F	31	286241	-3	0.00121
31	53356	F	31	249888	-3	0.00121
31	54730	F	31	250705	-3	0.00121
31	57226	F	31	139247	-3	0.00121
31	58667	P	31	370578	-3	0.00121
31	68665	F	31	338030	-3	0.00121
31	85590	F	31	321069	-3	0.00121
31	98275	P	31	309165	-3	0.00121
31	98556	P	31	308753	-3	0.00121
31	111384	F	31	288290	-3	0.00121
31	139247	P	31	378964	-3	0.00121
31	144290	F	31	378320	-3	0.00121
31	167987	P	31	255871	-3	0.00121
31	180982	P	31	372165	-3	0.00121
31	226279	F	31	278381	-3	0.00121
31	292081	P	31	301226	-3	0.00121
31	330287	F	31	338030	-3	0.00121
30	920	P	30	311518	-3	0.00438
30	2575	P	30	309512	-3	0.00438
30	6109	F	30	42251	-3	0.00438

30	7234	F	30	43766	-3	0.00438
30	10452	P	30	323281	-3	0.00438
30	11555	F	30	12353	-3	0.00438
30	11555	F	30	107737	-3	0.00438
30	12353	F	30	106939	-3	0.00438
30	24016	P	30	324719	-3	0.00438
30	42251	F	30	101493	-3	0.00438
30	42251	F	30	231341	-3	0.00438
30	43766	F	30	102618	-3	0.00438
30	43766	F	30	232466	-3	0.00438
30	58615	P	30	193786	-3	0.00438
30	58682	F	30	140194	-3	0.00438
30	65612	P	30	193993	-3	0.00438
30	97959	P	30	309512	-3	0.00438
30	105836	P	30	323281	-3	0.00438
30	106939	F	30	107737	-3	0.00438
30	119400	P	30	324719	-3	0.00438
30	139239	P	30	361843	-3	0.00438
30	143307	P	30	203711	-3	0.00438
30	143320	P	30	288626	-3	0.00438
30	145904	P	30	287924	-3	0.00438
30	163696	F	30	237380	-3	0.00438
30	163696	F	30	274452	-3	0.00438
30	193995	P	30	327232	-3	0.00438
30	206712	P	30	224327	-3	0.00438
30	224351	F	30	376240	-3	0.00438
30	243914	F	30	288669	-3	0.00438
30	353021	F	30	402428	-3	0.00438
30	359532	P	30	361907	-3	0.00438
30	370354	F	30	389045	-3	0.00438

Table S7. The homologous DNA fragment in the mitochondrial genome of *Punica granatum*.

Number	Identity %	Alignment Length	Mismatches	Gap Openings	Alignment start (chloroplast genome)	Alignment end (chloroplast genome)	Alignment start (mitochondrial genome)	Alignment end (mitochondrial genome)	E-value	Bit Score	MTPT annotation
1	92.039	716	48	7	37593	38303	2.83E+05	284104	0.00E+00	998	Complete (*trn*S-UGA), Partial (*psb*C)
2	93.711	159	9	1	70783	70941	181715	181558	2.1E-61	237	Complete (*trn*W-CCA)
3	82.456	285	29	11	32154	32422	156103	155824	3.5E-59	230	Complete (*trn*D-GUC)
4	92.053	151	12	0	71021	71171	181501	181351	3.48E-54	213	Complete (*trn*P-UGG)
5	83.902	205	33	0	131048	131252	355828	356032	3.51E-49	196	Partial (*ycf*1)
6	97.619	84	2	0	134567	134650	391047	390964	1.29E-33	145	Complete (*trn*N-GUU)
7	97.619	84	2	0	113010	113093	390964	391047	1.29E-33	145	Complete (*trn*N-GUU)
8	96.471	85	2	1	20	104	241304	241221	6.00E-32	139	Complete (*trn*H-GUG)
9	93.827	81	4	1	133370	133449	71817	71737	2.17E-26	121	Partial (*ycf*1)
10	93.827	81	4	1	114211	114290	71737	71817	2.17E-26	121	IGS(*trn*N-GUU, *ndh*F)
11	93.827	81	4	1	133370	133449	333439	333359	2.17E-26	121	Partial (*ycf*1)
12	93.827	81	4	1	114211	114290	333359	333439	2.17E-26	121	IGS(*trn*N-GUU, *ndh*F)
13	92.405	79	5	1	56030	56108	244630	244707	1.31E-23	111	Complete (*trn*M-CAU)
14	100	28	0	0	44067	44094	90154	90127	8.04E-06	52.8	Partial (*psa*A)

Table S8. Sequence collinearity of the mitochondrial genome of *Punica granatum* and related genera

query	database	identity (%)	alignment	mismatch	gap	q. start	q. end	d. start	d. end	e-value	score
MZ934756.1	MZ934757.1	99.996	102837	3	1	1	102836	1	102837	0	1.90E+05
MZ934756.1	MZ934757.1	99.996	91291	4	0	308018	399308	245204	336494	0	1.69E+05
MZ934756.1	MZ934757.1	99.99	70387	3	1	201759	272141	205923	135537	0	1.30E+05
MZ934756.1	MZ934757.1	99.997	67713	2	0	123050	190762	424392	356680	0	1.25E+05
MZ934756.1	MZ934757.1	100	32885	0	0	46165	79049	102831	135715	0	60728
MZ934756.1	MZ934757.1	99.992	24998	2	0	399135	424132	424219	449216	0	46152
MZ934756.1	MZ934757.1	99.99	19505	0	2	288309	307811	235082	215578	0	36007
MZ934756.1	MZ934757.1	99.994	17162	1	0	106062	123223	353482	336321	0	31687
MZ934756.1	MZ934757.1	99.973	15094	3	1	273628	288720	220605	205512	0	27850
MZ934756.1	MZ934757.1	99.974	7652	2	0	194519	202170	242322	234671	0	14120
MZ934756.1	MZ934757.1	99.618	3399	8	2	191429	194827	356572	353179	0	6200
MZ934756.1	MZ934757.1	99.625	3202	7	2	103169	106365	245215	242014	0	5842
MZ934756.1	MZ934757.1	100	1906	0	0	272135	274040	6018	4113	0	3520
MZ934756.1	MZ934757.1	99.779	906	2	0	1	906	356094	356999	0	1663
MZ934756.1	MZ934757.1	73.272	1201	237	58	46954	48111	40648	41807	9.70E-99	363
MZ934756.1	MZ934757.1	73.272	1201	237	60	40647	41806	46955	48112	9.70E-99	363
MZ934756.1	MZ934757.1	73.272	1201	237	60	40647	41806	103620	104777	9.70E-99	363
MZ934757.1	MZ934755.1	99.642	85726	110	56	250862	336494	298281	383902	0.00E+00	1.56E+05

MZ934757.1	MZ934755.1	99.685	79646	84	32	5	79596	1	79533	0.00E+00	1.46E+05
MZ934757.1	MZ934755.1	99.799	32886	13	9	102831	135704	46177	79021	0.00E+00	60314
MZ934757.1	MZ934755.1	99.708	32204	26	14	159078	191262	229854	197700	0.00E+00	58887
MZ934757.1	MZ934755.1	99.8	25017	24	9	424219	449216	383729	408738	0.00E+00	45897
MZ934757.1	MZ934755.1	99.658	22518	41	10	222710	245215	131055	108562	0.00E+00	41124
MZ934757.1	MZ934755.1	99.591	20067	31	13	82794	102837	88186	108224	0.00E+00	36555
MZ934757.1	MZ934755.1	99.561	19813	40	14	387569	407358	163954	183742	0.00E+00	36062
MZ934757.1	MZ934755.1	99.74	19643	20	11	356680	376310	260497	280120	0.00E+00	35962
MZ934757.1	MZ934755.1	99.804	14809	23	2	144015	158817	244650	229842	0.00E+00	27181
MZ934757.1	MZ934755.1	99.851	10075	9	1	205512	215586	131057	141125	0.00E+00	18517
MZ934757.1	MZ934755.1	99.386	8960	21	8	336347	345284	280392	289339	0.00E+00	16211
MZ934757.1	MZ934755.1	99.656	7263	9	5	349320	356572	252572	259828	0.00E+00	13260
MZ934757.1	MZ934755.1	99.822	6723	6	2	409766	416482	150950	157672	0.00E+00	12344
MZ934757.1	MZ934755.1	99.476	6484	14	3	416534	423013	157673	164140	0.00E+00	11767
MZ934757.1	MZ934755.1	98.245	6723	68	8	135710	142424	296862	290182	0.00E+00	11716
MZ934757.1	MZ934755.1	99.541	6103	3	3	245204	251306	190410	184333	0.00E+00	11092
MZ934757.1	MZ934755.1	99.617	5476	9	3	382287	387757	88194	82726	0.00E+00	9985
MZ934757.1	MZ934755.1	99.796	3915	6	2	345403	349315	248710	252624	0.00E+00	7184
MZ934757.1	MZ934755.1	99.932	2943	1	1	215578	218519	190617	193559	0.00E+00	5422
MZ934757.1	MZ934755.1	99.539	2602	6	1	407227	409828	144260	141665	0.00E+00	4734

MZ934757.1	MZ934755.1	99.313	1602	9	2	142425	144025	246947	245347	0.00E+00	2896
MZ934757.1	MZ934755.1	98.582	1410	6	3	422820	424228	82907	81511	0.00E+00	2481
MZ934757.1	MZ934755.1	99.667	902	3	0	356098	356999	1	902	0.00E+00	1650
MZ934757.1	MZ934755.1	92.391	828	54	3	141598	142424	247734	246915	0.00E+00	1171
MZ934757.1	MZ934755.1	97.842	556	10	2	142425	142979	290214	289660	0.00E+00	959
MZ934757.1	MZ934755.1	73.272	1201	237	58	46955	48112	40655	41814	9.35E-99	363
MZ934757.1	MZ934755.1	73.272	1201	237	58	103620	104777	40655	41814	9.35E-99	363
MZ934757.1	MZ934755.1	73.272	1201	237	60	40648	41807	46966	48123	9.35E-99	363
MZ934755.1	mtDNA	95.246	7594	217	55	401189	408721	355077	347567	0.00E+00	11891
MZ934755.1	mtDNA	93.39	5174	183	62	224490	229543	393490	388356	0.00E+00	7513
MZ934755.1	mtDNA	94.58	4889	180	34	254998	259828	53873	49012	0.00E+00	7481
MZ934755.1	mtDNA	95.785	4555	133	30	101425	105947	263439	258912	0.00E+00	7293
MZ934755.1	mtDNA	98.395	3240	39	4	74979	78209	15232	18467	0.00E+00	5683
MZ934755.1	mtDNA	98.395	3240	39	4	74979	78209	110616	113851	0.00E+00	5683
MZ934755.1	mtDNA	96.583	3278	81	16	373501	376758	25844	29110	0.00E+00	5404
MZ934755.1	mtDNA	96.583	3278	81	16	373501	376758	121228	124494	0.00E+00	5404
MZ934755.1	mtDNA	92.276	3651	160	48	32748	36319	364673	361066	0.00E+00	5068
MZ934755.1	mtDNA	96.004	3103	63	20	270558	273616	150255	153340	0.00E+00	4987
MZ934755.1	mtDNA	95.174	3170	106	17	230151	233292	388368	385218	0.00E+00	4963
MZ934755.1	mtDNA	96.301	3001	74	15	134781	137759	205822	202837	0.00E+00	4892

MZ934755.1	mtDNA	96.289	2964	90	11	265236	268191	144774	147725	0.00E+00	4846
MZ934755.1	mtDNA	95.353	2690	78	23	211057	213716	291466	288794	0.00E+00	4231
MZ934755.1	mtDNA	96.694	2420	61	8	6318	8720	248924	251341	0.00E+00	4008
MZ934755.1	mtDNA	92.846	2544	96	27	221699	224194	396542	394037	0.00E+00	3611
MZ934755.1	mtDNA	94.39	2353	52	30	71770	74074	44126	41806	0.00E+00	3541
MZ934755.1	mtDNA	96.834	2116	20	6	187660	189739	2255	151	0.00E+00	3493
MZ934755.1	mtDNA	95.21	2171	66	21	174517	176659	280480	282640	0.00E+00	3398
MZ934755.1	mtDNA	98.175	1808	29	3	201085	202892	161936	163739	0.00E+00	3153
MZ934755.1	mtDNA	98.434	1660	23	2	131461	133120	336412	338068	0.00E+00	2918
MZ934755.1	mtDNA	95.128	1868	57	11	214355	216203	288135	286283	0.00E+00	2915
MZ934755.1	mtDNA	93.491	1813	96	12	384926	386735	139240	141033	0.00E+00	2675
MZ934755.1	mtDNA	96.056	1648	54	9	112470	114115	403750	402112	0.00E+00	2673
MZ934755.1	mtDNA	99.43	1403	8	0	52916	54318	310582	311984	0.00E+00	2547
MZ934755.1	mtDNA	92.448	1814	73	32	336675	338459	59777	57999	0.00E+00	2532
MZ934755.1	mtDNA	95.688	1577	53	4	216374	217936	285939	284364	0.00E+00	2521
MZ934755.1	mtDNA	95.548	1550	66	2	352100	353649	127843	129389	0.00E+00	2477
MZ934755.1	mtDNA	95.814	1529	49	8	180860	182377	228202	226678	0.00E+00	2455
MZ934755.1	mtDNA	94.161	1610	59	11	286309	287907	235584	233999	0.00E+00	2420
MZ934755.1	mtDNA	98.397	1372	11	5	51527	52893	308997	310362	0.00E+00	2401
MZ934755.1	mtDNA	94.181	1581	60	9	178289	179849	277931	279499	0.00E+00	2381

MZ934755.1	mtDNA	93.989	1597	38	12	287985	289542	234003	232426	0.00E+00	2364
MZ934755.1	mtDNA	95.31	1450	35	17	269043	270475	148617	150050	0.00E+00	2270
MZ934755.1	mtDNA	91.63	1577	88	19	18599	20156	240478	242029	0.00E+00	2141
MZ934755.1	mtDNA	92.603	1433	61	14	116417	117804	191630	193062	0.00E+00	2017
MZ934755.1	mtDNA	91.734	1476	68	18	17126	18578	239043	240487	0.00E+00	2001
MZ934755.1	mtDNA	98.765	1053	13	0	393775	394827	6693	5641	0.00E+00	1873
MZ934755.1	mtDNA	98.765	1053	13	0	393775	394827	102077	101025	0.00E+00	1873
MZ934755.1	mtDNA	94.481	1232	41	9	390943	392156	180035	181257	0.00E+00	1873
MZ934755.1	mtDNA	98.765	1053	13	0	393775	394827	231925	230873	0.00E+00	1873
MZ934755.1	mtDNA	92.923	1201	54	11	20602	21787	242230	243414	0.00E+00	1718
MZ934755.1	mtDNA	96.949	1016	30	1	112470	113484	76187	77202	0.00E+00	1703
MZ934755.1	mtDNA	96.872	991	6	2	187660	188625	97639	96649	0.00E+00	1635
MZ934755.1	mtDNA	95.866	1016	27	6	220703	221709	397574	396565	0.00E+00	1629
MZ934755.1	mtDNA	96.829	946	19	1	156552	157497	198805	199739	0.00E+00	1570
MZ934755.1	mtDNA	93.939	1056	35	12	264168	265202	143601	144648	0.00E+00	1568
MZ934755.1	mtDNA	95.825	958	29	4	383793	384749	138286	139233	0.00E+00	1537
MZ934755.1	mtDNA	95.638	894	36	1	1	894	49486	48596	0.00E+00	1432
MZ934755.1	mtDNA	89.379	1111	61	15	253939	255014	55024	53936	0.00E+00	1345
MZ934755.1	mtDNA	96.509	802	26	2	366535	367334	269661	268860	0.00E+00	1325
MZ934755.1	mtDNA	96.382	774	16	6	253163	253924	235717	236490	0.00E+00	1264

MZ934755.1	mtDNA	92.713	892	33	11	182813	183693	226686	225816	0.00E+00	1258
MZ934755.1	mtDNA	94.395	785	22	6	22301	23085	244058	244820	0.00E+00	1186
MZ934755.1	mtDNA	91.249	857	51	16	123119	123962	238460	237615	0.00E+00	1146
MZ934755.1	mtDNA	92.067	832	37	13	204253	205078	164765	165573	0.00E+00	1144
MZ934755.1	mtDNA	94.694	735	37	2	298688	299420	376231	376965	0.00E+00	1140
MZ934755.1	mtDNA	93.817	744	32	4	268258	268987	147746	148489	0.00E+00	1107
MZ934755.1	mtDNA	97.756	624	10	1	197089	197712	2389	3008	0.00E+00	1072
MZ934755.1	mtDNA	97.756	624	10	1	197089	197712	97773	98392	0.00E+00	1072
MZ934755.1	mtDNA	95.289	658	12	8	335706	336354	60415	59768	0.00E+00	1026
MZ934755.1	mtDNA	96.053	608	24	0	308863	309470	189321	188714	0.00E+00	990
MZ934755.1	mtDNA	90.959	730	50	5	325221	325945	372493	373211	0.00E+00	968
MZ934755.1	mtDNA	92.388	670	22	4	203034	203674	163732	164401	0.00E+00	928
MZ934755.1	mtDNA	95.304	575	27	0	281290	281864	225801	225227	0.00E+00	913
MZ934755.1	mtDNA	91.145	655	33	9	274209	274863	153684	154313	0.00E+00	865
MZ934755.1	mtDNA	90.923	661	24	17	380290	380949	172607	173232	0.00E+00	856
MZ934755.1	mtDNA	91.681	589	40	6	118189	118777	193132	193711	0.00E+00	808
MZ934755.1	mtDNA	90.302	629	40	17	50841	51465	308363	308974	0.00E+00	804
MZ934755.1	mtDNA	92.74	551	22	5	213802	214334	288678	288128	0.00E+00	780
MZ934755.1	mtDNA	91.124	507	30	3	186154	186646	306268	305763	0.00E+00	673
MZ934755.1	mtDNA	88.65	511	38	13	392215	392709	181254	181760	1.27E-171	604

MZ934755.1	mtDNA	74.128	889	175	41	72367	73225	7059	6196	6.65E-85	316
MZ934755.1	mtDNA	74.128	889	175	41	72367	73225	102443	101580	6.65E-85	316
MZ934755.1	mtDNA	74.128	889	175	41	72367	73225	232291	231428	6.65E-85	316
mtDNA	NC035616.1	97.831	3411	33	14	2238	5642	238843	242218	0.00E+00	5851
mtDNA	NC035616.1	97.831	3411	33	14	97622	101026	238843	242218	0.00E+00	5851
mtDNA	NC035616.1	97.729	2818	20	16	149225	152004	289398	292209	0.00E+00	4809
mtDNA	NC035616.1	98.566	2719	17	9	348992	351702	163704	166408	0.00E+00	4785
mtDNA	NC035616.1	98.504	2607	30	3	248827	251433	229032	226435	0.00E+00	4590
mtDNA	NC035616.1	97.549	2693	21	17	388210	390883	304091	301425	0.00E+00	4566
mtDNA	NC035616.1	99.035	2384	20	3	259672	262054	230451	232832	0.00E+00	4272
mtDNA	NC035616.1	97.927	2460	25	7	231560	234003	333948	331499	0.00E+00	4237
mtDNA	NC035616.1	98.771	2279	23	3	335800	338073	125906	128184	0.00E+00	4048
mtDNA	NC035616.1	99.243	2245	10	3	16384	18626	118011	120250	0.00E+00	4045
mtDNA	NC035616.1	99.243	2245	10	3	111768	114010	118011	120250	0.00E+00	4045
mtDNA	NC035616.1	97.427	2371	23	9	288648	290989	66357	68718	0.00E+00	4006
mtDNA	NC035616.1	98.763	2263	7	8	41727	43974	245990	243734	0.00E+00	4004
mtDNA	NC035616.1	99.005	2212	16	2	375460	377670	87793	89999	0.00E+00	3958
mtDNA	NC035616.1	98.889	1891	9	5	286609	288495	64247	66129	0.00E+00	3365
mtDNA	NC035616.1	95.632	2106	69	15	127791	129887	149770	147679	0.00E+00	3358
mtDNA	NC035616.1	96.529	1988	30	14	161890	163852	237446	235473	0.00E+00	3253

mtDNA	NC035616.1	98.738	1822	6	4	14541	16362	116149	117953	0.00E+00	3221
mtDNA	NC035616.1	98.738	1822	6	4	109925	111746	116149	117953	0.00E+00	3221
mtDNA	NC035616.1	98.244	1765	17	3	402028	403787	29713	31468	0.00E+00	3075
mtDNA	NC035616.1	99.009	1715	6	3	386486	388194	305847	304138	0.00E+00	3062
mtDNA	NC035616.1	99.405	1681	10	0	310412	312092	262051	260371	0.00E+00	3049
mtDNA	NC035616.1	97.051	1797	24	11	229083	230874	240446	242218	0.00E+00	2998
mtDNA	NC035616.1	96.44	1798	28	17	180002	181771	10975	9186	0.00E+00	2933
mtDNA	NC035616.1	98.826	1619	9	4	191461	193079	256667	258275	0.00E+00	2876
mtDNA	NC035616.1	98.373	1598	13	6	280349	281945	189489	187904	0.00E+00	2795
mtDNA	NC035616.1	98.791	1572	13	2	172817	174387	210243	211809	0.00E+00	2793
mtDNA	NC035616.1	97.263	1498	18	5	58732	60225	299786	298308	0.00E+00	2518
mtDNA	NC035616.1	96.308	1544	20	11	378438	379963	90857	92381	0.00E+00	2501
mtDNA	NC035616.1	95.669	1547	40	18	49563	51095	25445	23912	0.00E+00	2460
mtDNA	NC035616.1	99.554	1346	5	1	234274	235619	726	2070	0.00E+00	2451
mtDNA	NC035616.1	95.382	1559	20	14	369917	371435	329583	331129	0.00E+00	2433
mtDNA	NC035616.1	97.604	1419	23	5	359100	360517	322112	323520	0.00E+00	2422
mtDNA	NC035616.1	99.171	1327	9	1	152016	153342	292258	293582	0.00E+00	2388
mtDNA	NC035616.1	99.542	1310	6	0	198452	199761	129802	128493	0.00E+00	2386
mtDNA	NC035616.1	99.233	1303	5	1	203050	204352	95016	96313	0.00E+00	2346
mtDNA	NC035616.1	99.61	1283	5	0	225172	226454	131384	132666	0.00E+00	2342

mtDNA	NC035616.1	98.824	1275	12	2	226928	228202	134099	135370	0.00E+00	2268
mtDNA	NC035616.1	99.351	1233	3	1	101712	102944	333948	332721	0.00E+00	2228
mtDNA	NC035616.1	99.351	1233	3	1	6328	7560	333948	332721	0.00E+00	2228
mtDNA	NC035616.1	99.748	1191	3	0	52632	53822	22228	21038	0.00E+00	2183
mtDNA	NC035616.1	97.408	1273	16	5	352993	354264	167810	169066	0.00E+00	2152
mtDNA	NC035616.1	99.911	1122	1	0	309282	310403	263221	262100	0.00E+00	2067
mtDNA	NC035616.1	99.123	1140	9	1	367473	368612	326761	327899	0.00E+00	2049
mtDNA	NC035616.1	99.638	1104	4	0	139203	140306	58199	59302	0.00E+00	2017
mtDNA	NC035616.1	99.724	1087	3	0	396489	397575	322060	320974	0.00E+00	1991
mtDNA	NC035616.1	98.048	1127	13	8	188603	189728	254378	255496	0.00E+00	1951
mtDNA	NC035616.1	97.548	1101	2	3	239028	240107	253422	252326	0.00E+00	1860
mtDNA	NC035616.1	98.575	1053	11	1	76150	77202	31468	30420	0.00E+00	1858
mtDNA	NC035616.1	98	1050	7	7	51103	52143	23838	22794	0.00E+00	1810
mtDNA	NC035616.1	99.494	989	5	0	160400	161388	122622	121634	0.00E+00	1799
mtDNA	NC035616.1	96.445	1069	17	9	57661	58719	300889	299832	0.00E+00	1744
mtDNA	NC035616.1	99.358	934	6	0	268735	269668	191464	190531	0.00E+00	1692
mtDNA	NC035616.1	97.951	976	5	5	372493	373454	42410	41436	0.00E+00	1677
mtDNA	NC035616.1	99.564	918	4	0	352062	352979	166850	167767	0.00E+00	1674
mtDNA	NC035616.1	98.015	957	14	3	74178	75129	125906	126862	0.00E+00	1657
mtDNA	NC035616.1	96.256	1015	17	11	54538	55531	20237	19223	0.00E+00	1644

mtDNA	NC035616.1	98.06	928	8	2	244291	245214	249031	248110	0.00E+00	1605
mtDNA	NC035616.1	99.419	861	5	0	83566	84426	298029	297169	0.00E+00	1563
mtDNA	NC035616.1	99.877	816	1	0	73355	74170	125017	125832	0.00E+00	1502
mtDNA	NC035616.1	99.877	816	1	0	334977	335792	125017	125832	0.00E+00	1502
mtDNA	NC035616.1	98.472	851	7	6	48594	49443	26255	25410	0.00E+00	1495
mtDNA	NC035616.1	97.9	857	15	1	235626	236479	2119	2975	0.00E+00	1480
mtDNA	NC035616.1	99.873	786	1	0	122713	123498	181217	180432	0.00E+00	1447
mtDNA	NC035616.1	99.873	786	1	0	27329	28114	181217	180432	0.00E+00	1447
mtDNA	NC035616.1	99.747	790	1	1	143504	144292	285731	286520	0.00E+00	1447
mtDNA	NC035616.1	98.415	820	9	2	140556	141375	59673	60488	0.00E+00	1439
mtDNA	NC035616.1	98.515	808	10	1	284913	285720	62438	63243	0.00E+00	1424
mtDNA	NC035616.1	98.753	802	6	4	145258	146056	287265	288065	0.00E+00	1423
mtDNA	NC035616.1	98.731	788	10	0	262066	262853	232916	233703	0.00E+00	1400
mtDNA	NC035616.1	94.574	903	38	9	40426	41321	247226	246328	0.00E+00	1386
mtDNA	NC035616.1	99.332	748	4	1	141384	142130	60535	61282	0.00E+00	1352
mtDNA	NC035616.1	98.935	751	3	2	285738	286488	63303	64048	0.00E+00	1338
mtDNA	NC035616.1	96.556	813	6	5	243419	244226	249965	249170	0.00E+00	1327
mtDNA	NC035616.1	94.263	889	9	10	369048	369904	328448	329326	0.00E+00	1321
mtDNA	NC035616.1	98.643	737	3	1	146060	146796	288121	288850	0.00E+00	1299
mtDNA	NC035616.1	95.198	833	10	7	153907	154731	294487	295297	0.00E+00	1290

mtDNA	NC035616.1	97.538	731	11	3	130396	131125	68783	69507	0.00E+00	1243
mtDNA	NC035616.1	98.31	710	8	1	313398	314107	61336	62041	0.00E+00	1242
mtDNA	NC035616.1	98.849	695	7	1	53834	54527	20990	20296	0.00E+00	1238
mtDNA	NC035616.1	96.913	745	5	3	204365	205109	96359	97085	0.00E+00	1232
mtDNA	NC035616.1	99.12	682	3	2	230881	231559	682	1	0.00E+00	1223
mtDNA	NC035616.1	99.12	682	3	2	101033	101711	682	1	0.00E+00	1223
mtDNA	NC035616.1	99.12	682	3	2	5649	6327	682	1	0.00E+00	1223
mtDNA	NC035616.1	98.694	689	9	0	290831	291519	147019	146331	0.00E+00	1223
mtDNA	NC035616.1	98.975	683	5	2	281959	282640	187837	187156	0.00E+00	1221
mtDNA	NC035616.1	98.02	707	4	1	354820	355516	169599	170305	0.00E+00	1219
mtDNA	NC035616.1	99.401	668	3	1	123509	124175	180372	179705	0.00E+00	1210
mtDNA	NC035616.1	99.401	668	3	1	28125	28791	180372	179705	0.00E+00	1210
mtDNA	NC035616.1	98.82	678	8	0	193070	193747	258526	259203	0.00E+00	1208
mtDNA	NC035616.1	100	641	0	0	121857	122497	182177	181537	0.00E+00	1184
mtDNA	NC035616.1	100	641	0	0	26473	27113	182177	181537	0.00E+00	1184
mtDNA	NC035616.1	98.938	659	1	2	362342	363000	323906	324558	0.00E+00	1173
mtDNA	NC035616.1	98.204	668	7	3	87922	88586	265370	264705	0.00E+00	1162
mtDNA	NC035616.1	97.177	673	8	6	242736	243404	250679	250014	0.00E+00	1127
mtDNA	NC035616.1	99.038	624	3	1	262858	263481	233749	234369	0.00E+00	1116
mtDNA	NC035616.1	97.674	645	11	1	190812	191456	255945	256585	0.00E+00	1105

mtDNA	NC035616.1	96.505	658	9	8	348341	348985	163004	163660	0.00E+00	1075
mtDNA	NC035616.1	94.61	705	10	15	240119	240801	252274	251576	0.00E+00	1066
mtDNA	NC035616.1	95.118	676	22	4	216533	217197	284227	283552	0.00E+00	1055
mtDNA	NC035616.1	96.855	636	8	5	44111	44741	243507	242879	0.00E+00	1053
mtDNA	NC035616.1	94.883	684	14	5	85565	86227	320243	319560	0.00E+00	1050
mtDNA	NC035616.1	97.858	607	10	1	237857	238460	3964	4570	0.00E+00	1046
mtDNA	NC035616.1	99.13	575	1	1	61279	61849	266254	266828	0.00E+00	1031
mtDNA	NC035616.1	97.054	611	15	1	154861	155471	295482	296089	0.00E+00	1026
mtDNA	NC035616.1	97.959	588	10	1	385382	385967	307042	306455	0.00E+00	1018
mtDNA	NC035616.1	96.705	607	9	4	21244	21846	112748	113347	0.00E+00	1000
mtDNA	NC035616.1	96.705	607	9	4	116628	117230	112748	113347	0.00E+00	1000
mtDNA	NC035616.1	99.815	542	1	0	163851	164392	235427	234886	0.00E+00	996
mtDNA	NC035616.1	99.63	540	2	0	380679	381218	93760	93221	0.00E+00	987
mtDNA	NC035616.1	95.338	622	11	5	384744	385362	307694	307088	0.00E+00	972
mtDNA	NC035616.1	91.81	696	22	8	65196	65862	225170	225859	0.00E+00	937
mtDNA	NC035616.1	91.81	696	22	8	326818	327484	225170	225859	0.00E+00	937
mtDNA	NC035616.1	98.141	538	6	2	321312	321846	26942	26406	0.00E+00	935
mtDNA	NC035616.1	98.857	525	5	1	205167	205690	97251	97775	0.00E+00	935
mtDNA	NC035616.1	95.254	590	12	4	188020	188598	253722	254306	0.00E+00	920
mtDNA	NC035616.1	98.828	512	6	0	371930	372441	43003	42492	0.00E+00	913

mtDNA	NC035616.1	93.881	621	8	9	155485	156105	296137	296727	0.00E+00	909
mtDNA	NC035616.1	98.444	514	3	1	13839	14352	115378	115886	0.00E+00	900
mtDNA	NC035616.1	98.444	514	3	1	109223	109736	115378	115886	0.00E+00	900
mtDNA	NC035616.1	95.825	503	1	1	377685	378187	90052	90534	0.00E+00	795
mtDNA	NC035616.1	93.621	533	10	9	120356	120864	183440	182908	0.00E+00	774
mtDNA	NC035616.1	93.621	533	10	9	24972	25480	183440	182908	0.00E+00	774
mtDNA	NC035616.1	92.415	501	9	14	90649	91124	83020	83516	0.00E+00	688
mtDNA	NC035616.1	74.045	890	174	42	231428	232291	245021	244163	2.53E-83	311
mtDNA	NC035616.1	74.045	890	174	42	101580	102443	245021	244163	2.53E-83	311
mtDNA	NC035616.1	74.045	890	174	42	6196	7059	245021	244163	2.53E-83	311
mtDNA	NC035616.1	74.667	750	145	34	42823	43550	333948	333222	3.29E-77	291
NC035616.1	MT043351.1	95.545	2649	78	19	163739	166367	126277	128905	0.00E+00	4202
NC035616.1	MT043351.1	94.908	2494	101	12	226459	228934	316747	314262	0.00E+00	3879
NC035616.1	MT043351.1	97.639	2245	47	3	118011	120250	12130	14373	0.00E+00	3847
NC035616.1	MT043351.1	94.342	2386	119	8	230451	232832	90140	92513	0.00E+00	3644
NC035616.1	MT043351.1	97.532	1945	33	8	244042	245979	45134	47070	0.00E+00	3312
NC035616.1	MT043351.1	97.011	1673	50	0	126534	128206	1	1673	0.00E+00	2813
NC035616.1	MT043351.1	98.035	1578	23	4	260479	262049	272989	271413	0.00E+00	2736
NC035616.1	MT043351.1	96.105	1669	53	10	304193	305855	202504	200842	0.00E+00	2712
NC035616.1	MT043351.1	94.89	1722	65	11	302111	303826	205205	203501	0.00E+00	2671

NC035616.1	MT043351.1	93.388	1815	73	18	235608	237400	303302	305091	0.00E+00	2643
NC035616.1	MT043351.1	95.331	1649	60	10	290572	292209	21958	20316	0.00E+00	2603
NC035616.1	MT043351.1	96.487	1537	17	16	262100	263636	271420	269921	0.00E+00	2505
NC035616.1	MT043351.1	93.794	1547	95	1	148166	149711	224179	225725	0.00E+00	2324
NC035616.1	MT043351.1	95.112	1473	59	5	88538	89999	182361	183831	0.00E+00	2309
NC035616.1	MT043351.1	95.284	1357	48	5	256837	258193	152604	151264	0.00E+00	2137
NC035616.1	MT043351.1	95.557	1328	44	6	292258	293582	20304	18989	0.00E+00	2111
NC035616.1	MT043351.1	97.284	1215	30	2	134166	135377	135695	134481	0.00E+00	2058
NC035616.1	MT043351.1	94.765	1318	48	11	131365	132666	137973	136661	0.00E+00	2032
NC035616.1	MT043351.1	94.419	1326	36	10	95016	96313	5832	4517	0.00E+00	2004
NC035616.1	MT043351.1	96.222	1191	35	3	21038	22228	187131	185951	0.00E+00	1941
NC035616.1	MT043351.1	90.244	1517	78	28	298308	299786	54638	56122	0.00E+00	1917
NC035616.1	MT043351.1	83.072	2044	292	27	176452	178474	278190	280200	0.00E+00	1808
NC035616.1	MT043351.1	93.117	1235	63	12	187941	189158	80577	79348	0.00E+00	1790
NC035616.1	MT043351.1	96.532	1067	24	4	332408	333464	133152	134215	0.00E+00	1753
NC035616.1	MT043351.1	95.93	1081	34	6	116879	117953	11032	12108	0.00E+00	1744
NC035616.1	MT043351.1	93.322	1168	59	7	24019	25184	141758	142908	0.00E+00	1707
NC035616.1	MT043351.1	91.587	1248	64	21	9722	10946	346585	345356	0.00E+00	1685
NC035616.1	MT043351.1	94.649	1084	38	7	58236	59302	355561	354481	0.00E+00	1663
NC035616.1	MT043351.1	92.928	1103	52	12	289398	290491	23087	22002	0.00E+00	1581

NC035616.1	MT043351.1	95.603	978	42	1	67751	68728	334225	335201	0.00E+00	1567
NC035616.1	MT043351.1	97.143	875	25	0	166886	167760	129046	129920	0.00E+00	1478
NC035616.1	MT043351.1	96.317	896	23	6	167810	168700	129948	130838	0.00E+00	1463
NC035616.1	MT043351.1	99.366	789	4	1	244042	244830	113831	113044	0.00E+00	1428
NC035616.1	MT043351.1	95.084	895	42	2	210807	211701	109416	108524	0.00E+00	1408
NC035616.1	MT043351.1	92.262	1008	38	19	65166	66143	332222	333219	0.00E+00	1393
NC035616.1	MT043351.1	96.074	815	27	1	125023	125832	376484	377298	0.00E+00	1323
NC035616.1	MT043351.1	95.896	804	29	1	190531	191334	297789	296990	0.00E+00	1299
NC035616.1	MT043351.1	95.729	796	29	3	29737	30528	111547	112341	0.00E+00	1277
NC035616.1	MT043351.1	92.057	919	46	10	248111	249017	300075	299172	0.00E+00	1267
NC035616.1	MT043351.1	95.812	788	21	5	285735	286520	28485	27708	0.00E+00	1262
NC035616.1	MT043351.1	94.913	806	34	5	30658	31462	163797	164596	0.00E+00	1254
NC035616.1	MT043351.1	95.408	784	35	1	128505	129287	110379	109596	0.00E+00	1247
NC035616.1	MT043351.1	93.132	859	45	10	252326	253174	373613	372759	0.00E+00	1247
NC035616.1	MT043351.1	95.437	789	23	9	180432	181217	196839	197617	0.00E+00	1245
NC035616.1	MT043351.1	94.061	825	35	3	2152	2975	53482	54293	0.00E+00	1240
NC035616.1	MT043351.1	94.022	803	35	10	287265	288065	26758	25967	0.00E+00	1205
NC035616.1	MT043351.1	94.465	795	23	4	232916	233703	92525	93305	0.00E+00	1205
NC035616.1	MT043351.1	95.635	756	21	4	66868	67617	333393	334142	0.00E+00	1203
NC035616.1	MT043351.1	95.867	750	16	6	288109	288850	25968	25226	0.00E+00	1199

NC035616.1	MT043351.1	95.491	754	22	3	254384	255133	15645	16390	0.00E+00	1194
NC035616.1	MT043351.1	92.169	830	49	9	3977	4798	100581	99760	0.00E+00	1158
NC035616.1	MT043351.1	96.491	684	20	4	187156	187837	81379	80698	0.00E+00	1127
NC035616.1	MT043351.1	94.061	724	30	4	1277	1992	163526	162808	0.00E+00	1086
NC035616.1	MT043351.1	90.233	860	42	18	331530	332369	132306	133143	0.00E+00	1085
NC035616.1	MT043351.1	84.036	1234	74	59	295536	296717	244363	245525	0.00E+00	1074
NC035616.1	MT043351.1	96.729	642	20	1	181537	182177	197825	198466	0.00E+00	1068
NC035616.1	MT043351.1	95.489	665	27	3	25591	26254	143072	143734	0.00E+00	1059
NC035616.1	MT043351.1	94.838	678	20	6	179705	180372	196156	196828	0.00E+00	1044
NC035616.1	MT043351.1	91.867	750	31	10	96359	97089	4504	3766	0.00E+00	1020
NC035616.1	MT043351.1	95.447	593	24	1	297440	298029	319329	318737	0.00E+00	942
NC035616.1	MT043351.1	94.754	610	24	4	146417	147019	335642	335034	0.00E+00	942
NC035616.1	MT043351.1	89.345	779	39	17	90892	91659	184514	185259	0.00E+00	939
NC035616.1	MT043351.1	90.155	711	37	11	31599	32301	320361	321046	0.00E+00	894
NC035616.1	MT043351.1	97.143	525	15	0	233749	234273	93310	93834	0.00E+00	887
NC035616.1	MT043351.1	95.404	544	13	6	306455	306994	200148	199613	0.00E+00	856
NC035616.1	MT043351.1	96.024	503	15	2	57271	57768	356601	356099	0.00E+00	813
NC035616.1	MT043351.1	93.285	551	36	1	41688	42237	274762	275312	0.00E+00	811
NC035616.1	MT043351.1	88.739	666	46	14	163006	163660	125586	126233	0.00E+00	787
NC035616.1	MT043351.1	92.939	524	36	1	258589	259111	150922	150399	0.00E+00	761

NC035616.1	MT043351.1	91.86	516	26	8	324049	324558	67886	68391	0.00E+00	706
NC035616.1	MT043351.1	87.931	580	30	18	125956	126533	377323	377864	0.00E+00	647
NC035616.1	MT043351.1	74.7	751	143	37	333222	333948	45255	45982	3.08E-77	291
NC035616.1	MT043351.1	73.256	688	142	37	333222	333888	113710	113044	6.85E-54	213
MT043351.1	NC040010.1	95.582	4776	151	22	196043	200772	339030	343791	0.00E+00	7596
MT043351.1	NC040010.1	94.402	4627	172	30	18640	23205	144619	140019	0.00E+00	7029
MT043351.1	NC040010.1	93.879	4460	161	38	200798	205209	343787	348182	0.00E+00	6621
MT043351.1	NC040010.1	96.108	3957	119	9	29229	33182	23909	19985	0.00E+00	6421
MT043351.1	NC040010.1	95.334	4029	145	13	89882	93876	457539	461558	0.00E+00	6359
MT043351.1	NC040010.1	97.323	3511	82	9	11018	14522	47212	50716	0.00E+00	5952
MT043351.1	NC040010.1	95.787	3560	77	24	269929	273430	200080	203624	0.00E+00	5675
MT043351.1	NC040010.1	95.412	3531	113	21	125561	129060	288728	285216	0.00E+00	5578
MT043351.1	NC040010.1	95.318	3353	121	12	18640	21960	155957	152609	0.00E+00	5289
MT043351.1	NC040010.1	94.182	3489	123	30	205274	208717	348520	351973	0.00E+00	5245
MT043351.1	NC040010.1	96.174	2509	80	12	314262	316759	232342	234845	0.00E+00	4087
MT043351.1	NC040010.1	94.302	2580	100	15	3731	6265	257671	255094	0.00E+00	3906
MT043351.1	NC040010.1	92.046	2703	125	39	298792	301422	226836	229520	0.00E+00	3718
MT043351.1	NC040010.1	95.816	2175	69	9	135799	137961	211999	209835	0.00E+00	3493
MT043351.1	NC040010.1	96.457	2117	73	1	303302	305416	454690	452574	0.00E+00	3493
MT043351.1	NC040010.1	94.688	2278	69	14	333398	335642	165731	167989	0.00E+00	3489

MT043351.1	NC040010.1	95.339	2167	84	12	25084	27239	137797	135637	0.00E+00	3426
MT043351.1	NC040010.1	94.986	2114	73	14	354599	356691	263634	261533	0.00E+00	3286
MT043351.1	NC040010.1	96.622	1806	54	2	1	1801	110152	111955	0.00E+00	2990
MT043351.1	NC040010.1	96.077	1810	53	6	129046	130840	284945	283139	0.00E+00	2933
MT043351.1	NC040010.1	90.791	2237	104	39	67888	70053	449399	447194	0.00E+00	2896
MT043351.1	NC040010.1	90.121	2146	162	32	296474	298593	224465	226586	0.00E+00	2743
MT043351.1	NC040010.1	96.169	1540	49	4	182499	184037	291070	292600	0.00E+00	2508
MT043351.1	NC040010.1	95.208	1586	60	6	185605	187186	372824	371251	0.00E+00	2494
MT043351.1	NC040010.1	94.144	1571	88	4	224159	225725	424870	426440	0.00E+00	2388
MT043351.1	NC040010.1	95.792	1402	49	3	151265	152656	16826	15425	0.00E+00	2254
MT043351.1	NC040010.1	92.283	1568	69	13	301444	302973	229513	231066	0.00E+00	2178
MT043351.1	NC040010.1	91.683	1575	77	21	23205	24759	139991	138451	0.00E+00	2134
MT043351.1	NC040010.1	97.727	1188	27	0	134481	135668	213318	212131	0.00E+00	2045
MT043351.1	NC040010.1	92.702	1384	49	15	132274	133629	476331	477690	0.00E+00	1949
MT043351.1	NC040010.1	92.037	1419	51	9	309152	310520	299124	297718	0.00E+00	1938
MT043351.1	NC040010.1	96.872	1151	32	2	2589	3736	258928	257779	0.00E+00	1923
MT043351.1	NC040010.1	95.177	1161	36	13	141758	142908	97291	98441	0.00E+00	1816
MT043351.1	NC040010.1	92.012	1327	47	26	376484	377771	108722	110028	0.00E+00	1808
MT043351.1	NC040010.1	97.881	991	17	3	46086	47076	1589	2575	0.00E+00	1711
MT043351.1	NC040010.1	91.325	1268	65	25	79348	80598	411967	413206	0.00E+00	1690

MT043351.1	NC040010.1	92.908	1128	38	13	318415	319518	396710	395601	0.00E+00	1602
MT043351.1	NC040010.1	92.824	1087	48	13	55077	56145	72841	71767	0.00E+00	1548
MT043351.1	NC040010.1	91.904	1087	56	16	99766	100833	188728	189801	0.00E+00	1491
MT043351.1	NC040010.1	92.823	1045	46	15	332304	333342	164146	165167	0.00E+00	1487
MT043351.1	NC040010.1	95.861	894	35	2	108523	109416	409565	410456	0.00E+00	1445
MT043351.1	NC040010.1	96.471	850	22	2	163755	164596	303504	304353	0.00E+00	1397
MT043351.1	NC040010.1	95.039	887	35	4	109596	110480	362171	363050	0.00E+00	1386
MT043351.1	NC040010.1	96.014	828	23	3	53493	54310	132506	133333	0.00E+00	1338
MT043351.1	NC040010.1	90.707	990	75	12	45134	46111	122067	123051	0.00E+00	1303
MT043351.1	NC040010.1	96.094	768	27	1	33882	34649	178827	179591	0.00E+00	1249
MT043351.1	NC040010.1	98.692	688	9	0	133528	134215	477690	478377	0.00E+00	1221
MT043351.1	NC040010.1	91.135	925	45	13	320100	321007	77270	78174	0.00E+00	1219
MT043351.1	NC040010.1	94.332	794	38	6	113044	113831	122859	122067	0.00E+00	1210
MT043351.1	NC040010.1	95.745	752	21	4	80682	81426	413246	413993	0.00E+00	1201
MT043351.1	NC040010.1	94.226	762	23	8	307944	308689	300425	299669	0.00E+00	1144
MT043351.1	NC040010.1	96.246	666	22	3	143072	143735	99019	99683	0.00E+00	1088
MT043351.1	NC040010.1	94.452	703	23	4	27691	28386	134970	134277	0.00E+00	1068
MT043351.1	NC040010.1	94.548	697	28	4	353691	354382	265374	264683	0.00E+00	1068
MT043351.1	NC040010.1	92.848	741	43	3	15660	16390	112604	113344	0.00E+00	1066
MT043351.1	NC040010.1	96.463	622	21	1	1940	2561	259535	258915	0.00E+00	1026

MT043351.1	NC040010.1	93.722	669	32	5	374309	374972	326206	326869	0.00E+00	994
MT043351.1	NC040010.1	93.445	656	43	0	274739	275394	408832	408177	0.00E+00	974
MT043351.1	NC040010.1	96.128	594	15	4	124908	125493	289499	288906	0.00E+00	963
MT043351.1	NC040010.1	91.728	677	38	5	66345	67008	451117	450446	0.00E+00	924
MT043351.1	NC040010.1	94.407	590	21	5	209136	209713	352622	353211	0.00E+00	896
MT043351.1	NC040010.1	91.592	666	31	8	187584	188232	370893	370236	0.00E+00	896
MT043351.1	NC040010.1	87.5	800	51	17	184500	185270	292673	293452	0.00E+00	878
MT043351.1	NC040010.1	95.183	519	20	1	162895	163413	61588	62101	0.00E+00	815
MT043351.1	NC040010.1	91.05	581	51	1	234023	234603	52630	52051	0.00E+00	784
MT043351.1	NC040010.1	85.942	754	73	18	71845	72577	218337	217596	0.00E+00	774
MT043351.1	NC040010.1	89.661	619	40	9	346483	347080	178824	178209	0.00E+00	767
MT043351.1	NC040010.1	87.106	667	49	18	17933	18587	157004	156363	0.00E+00	721
MT043351.1	NC040010.1	92.829	502	18	7	67007	67490	450375	449874	0.00E+00	712
MT043351.1	NC040010.1	89.981	539	23	10	269405	269917	199490	200023	0.00E+00	667
MT043351.1	NC040010.1	73.333	570	124	26	45255	45810	478130	478685	2.14E-45	185
MT043351.1	NC040010.1	73.333	570	124	26	113155	113710	478685	478130	2.14E-45	185

Table S9. RNA editing events predicted in *Punica granatum* mitochondrial genome using BEDTools software

Region	Position	Reference	Strand	Coverage-q30	MeanQ	BaseCount[A,C,G,T]	AllSubs	Frequency
atp1	1490	C	2	12161	37.48	[15, 219, 12, 11915]	CT CA CG	0.98
atp1	1523	C	2	12587	37.09	[1, 229, 0, 12357]	CT CA	0.98
atp4	56	C	2	7445	37.79	[0, 223, 0, 7222]	CT	0.97
atp4	59	C	2	7169	37.61	[1, 397, 0, 6771]	CT CA	0.94
atp4	89	C	2	8095	37.64	[1, 240, 0, 7854]	CT CA	0.97
atp4	118	C	2	8597	37.6	[1, 176, 0, 8420]	CT CA	0.98
atp4	138	C	2	8901	37.66	[1, 634, 0, 8266]	CT CA	0.93
atp4	171	C	2	11153	37.47	[3, 8051, 2, 3097]	CT CA CG	0.28
atp4	215	C	2	13220	37.56	[0, 315, 0, 12905]	CT	0.98
atp4	227	C	2	12789	37.33	[5, 533, 2, 12249]	CT CA CG	0.96
atp4	248	C	2	13277	37.6	[3, 443, 0, 12831]	CT CA	0.97
atp4	251	C	2	13571	37.71	[0, 487, 2, 13082]	CT CG	0.96
atp4	395	C	2	14907	37.53	[3, 329, 1, 14574]	CT CA CG	0.98
atp4	407	C	2	14507	37.72	[1, 489, 0, 14017]	CT CA	0.97
atp4	416	C	2	13654	37.14	[14, 268, 5, 13367]	CT CA CG	0.98
atp6	37	C	2	8332	37.59	[1, 218, 0, 8113]	CT CA	0.97
atp6	116	C	2	9566	37.65	[2, 342, 0, 9222]	CT CA	0.96
atp6	173	C	2	10024	37.62	[5, 328, 3, 9688]	CT CA CG	0.97
atp6	229	C	2	9995	37.67	[0, 200, 5, 9790]	CT CG	0.98
atp6	236	C	2	10264	37.63	[0, 491, 2, 9771]	CT CG	0.95
atp6	254	C	2	9358	37.74	[0, 456, 0, 8902]	CT	0.95
atp6	262	C	2	9245	37.72	[0, 209, 0, 9036]	CT	0.98
atp6	269	C	2	8857	37.44	[4, 272, 1, 8580]	CT CA CG	0.97
atp6	270	C	2	8450	36.98	[0, 4034, 1, 4415]	CT CG	0.52
atp6	401	C	2	9961	37.68	[1, 166, 0, 9794]	CT CA	0.98
atp6	460	C	2	10010	37.56	[0, 145, 0, 9865]	CT	0.99
atp6	463	C	2	10043	37.71	[1, 172, 0, 9870]	CT CA	0.98
atp6	485	C	2	9087	37.63	[3, 379, 0, 8705]	CT CA	0.96
atp6	527	C	2	9044	37.66	[0, 224, 0, 8820]	CT	0.98
atp6	548	C	2	7768	37.17	[5, 556, 2, 7205]	CT CA CG	0.93
atp6	549	C	2	7359	37.09	[0, 6250, 0, 1109]	CT	0.15
atp6	664	C	2	4090	37.47	[1, 133, 1, 3955]	CT CA CG	0.97

atp6	671	C	2	3916	37.23	[0, 155, 0, 3761]	CT	0.96
atp6	680	C	2	3391	37.65	[4, 130, 0, 3257]	CT CA	0.96
atp6	707	C	2	2125	37.59	[5, 162, 4, 1954]	CT CA CG	0.92
atp6	718	C	2	1832	37.71	[4, 130, 1, 1697]	CT CA CG	0.93
atp8	30	C	2	3347	37.47	[1, 2806, 0, 540]	CT CA	0.16
atp8	47	C	2	3555	37.63	[7, 879, 0, 2669]	CT CA	0.75
atp8	58	C	2	3752	37.62	[2, 1166, 1, 2583]	CT CA CG	0.69
atp8	76	C	2	3983	37.67	[2, 237, 0, 3744]	CT CA	0.94
atp8	77	C	2	3983	37.61	[1, 395, 0, 3587]	CT CA	0.9
atp8	452	C	2	13213	37.51	[1, 434, 0, 12778]	CT CA	0.97
atp9	20	C	2	80921	37.63	[23, 751, 0, 80147]	CT CA	0.99
atp9	50	C	2	96704	37.64	[26, 761, 0, 95917]	CT CA	0.99
atp9	134	C	2	147385	37.68	[88, 1885, 0, 145412]	CT CA	0.99
atp9	190	C	2	157287	37.18	[49, 137552, 13, 19673]	CT CA CG	0.13
atp9	191	C	2	163144	37.72	[247, 6450, 92, 156355]	CT CA CG	0.96
atp9	223	C	2	171282	37.65	[12, 1890, 6, 169374]	CT CA CG	0.99
ccmB	28	C	2	13441	37.32	[0, 11622, 0, 1819]	CT	0.14
ccmB	39	C	2	13728	37.48	[3, 2651, 0, 11074]	CT CA	0.81
ccmB	43	C	2	13101	37.16	[3, 10073, 0, 3025]	CT CA	0.23
ccmB	48	C	2	13241	37.55	[0, 2780, 1, 10460]	CT CG	0.79
ccmB	50	C	2	13321	37.58	[3, 2741, 0, 10577]	CT CA	0.79
ccmB	71	C	2	14947	37.44	[0, 12404, 1, 2542]	CT CG	0.17
ccmB	90	C	2	18492	37.62	[1, 1780, 0, 16711]	CT CA	0.9
ccmB	128	C	2	20218	37.36	[0, 17645, 0, 2573]	CT	0.13
ccmB	148	C	2	1985	37.11	[1, 409, 0, 1575]	CT CA	0.79
ccmB	149	C	2	2035	37.46	[0, 459, 0, 1576]	CT	0.77
ccmB	160	C	2	2181	37.11	[0, 457, 0, 1724]	CT	0.79
ccmB	164	C	2	2245	37.51	[2, 515, 1, 1727]	CT CA CG	0.77
ccmB	172	C	2	2255	37.22	[0, 285, 0, 1970]	CT	0.87
ccmB	179	C	2	2169	37.31	[0, 494, 0, 1675]	CT	0.77
ccmB	181	C	2	2148	37.27	[1, 514, 0, 1633]	CT CA	0.76
ccmB	193	C	2	1861	37.46	[0, 254, 0, 1607]	CT	0.86

ccmB	194	C	2	1896	37.52	[0, 253, 0, 1643]	CT	0.87
ccmB	286	C	2	2837	37.52	[0, 609, 0, 2228]	CT	0.79
ccmB	304	C	2	2984	37.49	[1, 334, 0, 2649]	CT CA	0.89
ccmB	313	C	2	3028	37.59	[1, 477, 2, 2548]	CT CG CA	0.84
ccmB	357	C	2	3783	37.14	[0, 3129, 0, 654]	CT	0.17
ccmB	367	C	2	3989	37.48	[0, 485, 0, 3504]	CT	0.88
ccmB	375	C	2	4167	37.28	[0, 2885, 0, 1282]	CT	0.31
ccmB	379	C	2	4190	37.32	[2, 1204, 0, 2984]	CT CA	0.71
ccmB	391	C	2	3901	37	[4, 1558, 0, 2339]	CT CA	0.6
ccmB	392	C	2	4011	37.34	[0, 1376, 2, 2633]	CT CG	0.66
ccmB	406	C	2	4109	37.4	[0, 1013, 0, 3096]	CT	0.75
ccmB	424	C	2	4203	37.59	[0, 606, 0, 3597]	CT	0.86
ccmB	428	C	2	4202	37.5	[0, 281, 0, 3921]	CT	0.93
ccmB	467	C	2	3748	37.46	[1, 475, 2, 3270]	CT CG CA	0.87
ccmB	475	C	2	3424	37.41	[0, 321, 0, 3103]	CT	0.91
ccmB	476	C	2	3465	37.62	[0, 345, 0, 3120]	CT	0.9
ccmB	485	C	2	3482	37.64	[1, 377, 0, 3104]	CT CA	0.89
ccmB	494	C	2	3671	37.66	[0, 442, 0, 3229]	CT	0.88
ccmB	502	C	2	2993	37.46	[36, 353, 16, 2588]	CT CA CG	0.88
ccmB	503	C	2	2842	37.61	[8, 412, 1, 2421]	CT CA CG	0.85
ccmB	512	C	2	2774	37.52	[1, 360, 0, 2413]	CT CA	0.87
ccmB	514	C	2	2769	37.69	[0, 316, 0, 2453]	CT	0.89
ccmB	547	C	2	2652	36.51	[0, 2335, 0, 317]	CT	0.12
ccmB	551	C	2	2956	37.63	[2, 211, 0, 2743]	CT CA	0.93
ccmB	554	C	2	3358	37.59	[0, 317, 0, 3041]	CT	0.91
ccmB	566	C	2	3040	37.14	[0, 291, 0, 2749]	CT	0.9
ccmB	569	C	2	3067	37.39	[0, 306, 0, 2761]	CT	0.9
ccmB	572	C	2	3079	37.6	[0, 219, 0, 2860]	CT	0.93
ccmB	576	C	2	3059	37.24	[0, 807, 0, 2252]	CT	0.74
ccmB	596	C	2	3046	37.35	[0, 331, 0, 2715]	CT	0.89
ccmB	611	C	2	2794	37.02	[0, 269, 0, 2525]	CT	0.9
ccmC	76	C	2	2072	37.65	[2, 121, 0, 1949]	CT CA	0.94
ccmC	103	C	2	2288	37.63	[0, 407, 0, 1881]	CT	0.82
ccmC	115	C	2	2485	37.61	[1, 198, 1, 2285]	CT CA CG	0.92

ccmC	161	C	2	2850	37.58	[2, 411, 0, 2437]	CT CA	0.86
ccmC	179	C	2	2838	37.53	[5, 91, 0, 2742]	CT CA	0.97
ccmC	184	C	2	2791	37.52	[0, 142, 1, 2648]	CT CG	0.95
ccmC	331	C	2	3350	37.45	[6, 329, 4, 3011]	CT CA CG	0.9
ccmC	395	C	2	5199	37.52	[0, 1497, 0, 3702]	CT	0.71
ccmC	399	C	2	5338	37.36	[0, 1781, 0, 3557]	CT	0.67
ccmC	436	C	2	6624	37.6	[1, 79, 0, 6544]	CT CA	0.99
ccmC	446	C	2	6809	37.69	[3, 404, 2, 6400]	CT CA CG	0.94
ccmC	458	C	2	7089	37.73	[1, 351, 0, 6737]	CT CA	0.95
ccmC	463	C	2	7284	37.74	[3, 596, 1, 6684]	CT CA CG	0.92
ccmC	473	C	2	7517	37.69	[0, 448, 2, 7067]	CT CG	0.94
ccmC	497	C	2	7410	37.44	[3, 271, 0, 7136]	CT CA	0.96
ccmC	521	C	2	6534	37.67	[0, 300, 0, 6234]	CT	0.95
ccmC	548	C	2	5975	37.39	[7, 305, 1, 5662]	CT CA CG	0.95
ccmC	568	C	2	6175	37.25	[1, 160, 0, 6014]	CT CA	0.97
ccmC	575	C	2	6137	37.2	[5, 349, 0, 5783]	CT CA	0.94
ccmC	605	C	2	5141	37.11	[0, 458, 2, 4681]	CT CG	0.91
ccmC	608	C	2	5184	37.38	[5, 54, 0, 5125]	CT CA	0.99
ccmC	614	C	2	5000	37.75	[0, 115, 0, 4885]	CT	0.98
ccmC	618	C	2	4513	36.83	[0, 3869, 1, 643]	CT CG	0.14
ccmC	619	C	2	4782	37.68	[3, 92, 1, 4686]	CT CA CG	0.98
ccmC	624	C	2	4275	37.05	[0, 2122, 0, 2153]	CT	0.5
ccmC	650	C	2	3175	37.5	[0, 255, 1, 2919]	CT CG	0.92
ccmC	665	C	2	2904	37.21	[0, 55, 0, 2849]	CT	0.98
ccmFC	38	C	2	716	37.54	[0, 66, 0, 650]	CT	0.91
ccmFC	39	C	2	717	37.42	[0, 490, 0, 227]	CT	0.32
ccmFC	50	C	2	743	37.39	[0, 95, 0, 648]	CT	0.87
ccmFC	52	C	2	749	37.59	[0, 100, 0, 649]	CT	0.87
ccmFC	103	C	2	815	37.42	[0, 62, 0, 753]	CT	0.92
ccmFC	119	C	2	840	37.52	[1, 93, 0, 746]	CT CA	0.89
ccmFC	122	C	2	838	37.53	[0, 77, 0, 761]	CT	0.91
ccmFC	146	C	2	836	37.62	[0, 64, 0, 772]	CT	0.92
ccmFC	151	C	2	822	37.55	[1, 27, 0, 794]	CT CA	0.97
ccmFC	155	C	2	901	37.58	[0, 282, 0, 619]	CT	0.69

ccmFC	160	C	2	914	37.53	[1, 59, 0, 854]	CT CA	0.94
ccmFC	192	C	2	1003	37.3	[0, 896, 0, 107]	CT	0.11
ccmFC	305	C	2	1049	37.35	[3, 331, 0, 715]	CT CA	0.68
ccmFC	310	C	2	1140	37.63	[0, 143, 0, 997]	CT	0.87
ccmFC	391	C	2	1191	37.52	[1, 82, 0, 1108]	CT CA	0.93
ccmFC	406	C	2	1279	37.72	[0, 78, 0, 1201]	CT	0.94
ccmFC	412	C	2	1251	37.3	[0, 301, 0, 950]	CT	0.76
ccmFC	706	C	2	1152	36.91	[0, 200, 0, 952]	CT	0.83
ccmFC	878	C	2	1877	37.59	[2, 166, 0, 1709]	CT CA	0.91
ccmFC	1142	C	2	1670	37.72	[0, 55, 0, 1615]	CT	0.97
ccmFC	1163	C	2	1455	37.67	[0, 85, 0, 1370]	CT	0.94
ccmFC	1237	C	2	1408	37.69	[1, 25, 0, 1382]	CT CA	0.98
ccmFC	1271	C	2	983	37.06	[0, 324, 0, 659]	CT	0.67
ccmFC	1318	C	2	757	37.53	[0, 13, 0, 744]	CT	0.98
ccmFN	38	C	2	347	37.69	[0, 61, 0, 286]	CT	0.82
ccmFN	98	C	2	364	37.36	[0, 67, 0, 297]	CT	0.82
ccmFN	137	C	2	306	37.75	[0, 26, 0, 280]	CT	0.92
ccmFN	142	C	2	305	37.71	[0, 37, 0, 268]	CT	0.88
ccmFN	151	C	2	292	37.5	[0, 32, 0, 260]	CT	0.89
ccmFN	248	C	2	268	37.67	[0, 17, 0, 251]	CT	0.94
ccmFN	256	C	2	286	37.38	[0, 30, 0, 256]	CT	0.9
ccmFN	263	C	2	307	37.6	[0, 30, 0, 277]	CT	0.9
ccmFN	279	C	2	335	37.39	[0, 32, 0, 303]	CT	0.9
ccmFN	283	C	2	332	37.46	[0, 36, 0, 296]	CT	0.89
ccmFN	372	C	2	512	37.63	[0, 91, 0, 421]	CT	0.82
ccmFN	400	C	2	526	37.35	[1, 437, 0, 88]	CT CA	0.17
ccmFN	559	C	2	485	37.12	[0, 431, 0, 54]	CT	0.11
ccmFN	605	C	2	492	37.07	[8, 378, 0, 106]	CT CA	0.22
ccmFN	607	C	2	502	37.65	[0, 80, 0, 422]	CT	0.84
ccmFN	707	C	2	424	37.31	[1, 34, 0, 389]	CT CA	0.92
ccmFN	716	C	2	426	37.61	[0, 51, 0, 375]	CT	0.88
ccmFN	747	C	2	391	36.92	[0, 345, 0, 46]	CT	0.12
ccmFN	754	C	2	411	37.55	[0, 32, 0, 379]	CT	0.92
ccmFN	759	C	2	412	37.24	[0, 311, 0, 101]	CT	0.25

ccmFN	776	C	2	356	37.62	[0, 26, 0, 330]	CT	0.93
ccmFN	788	C	2	340	37.59	[0, 20, 0, 320]	CT	0.94
ccmFN	803	C	2	350	37.72	[0, 13, 0, 337]	CT	0.96
ccmFN	952	C	2	359	37.59	[0, 18, 0, 341]	CT	0.95
ccmFN	1187	C	2	303	37.43	[0, 29, 0, 274]	CT	0.9
ccmFN	1270	C	2	295	37.41	[0, 23, 0, 272]	CT	0.92
ccmFN	1298	C	2	291	37.16	[0, 31, 0, 260]	CT	0.89
ccmFN	1315	C	2	307	37.35	[1, 24, 0, 282]	CT CA	0.92
ccmFN	1330	C	2	320	37.17	[2, 27, 0, 291]	CT CA	0.92
ccmFN	1348	C	2	414	37.33	[0, 28, 0, 386]	CT	0.93
ccmFN	1381	C	2	444	37.61	[1, 25, 0, 418]	CT CA	0.94
ccmFN	1399	C	2	524	37.64	[0, 26, 0, 498]	CT	0.95
ccmFN	1442	C	2	484	37.66	[1, 33, 0, 450]	CT CA	0.93
ccmFN	1462	C	2	450	37.54	[0, 16, 0, 434]	CT	0.96
ccmFN	1490	C	2	442	37.41	[0, 347, 0, 95]	CT	0.21
cob	180	C	2	5690	37.21	[0, 4760, 0, 930]	CT	0.16
cob	286	C	2	6254	37.46	[1, 123, 0, 6130]	CT CA	0.98
cob	298	C	2	6551	37.44	[0, 143, 0, 6408]	CT	0.98
cob	325	C	2	6929	37.65	[0, 109, 0, 6820]	CT	0.98
cob	358	C	2	7334	37.63	[0, 97, 4, 7233]	CT CG	0.99
cob	407	C	2	8357	37.64	[1, 92, 0, 8264]	CT CA	0.99
cob	568	C	2	7129	37.66	[1, 106, 0, 7022]	CT CA	0.99
cob	853	C	2	8166	37.61	[10, 116, 2, 8038]	CT CA CG	0.99
cob	907	C	2	6485	36.93	[0, 5542, 3, 940]	CT CG	0.15
cob	908	C	2	6730	37.64	[15, 142, 3, 6570]	CT CA CG	0.98
cob	982	C	2	8367	37.56	[1, 290, 1, 8075]	CT CA CG	0.97
cob	1084	C	2	10625	37.47	[1, 58, 0, 10566]	CT CA	0.99
cob	1406	C	2	13394	37.52	[24, 3589, 3, 9778]	CT CA CG	0.73
cox1	11	C	2	9820	37.63	[1, 913, 0, 8906]	CT CA	0.91
cox1	15	C	2	9183	36.88	[0, 7106, 1, 2076]	CT CG	0.23
cox1	242	C	2	15508	37.44	[3, 457, 4, 15044]	CT CG CA	0.97
cox1	254	C	2	16255	37.41	[2, 479, 1, 15773]	CT CA CG	0.97
cox1	265	C	2	16916	37.34	[0, 8480, 0, 8436]	CT	0.5
cox1	352	C	2	20093	37.48	[4, 349, 0, 19740]	CT CA	0.98

cox1	443	C	2	23986	37.63	[3, 173, 0, 23810]	CT CA	0.99
cox1	452	C	2	23732	37.51	[1, 262, 0, 23469]	CT CA	0.99
cox1	515	C	2	23664	37.19	[4, 294, 3, 23363]	CT CA CG	0.99
cox1	551	C	2	26188	37.55	[3, 334, 0, 25851]	CT CA	0.99
cox1	575	C	2	21734	37.67	[2, 236, 1, 21495]	CT CA CG	0.99
cox1	590	C	2	22173	37.73	[7, 235, 3, 21928]	CT CA CG	0.99
cox1	668	C	2	22397	37.54	[2, 255, 1, 22139]	CT CA CG	0.99
cox1	715	C	2	20192	37.73	[1, 129, 3, 20059]	CT CG CA	0.99
cox1	746	C	2	22428	37.58	[0, 112, 2, 22314]	CT CG	1
cox1	761	C	2	21269	37.48	[7, 263, 0, 20999]	CT CA	0.99
cox1	1037	C	2	18592	36.93	[27, 151, 13, 18401]	CT CA CG	0.99
cox1	1079	C	2	20703	37.01	[8, 194, 2, 20499]	CT CA CG	0.99
cox1	1186	C	2	22813	37.49	[0, 349, 0, 22464]	CT	0.98
cox1	1274	C	2	24623	37.65	[2, 136, 2, 24483]	CT CA CG	0.99
cox1	1279	C	2	24615	37.39	[7, 92, 3, 24513]	CT CA CG	1
cox1	1402	C	2	27865	37.73	[2, 135, 2, 27726]	CT CG CA	1
cox1	1433	C	2	24626	37.71	[2, 334, 3, 24287]	CT CG CA	0.99
cox2	27	C	2	7056	37.48	[0, 2894, 0, 4162]	CT	0.59
cox2	38	C	2	7606	37.42	[0, 462, 0, 7144]	CT	0.94
cox2	71	C	2	9553	37.4	[2, 116, 0, 9435]	CT CA	0.99
cox2	161	C	2	10373	37.68	[2, 113, 0, 10258]	CT CA	0.99
cox2	163	C	2	10535	37.64	[0, 112, 0, 10423]	CT	0.99
cox2	253	C	2	13417	37.68	[3, 184, 2, 13228]	CT CA CG	0.99
cox2	278	C	2	14182	37.72	[9, 198, 5, 13970]	CT CA CG	0.99
cox2	379	C	2	12644	37.63	[2, 131, 1, 12510]	CT CA CG	0.99
cox2	443	C	2	13566	37.69	[2, 417, 0, 13147]	CT CA	0.97
cox2	476	C	2	14441	37.65	[0, 163, 0, 14278]	CT	0.99
cox2	544	C	2	14740	37.43	[2, 153, 0, 14585]	CT CA	0.99
cox2	557	C	2	15745	37.56	[1, 227, 1, 15516]	CT CG CA	0.99
cox2	581	C	2	14623	37.72	[1, 329, 1, 14292]	CT CA CG	0.98
cox2	632	C	2	13618	37.74	[0, 96, 1, 13521]	CT CG	0.99
cox2	698	C	2	11443	37.61	[4, 75, 4, 11360]	CT CA CG	0.99
cox2	721	C	2	9510	37.09	[0, 112, 0, 9398]	CT	0.99
cox2	742	C	2	8889	37.61	[1, 31, 0, 8857]	CT CA	1

cox3	112	C	2	10676	37.44	[6, 216, 4, 10450]	CT CA CG	0.98
cox3	270	C	2	8382	36.74	[1, 7003, 0, 1378]	CT CA	0.16
cox3	304	C	2	8469	37.52	[2, 202, 4, 8261]	CT CG CA	0.98
cox3	311	C	2	8316	37.16	[4, 158, 1, 8153]	CT CA CG	0.98
cox3	413	C	2	9508	37.41	[39, 156, 21, 9292]	CT CA CG	0.98
cox3	512	C	2	13656	37.68	[1, 317, 0, 13338]	CT CA	0.98
cox3	565	C	2	13581	37.6	[43, 148, 17, 13373]	CT CA CG	0.99
cox3	566	C	2	13344	37.58	[10, 129, 4, 13201]	CT CA CG	0.99
cox3	653	C	2	12641	37.78	[1, 55, 0, 12585]	CT CA	1
cox3	754	C	2	3163	37.44	[0, 35, 0, 3128]	CT	0.99
matR	32	C	2	1502	37.3	[0, 339, 0, 1163]	CT	0.77
matR	43	C	2	1665	37.08	[0, 412, 0, 1253]	CT	0.75
matR	147	C	2	2009	37.19	[0, 1668, 0, 341]	CT	0.17
matR	193	C	2	1960	37.26	[1, 386, 1, 1572]	CT CA CG	0.8
matR	413	C	2	1944	37.49	[0, 474, 0, 1470]	CT	0.76
matR	923	C	2	2484	37.62	[1, 257, 1, 2225]	CT CA CG	0.9
matR	1545	C	2	3168	37.34	[0, 2459, 1, 708]	CT CG	0.22
matR	1679	C	2	4487	36.92	[0, 1328, 0, 3159]	CT	0.7
matR	1700	C	2	4855	37.39	[0, 712, 0, 4143]	CT	0.85
matR	1734	C	2	4427	37.32	[2, 357, 0, 4068]	CT CA	0.92
matR	1756	C	2	4419	37.48	[4, 891, 3, 3521]	CT CA CG	0.8
matR	1844	C	2	3013	37.64	[2, 147, 0, 2864]	CT CA	0.95
matR	1875	C	2	2858	36.77	[0, 2503, 1, 354]	CT CG	0.12
mttB	27	C	2	4698	37.05	[0, 4139, 0, 559]	CT	0.12
mttB	37	C	2	5204	37.48	[0, 1688, 0, 3516]	CT	0.68
mttB	84	C	2	5903	37.16	[0, 1332, 0, 4571]	CT	0.77
mttB	85	C	2	5834	37.05	[0, 766, 0, 5068]	CT	0.87
mttB	101	C	2	6012	37.4	[0, 1954, 0, 4058]	CT	0.67
mttB	104	C	2	6051	37.63	[1, 1650, 0, 4400]	CT CA	0.73
mttB	161	C	2	4849	37.62	[0, 743, 1, 4105]	CT CG	0.85
mttB	175	C	2	4316	36.96	[1, 1521, 0, 2794]	CT CA	0.65
mttB	235	C	2	2238	37.47	[1, 687, 0, 1550]	CT CA	0.69
mttB	304	C	2	2432	37.68	[0, 237, 0, 2195]	CT	0.9
mttB	317	C	2	2337	37.04	[0, 516, 0, 1821]	CT	0.78

mttB	346	C	2	1967	37.08	[0, 713, 0, 1254]	CT	0.64
mttB	347	C	2	1979	37.23	[0, 440, 0, 1539]	CT	0.78
mttB	349	C	2	2019	37.38	[1, 111, 0, 1907]	CT CA	0.94
mttB	352	C	2	2058	37.35	[0, 936, 0, 1122]	CT	0.55
mttB	380	C	2	2331	37.67	[0, 262, 0, 2069]	CT	0.89
mttB	410	C	2	2099	37.54	[2, 162, 0, 1935]	CT CA	0.92
mttB	470	C	2	2328	37.58	[0, 340, 0, 1988]	CT	0.85
mttB	478	C	2	2292	37.13	[0, 749, 0, 1543]	CT	0.67
mttB	521	C	2	2271	37.41	[0, 900, 0, 1371]	CT	0.6
mttB	527	C	2	2341	37.43	[0, 269, 1, 2071]	CT CG	0.89
mttB	583	C	2	3163	37.28	[2, 328, 0, 2833]	CT CA	0.9
mttB	640	C	2	3489	37.48	[1, 981, 0, 2507]	CT CA	0.72
mttB	645	C	2	3250	36.86	[0, 2940, 0, 310]	CT	0.1
mttB	681	C	2	3410	37.09	[0, 2275, 0, 1135]	CT	0.33
mttB	686	C	2	3479	37.34	[0, 1447, 1, 2031]	CT CG	0.58
nad1	2	C	2	10353	37.37	[2, 4644, 0, 5707]	CT CA	0.55
nad1	167	C	2	9179	37.59	[0, 104, 0, 9075]	CT	0.99
nad1	215	C	2	12030	37.5	[0, 153, 0, 11877]	CT	0.99
nad1	265	C	2	11271	37.4	[5, 1055, 3, 10208]	CT CA CG	0.91
nad1	307	C	2	11309	37.31	[1, 561, 1, 10746]	CT CG CA	0.95
nad1	308	C	2	11489	37.57	[0, 723, 0, 10766]	CT	0.94
nad1	312	C	2	11549	37.22	[1, 10207, 1, 1340]	CT CG CA	0.12
nad1	336	C	2	9382	37.42	[1, 4575, 0, 4806]	CT CA	0.51
nad1	368	C	2	9148	37.6	[0, 262, 0, 8886]	CT	0.97
nad1	376	C	2	9096	37.45	[12, 309, 7, 8768]	CT CA CG	0.97
nad1	490	C	2	6263	37.15	[2, 109, 2, 6150]	CT CA CG	0.98
nad1	492	C	2	6226	37.4	[1, 387, 1, 5837]	CT CG CA	0.94
nad1	493	C	2	6425	37.66	[0, 177, 3, 6245]	CT CG	0.97
nad1	500	C	2	7109	37.67	[0, 231, 0, 6878]	CT	0.97
nad1	536	C	2	7923	37.13	[1, 163, 0, 7759]	CT CA	0.98
nad1	573	C	2	8416	37.71	[1, 205, 0, 8210]	CT CA	0.98
nad1	580	C	2	8563	37.71	[1, 129, 0, 8433]	CT CA	0.98
nad1	635	C	2	9144	37.63	[2, 207, 0, 8935]	CT CA	0.98
nad1	674	C	2	8838	37.08	[6, 367, 2, 8463]	CT CA CG	0.96

nad1	734	C	2	13305	37.63	[1, 97, 4, 13203]	CT CG CA	0.99
nad1	740	C	2	12748	37.57	[1, 140, 0, 12607]	CT CA	0.99
nad1	743	C	2	14243	37.72	[1, 139, 0, 14103]	CT CA	0.99
nad1	755	C	2	15368	37.26	[3, 174, 4, 15187]	CT CG CA	0.99
nad1	823	C	2	11695	37.44	[0, 253, 0, 11442]	CT	0.98
nad1	829	C	2	11285	37.18	[0, 9808, 0, 1477]	CT	0.13
nad1	898	C	2	6648	37.23	[12, 97, 8, 6531]	CT CA CG	0.99
nad1	909	C	2	6192	36.97	[0, 3913, 3, 2276]	CT CG	0.37
nad1	928	C	2	5582	37.43	[4, 171, 12, 5395]	CT CG CA	0.97
nad1	937	C	2	5106	36.95	[5, 54, 2, 5045]	CT CA CG	0.99
nad2	26	C	2	4687	37.47	[1, 293, 0, 4393]	CT CA	0.94
nad2	223	C	2	4280	37.47	[0, 64, 0, 4216]	CT	0.99
nad2	303	C	2	2595	36.94	[0, 2305, 0, 290]	CT	0.11
nad2	356	C	2	3285	37.64	[0, 126, 0, 3159]	CT	0.96
nad2	361	C	2	3232	37.42	[1, 153, 0, 3078]	CT CA	0.95
nad2	367	C	2	3275	37.55	[2, 66, 2, 3205]	CT CG CA	0.98
nad2	394	C	2	2965	37.55	[9, 73, 1, 2882]	CT CA CG	0.98
nad2	401	C	2	3077	37.57	[0, 192, 0, 2885]	CT	0.94
nad2	428	C	2	3300	37.31	[0, 75, 2, 3223]	CT CG	0.98
nad2	497	C	2	3305	37.53	[0, 63, 2, 3240]	CT CG	0.98
nad2	523	C	2	4315	37.49	[0, 220, 0, 4095]	CT	0.95
nad2	609	C	2	7142	37.61	[1, 706, 1, 6434]	CT CG CA	0.9
nad2	788	C	2	5969	37.49	[2, 31, 0, 5936]	CT CA	0.99
nad2	800	C	2	6094	37.69	[0, 43, 0, 6051]	CT	0.99
nad2	809	C	2	5965	37.54	[1, 37, 0, 5927]	CT CA	0.99
nad2	920	C	2	5107	37.48	[1, 70, 0, 5036]	CT CA	0.99
nad2	928	C	2	5317	37.68	[0, 92, 0, 5225]	CT	0.98
nad2	958	C	2	5746	37.7	[0, 38, 0, 5708]	CT	0.99
nad2	962	C	2	5618	37.34	[1, 346, 1, 5270]	CT CG CA	0.94
nad2	1025	C	2	5437	37.12	[0, 4849, 0, 588]	CT	0.11
nad2	1058	C	2	5418	37.62	[1, 39, 0, 5378]	CT CA	0.99
nad2	1127	C	2	5123	37.59	[0, 248, 0, 4875]	CT	0.95
nad2	1246	C	2	5432	37.04	[1, 34, 0, 5397]	CT CA	0.99
nad2	1298	C	2	6910	37.4	[0, 23, 0, 6887]	CT	1

nad2	1400	C	2	7328	37.42	[2, 996, 0, 6330]	CT CA	0.86
nad2	1403	C	2	7206	37.24	[1, 378, 0, 6827]	CT CA	0.95
nad2	1408	C	2	7195	37.49	[0, 384, 0, 6811]	CT	0.95
nad2	1416	C	2	7154	36.97	[2, 735, 2, 6415]	CT CG CA	0.9
nad2	1457	C	2	5540	37.21	[0, 44, 0, 5496]	CT	0.99
nad3	5	C	2	2658	37.47	[1, 543, 0, 2114]	CT CA	0.8
nad3	39	C	2	2749	37.45	[0, 878, 0, 1871]	CT	0.68
nad3	44	C	2	2792	37.58	[1, 341, 0, 2450]	CT CA	0.88
nad3	80	C	2	2650	37.69	[0, 292, 0, 2358]	CT	0.89
nad3	146	C	2	2854	37.27	[0, 363, 0, 2491]	CT	0.87
nad3	208	C	2	2503	37.45	[0, 346, 2, 2155]	CT CG	0.86
nad3	209	C	2	2500	37.45	[0, 309, 1, 2190]	CT CG	0.88
nad3	215	C	2	2532	37.44	[1, 453, 0, 2078]	CT CA	0.82
nad3	247	C	2	2676	36.99	[0, 488, 0, 2188]	CT	0.82
nad3	251	C	2	2589	36.96	[0, 470, 0, 2119]	CT	0.82
nad3	266	C	2	2528	37.42	[1, 666, 0, 1861]	CT CA	0.74
nad3	275	C	2	2387	37.16	[1, 459, 0, 1927]	CT CA	0.81
nad3	317	C	2	1873	37.05	[0, 281, 0, 1592]	CT	0.85
nad3	344	C	2	1527	37.4	[0, 259, 0, 1268]	CT	0.83
nad3	349	C	2	1512	37.39	[0, 346, 0, 1166]	CT	0.77
nad4	29	C	2	3748	37.51	[0, 408, 0, 3340]	CT	0.89
nad4	74	C	2	3234	37.23	[0, 1303, 0, 1931]	CT	0.6
nad4	77	C	2	3233	37.44	[0, 193, 0, 3040]	CT	0.94
nad4	84	C	2	3157	37.43	[0, 1325, 0, 1832]	CT	0.58
nad4	107	C	2	3000	37.51	[10, 144, 6, 2840]	CT CA CG	0.95
nad4	154	C	2	2693	37.21	[0, 109, 0, 2584]	CT	0.96
nad4	164	C	2	2706	37.21	[2, 129, 0, 2575]	CT CA	0.95
nad4	166	C	2	2749	37.6	[0, 180, 0, 2569]	CT	0.93
nad4	197	C	2	1745	37.45	[0, 84, 0, 1661]	CT	0.95
nad4	261	C	2	1596	36.84	[0, 1440, 0, 156]	CT	0.1
nad4	303	C	2	1416	36.62	[0, 1089, 0, 327]	CT	0.23
nad4	362	C	2	2584	37.59	[1, 129, 0, 2454]	CT CA	0.95
nad4	368	C	2	2583	37.47	[1, 77, 1, 2504]	CT CA CG	0.97
nad4	376	C	2	2661	37.67	[0, 102, 0, 2559]	CT	0.96

nad4	403	C	2	2872	37.58	[1, 139, 1, 2731]	CT CA CG	0.95
nad4	416	C	2	2771	37.55	[0, 145, 0, 2626]	CT	0.95
nad4	433	C	2	2686	37.5	[0, 183, 0, 2503]	CT	0.93
nad4	436	C	2	2642	37.6	[0, 91, 0, 2551]	CT	0.97
nad4	437	C	2	2651	37.51	[0, 112, 0, 2539]	CT	0.96
nad4	449	C	2	3219	37.63	[8, 108, 2, 3101]	CT CA CG	0.97
nad4	608	C	2	5144	37.68	[1, 83, 0, 5060]	CT CA	0.98
nad4	659	C	2	4894	37.39	[0, 195, 0, 4699]	CT	0.96
nad4	767	C	2	5954	37.48	[2, 124, 0, 5828]	CT CA	0.98
nad4	819	C	2	5533	37.18	[0, 4724, 0, 809]	CT	0.15
nad4	836	C	2	4901	37.16	[0, 96, 0, 4805]	CT	0.98
nad4	837	C	2	4813	37.21	[0, 4269, 0, 544]	CT	0.11
nad4	857	C	2	4918	37.69	[6, 136, 2, 4774]	CT CA CG	0.97
nad4	896	C	2	4090	37.7	[0, 80, 0, 4010]	CT	0.98
nad4	977	C	2	3660	37.52	[0, 643, 21, 2996]	CT CG	0.82
nad4	1006	C	2	3319	37.54	[1, 280, 0, 3038]	CT CA	0.92
nad4	1007	C	2	3346	37.66	[0, 163, 0, 3183]	CT	0.95
nad4	1009	C	2	3286	37.43	[1, 212, 0, 3073]	CT CA	0.94
nad4	1010	C	2	3365	37.69	[0, 288, 0, 3077]	CT	0.91
nad4	1101	C	2	5410	37.33	[1, 1994, 0, 3415]	CT CA	0.63
nad4	1109	C	2	5406	37.55	[0, 223, 0, 5183]	CT	0.96
nad4	1129	C	2	5465	37.22	[1, 161, 1, 5302]	CT CG CA	0.97
nad4	1132	C	2	5606	37.33	[0, 155, 0, 5451]	CT	0.97
nad4	1142	C	2	5422	37.38	[1, 186, 0, 5235]	CT CA	0.97
nad4	1148	C	2	5189	37.34	[0, 332, 0, 4857]	CT	0.94
nad4	1151	C	2	5151	37.35	[0, 246, 0, 4905]	CT	0.95
nad4	1172	C	2	5147	37.48	[2, 112, 0, 5033]	CT CA	0.98
nad4	1194	C	2	4897	37.01	[1, 4203, 0, 693]	CT CA	0.14
nad4	1205	C	2	5199	37.37	[1, 151, 0, 5047]	CT CA	0.97
nad4	1355	C	2	5931	37.72	[1, 48, 0, 5882]	CT CA	0.99
nad4	1373	C	2	6422	37.37	[1, 142, 0, 6279]	CT CA	0.98
nad4	1417	C	2	4986	37.44	[0, 93, 0, 4893]	CT	0.98
nad4	1433	C	2	4465	37.56	[4, 182, 7, 4272]	CT CG CA	0.96
nad4L	8	C	2	5333	37.61	[0, 1127, 0, 4206]	CT	0.79

nad4L	21	C	2	5350	37.32	[0, 4776, 0, 574]	CT	0.11
nad4L	41	C	2	5502	37.62	[0, 528, 0, 4974]	CT	0.9
nad4L	55	C	2	5077	37.61	[5, 791, 3, 4278]	CT CA CG	0.84
nad4L	86	C	2	5229	37.59	[1, 246, 0, 4982]	CT CA	0.95
nad4L	95	C	2	5046	37.61	[2, 436, 0, 4608]	CT CA	0.91
nad4L	100	C	2	4812	37.11	[0, 284, 0, 4528]	CT	0.94
nad4L	110	C	2	5037	37.63	[0, 469, 0, 4568]	CT	0.91
nad4L	158	C	2	7013	37.68	[1, 241, 0, 6771]	CT CA	0.97
nad4L	179	C	2	7740	37.63	[0, 248, 2, 7490]	CT CG	0.97
nad4L	188	C	2	7805	37.58	[0, 1311, 0, 6494]	CT	0.83
nad4L	196	C	2	7590	37.04	[0, 5598, 0, 1992]	CT	0.26
nad4L	197	C	2	7949	37.56	[2, 1460, 0, 6487]	CT CA	0.82
nad4L	281	C	2	8622	37.49	[1, 202, 0, 8419]	CT CA	0.98
nad5	150	C	2	3300	36.97	[0, 2732, 0, 568]	CT	0.17
nad5	155	C	2	3500	37.52	[0, 406, 2, 3092]	CT CG	0.88
nad5	242	C	2	3226	37.45	[1, 94, 1, 3130]	CT CA CG	0.97
nad5	358	C	2	2524	37.54	[1, 74, 1, 2448]	CT CA CG	0.97
nad5	374	C	2	2624	37.66	[0, 179, 0, 2445]	CT	0.93
nad5	398	C	2	2706	37.45	[0, 127, 0, 2579]	CT	0.95
nad5	494	C	2	3936	37.58	[2, 243, 0, 3691]	CT CA	0.94
nad5	506	C	2	4611	37.48	[0, 239, 0, 4372]	CT	0.95
nad5	539	C	2	5153	37.53	[1, 399, 1, 4752]	CT CA CG	0.92
nad5	548	C	2	5332	37.66	[0, 236, 1, 5095]	CT CG	0.96
nad5	553	C	2	5585	37.52	[0, 405, 0, 5180]	CT	0.93
nad5	598	C	2	5587	37.61	[2, 126, 1, 5458]	CT CA CG	0.98
nad5	608	C	2	5708	37.03	[1, 236, 1, 5470]	CT CA CG	0.96
nad5	676	C	2	6759	37.59	[0, 132, 0, 6627]	CT	0.98
nad5	713	C	2	7117	37.48	[1, 262, 0, 6854]	CT CA	0.96
nad5	725	C	2	7170	37.46	[3, 287, 3, 6877]	CT CA CG	0.96
nad5	835	C	2	7294	37.37	[1, 192, 0, 7101]	CT CA	0.97
nad5	863	C	2	6338	37.42	[0, 240, 0, 6098]	CT	0.96
nad5	875	C	2	6421	37.59	[3, 428, 1, 5989]	CT CA CG	0.93
nad5	1048	C	2	4329	36.65	[0, 3718, 0, 611]	CT	0.14
nad5	1400	C	2	4562	37.65	[0, 175, 0, 4387]	CT	0.96

nad5	1490	C	2	6700	37.43	[2, 227, 0, 6471]	CT CA	0.97
nad5	1550	C	2	7760	37.3	[1, 261, 1, 7497]	CT CA CG	0.97
nad5	1568	C	2	7429	37.71	[0, 286, 0, 7143]	CT	0.96
nad5	1580	C	2	7442	37.68	[0, 350, 0, 7092]	CT	0.95
nad5	1589	C	2	7563	37.54	[1, 268, 0, 7294]	CT CA	0.96
nad5	1610	C	2	8017	37.5	[1, 399, 0, 7617]	CT CA	0.95
nad5	1665	C	2	8771	37.2	[6, 5814, 4, 2947]	CT CA CG	0.34
nad5	1674	C	2	9753	36.84	[2, 8682, 0, 1069]	CT CA	0.11
nad5	1916	C	2	6519	37.52	[0, 192, 0, 6327]	CT	0.97
nad5	1918	C	2	6525	37.71	[0, 315, 1, 6209]	CT CG	0.95
nad5	1929	C	2	5567	36.79	[6, 4475, 9, 1077]	CT CG CA	0.19
nad5	1958	C	2	5440	37.54	[0, 104, 1, 5335]	CT CG	0.98
nad6	26	C	2	7635	37.5	[2, 323, 1, 7309]	CT CA CG	0.96
nad6	53	C	2	10207	37.42	[0, 216, 0, 9991]	CT	0.98
nad6	88	C	2	9545	37.51	[1, 208, 0, 9336]	CT CA	0.98
nad6	89	C	2	9535	37.5	[1, 279, 0, 9255]	CT CA	0.97
nad6	95	C	2	10275	37.73	[2, 393, 6, 9874]	CT CG CA	0.96
nad6	103	C	2	10448	37.66	[1, 489, 2, 9956]	CT CG CA	0.95
nad6	105	C	2	10048	37.13	[0, 7100, 0, 2948]	CT	0.29
nad6	146	C	2	12532	37.48	[0, 114, 1, 12417]	CT CG	0.99
nad6	159	C	2	11835	37.47	[4, 373, 1, 11457]	CT CA CG	0.97
nad6	161	C	2	11897	37.66	[0, 1953, 0, 9944]	CT	0.84
nad6	191	C	2	10243	37.65	[0, 40, 0, 10203]	CT	1
nad6	288	C	2	13618	37.06	[0, 7578, 2, 6038]	CT CG	0.44
nad6	289	C	2	14065	37.49	[0, 69, 2, 13994]	CT CG	1
nad6	446	C	2	12787	37.15	[0, 50, 0, 12737]	CT	1
nad7	38	C	2	4166	37.68	[2, 398, 0, 3766]	CT CA	0.9
nad7	77	C	2	3546	37.71	[0, 187, 1, 3358]	CT CG	0.95
nad7	83	C	2	3601	37.67	[0, 155, 0, 3446]	CT	0.96
nad7	200	C	2	2058	37.56	[0, 113, 0, 1945]	CT	0.95
nad7	224	C	2	3355	37.49	[0, 1125, 0, 2230]	CT	0.66
nad7	244	C	2	3519	37.63	[1, 102, 0, 3416]	CT CA	0.97
nad7	251	C	2	3427	37.72	[0, 102, 0, 3325]	CT	0.97
nad7	316	C	2	3802	37.73	[1, 85, 1, 3715]	CT CA CG	0.98

nad7	335	C	2	4508	37.6	[0, 72, 0, 4436]	CT	0.98
nad7	344	C	2	4742	37.62	[1, 109, 0, 4632]	CT CA	0.98
nad7	383	C	2	5521	37.56	[1, 111, 0, 5409]	CT CA	0.98
nad7	533	C	2	9356	37.26	[1, 264, 1, 9090]	CT CG CA	0.97
nad7	578	C	2	8326	37.41	[1, 122, 0, 8203]	CT CA	0.99
nad7	657	C	2	5815	37.28	[1, 3998, 1, 1815]	CT CG CA	0.31
nad7	679	C	2	5724	37.07	[13, 500, 15, 5196]	CT CG CA	0.91
nad7	698	C	2	6496	37.63	[3, 354, 1, 6138]	CT CA CG	0.95
nad7	724	C	2	6980	37.71	[0, 98, 0, 6882]	CT	0.99
nad7	734	C	2	7032	37.74	[2, 90, 2, 6938]	CT CA CG	0.99
nad7	739	C	2	6999	37.6	[2, 179, 0, 6818]	CT CA	0.97
nad7	769	C	2	8270	37.38	[3, 214, 1, 8052]	CT CA CG	0.97
nad7	789	C	2	8616	37.18	[0, 1737, 0, 6879]	CT	0.8
nad7	926	C	2	8422	36.91	[1, 1184, 1, 7236]	CT CA CG	0.86
nad7	963	C	2	7765	36.91	[0, 4628, 2, 3135]	CT CG	0.4
nad7	973	C	2	7791	37.11	[1, 118, 0, 7672]	CT CA	0.98
nad7	1050	C	2	7174	37.18	[17, 67, 3, 7087]	CT CA CG	0.99
nad7	1057	C	2	6804	37.29	[3, 71, 4, 6726]	CT CG CA	0.99
nad7	1079	C	2	7969	36.61	[4, 51, 5, 7909]	CT CG CA	0.99
nad7	1088	C	2	8712	37.37	[1, 257, 1, 8453]	CT CG CA	0.97
nad7	1103	C	2	8104	37.2	[0, 196, 0, 7908]	CT	0.98
nad7	1119	C	2	7508	37.24	[1, 6570, 0, 937]	CT CA	0.12
nad7	1124	C	2	7004	37.37	[13, 184, 9, 6798]	CT CA CG	0.97
nad7	1137	C	2	5622	37.33	[3, 1036, 1, 4582]	CT CA CG	0.82
nad7	1166	C	2	4692	36.86	[0, 31, 2, 4659]	CT CG	0.99
nad9	92	C	2	24420	37.4	[2, 391, 0, 24027]	CT CA	0.98
nad9	113	C	2	20791	37.68	[57, 415, 11, 20308]	CT CA CG	0.98
nad9	167	C	2	24499	37.66	[1, 226, 0, 24272]	CT CA	0.99
nad9	190	C	2	23196	37.53	[1, 399, 5, 22791]	CT CG CA	0.98
nad9	328	C	2	22412	37.39	[47, 216, 18, 22131]	CT CA CG	0.99
nad9	368	C	2	27811	37.44	[5, 254, 0, 27552]	CT CA	0.99
nad9	398	C	2	27421	37.66	[8, 614, 0, 26799]	CT CA	0.98
nad9	439	C	2	26264	37.21	[8, 808, 5, 25443]	CT CA CG	0.97
rpl10	9	C	2	2164	37.5	[1, 1124, 0, 1039]	CT CA	0.48

rpl10	83	C	2	5685	37.66	[1, 642, 0, 5042]	CT CA	0.89
rpl10	101	C	2	6279	37.58	[0, 612, 0, 5667]	CT	0.9
rpl10	133	C	2	7300	37.4	[2, 172, 0, 7126]	CT CA	0.98
rpl10	134	C	2	7380	37.52	[1, 277, 0, 7102]	CT CA	0.96
rpl10	239	C	2	8493	37.68	[0, 348, 0, 8145]	CT	0.96
rpl10	314	C	2	9299	37.72	[1, 94, 0, 9204]	CT CA	0.99
rpl10	330	C	2	7465	37.26	[0, 1785, 1, 5679]	CT CG	0.76
rpl10	397	C	2	8612	37.06	[0, 4012, 0, 4600]	CT	0.53
rpl16	104	C	2	9117	37.38	[5, 227, 0, 8885]	CT CA	0.98
rpl16	141	C	2	10172	37.4	[0, 9040, 1, 1131]	CT CG	0.11
rpl16	164	C	2	9070	37.54	[1, 2269, 0, 6800]	CT CA	0.75
rpl16	174	C	2	7910	37.17	[2, 4554, 0, 3354]	CT CA	0.42
rpl16	232	C	2	5067	37.06	[1, 1041, 0, 4025]	CT CA	0.79
rpl16	401	C	2	4890	37.68	[2, 801, 0, 4087]	CT CA	0.84
rpl16	407	C	2	5233	37.59	[2, 1560, 0, 3671]	CT CA	0.7
rpl5	7	C	2	2981	37.39	[0, 891, 0, 2090]	CT	0.7
rpl5	35	C	2	3425	37.64	[1, 476, 0, 2948]	CT CA	0.86
rpl5	59	C	2	4174	37.65	[0, 603, 0, 3571]	CT	0.86
rpl5	92	C	2	4247	37.57	[0, 503, 0, 3744]	CT	0.88
rpl5	335	C	2	4473	37.56	[0, 1288, 0, 3185]	CT	0.71
rpl5	518	C	2	2930	37.56	[0, 459, 0, 2471]	CT	0.84
rps1	209	C	2	1098	37.07	[0, 55, 0, 1043]	CT	0.95
rps1	605	C	2	826	36.94	[1, 38, 0, 787]	CT CA	0.95
rps10	2	C	2	5858	37.37	[11, 1383, 3, 4456]	CT CA CG	0.76
rps10	238	C	2	5513	37.53	[1, 1347, 0, 4165]	CT CA	0.76
rps10	302	C	2	8266	37.39	[0, 1690, 0, 6576]	CT	0.8
rps10	331	C	2	8002	37.46	[1, 251, 1, 7749]	CT CG CA	0.97
rps12	284	C	2	777	36.97	[0, 223, 0, 554]	CT	0.71
rps14	194	C	2	6133	37.27	[0, 297, 0, 5836]	CT	0.95
rps14	271	C	2	6573	37.12	[0, 253, 0, 6320]	CT	0.96
rps19	126	C	2	2241	37.27	[0, 1619, 0, 622]	CT	0.28
rps19	137	C	2	2201	37.2	[0, 1646, 0, 555]	CT	0.25
rps19	138	C	2	2253	37.52	[0, 705, 0, 1548]	CT	0.69
rps19	163	C	2	2087	37.28	[0, 704, 0, 1383]	CT	0.66

rps19	164	C	2	2172	37.51	[0, 196, 0, 1976]	CT	0.91
rps19	260	C	2	1030	36.95	[1, 914, 0, 115]	CT CA	0.11
rps3	26	C	2	872	37.5	[0, 724, 0, 148]	CT	0.17
rps3	69	C	2	1015	37.52	[0, 690, 0, 325]	CT	0.32
rps3	92	C	2	2722	37.68	[0, 98, 0, 2624]	CT	0.96
rps3	96	C	2	2737	37.31	[0, 2004, 0, 733]	CT	0.27
rps3	171	C	2	3092	37.22	[1, 2769, 0, 322]	CT CA	0.1
rps3	441	C	2	4103	37.12	[0, 3618, 0, 485]	CT	0.12
rps3	518	C	2	5831	37.6	[1, 483, 0, 5347]	CT CA	0.92
rps3	737	C	2	4629	37.48	[0, 1415, 0, 3214]	CT	0.69
rps3	914	C	2	3454	37.37	[0, 1198, 1, 2255]	CT CG	0.65
rps3	1388	C	2	4619	37.57	[0, 486, 0, 4133]	CT	0.89
rps3	1415	C	2	4212	37.51	[11, 139, 6, 4056]	CT CA CG	0.97
rps3	1526	C	2	15912	37.55	[0, 12114, 1, 3797]	CT CG	0.24
rps3	1612	C	2	14690	37.22	[2, 725, 3, 13960]	CT CG CA	0.95
rps4	38	C	2	927	37.63	[0, 159, 0, 768]	CT	0.83
rps4	49	C	2	1020	37.67	[0, 191, 1, 828]	CT CG	0.81
rps4	482	C	2	1795	37.61	[0, 238, 0, 1557]	CT	0.87
rps4	668	C	2	2027	36.84	[0, 1762, 1, 264]	CT CG	0.13
rps4	894	C	2	1387	37.04	[1, 1044, 0, 342]	CT CA	0.25
rps4	914	C	2	1325	37.63	[0, 157, 0, 1168]	CT	0.88
rps4	925	C	2	1516	37.59	[0, 77, 0, 1439]	CT	0.95
rps4	935	C	2	1508	37.61	[0, 171, 1, 1336]	CT CG	0.89
rps4	950	C	2	1569	37.39	[0, 64, 0, 1505]	CT	0.96
rps4	1000	C	2	1287	37.38	[0, 187, 0, 1100]	CT	0.85
rps4	1001	C	2	1314	37.63	[0, 77, 0, 1237]	CT	0.94
rps4	1010	C	2	1400	37.67	[0, 88, 0, 1312]	CT	0.94
rps4	1015	C	2	1365	37.72	[1, 65, 0, 1299]	CT CA	0.95
rps4	1026	C	2	1407	37.24	[0, 999, 0, 408]	CT	0.29
rps7	116	C	2	2269	37.59	[4, 129, 1, 2135]	CT CA CG	0.94
rps7	177	C	2	2187	37.17	[3, 1912, 0, 272]	CT CA	0.12
rps7	309	C	2	1945	37.38	[0, 1674, 0, 271]	CT	0.14
rps7	332	C	2	1851	37.46	[0, 72, 0, 1779]	CT	0.96
rps7	426	C	2	1614	37.26	[1, 1317, 0, 296]	CT CA	0.18

sdh4	29	C	2	7608	37.77	[1, 24, 5, 7578]	CT CG CA	1
sdh4	155	C	2	28019	37.66	[2, 815, 0, 27202]	CT CA	0.97
sdh4	203	C	2	25138	37.7	[7, 199, 1, 24931]	CT CA CG	0.99
sdh4	259	C	2	17101	37.56	[7, 181, 0, 16913]	CT CA	0.99
sdh4	348	C	2	5316	36.6	[0, 4264, 2, 1050]	CT CG	0.2
sdh4	353	C	2	5428	37.18	[0, 158, 0, 5270]	CT	0.97

Table S10. RNA editing events predicted in *Punica granatum* mitochondrial genome based on online website PREPACT

Gene	Base	Aa	Triplet pos.	Bases	Codon	Aa change
atp1	302	101	2	C→U	GCG→GUG	A→V
atp1	488	163	2	C→U	GCG→GUG	A→V
atp1	514	172	1	C→U	CGU→UGU	R→C
atp1	752	251	2	C→U	GCU→GUU	A→V
atp1	799	267	1	C→U	CCU→UCU	P→S
atp1	1316	439	2	C→U	GCC→GUC	A→V
atp4	59	20	2	C→U	UCU→UUU	S→F
atp4	89	30	2	C→U	UCA→UUA	S→L
atp4	118	40	1	C→U	CGU→UGU	R→C
atp4	215	72	2	C→U	UCG→UUG	S→L
atp4	227	76	2	C→U	CCC→CUC	P→L
atp4	248	83	2	C→U	CCU→CUU	P→L
atp4	251	84	2	C→U	CCG→CUG	P→L
atp4	395	132	2	C→U	UCA→UUA	S→L
atp4	407	136	2	C→U	CCA→CUA	P→L
atp4	416	139	2	C→U	ACU→AUU	T→I
atp6	119	40	2	C→U	ACU→AUU	T→I
atp6	329	110	2	C→U	CCC→CUC	P→L
atp6	373	125	1	C→U	CUC→UUC	L→F
atp6	406	136	1	C→U	CUU→UUU	L→F
atp6	461	154	2	C→U	UCA→UUA	S→L
atp6	520	174	1	C→U	CCU→UCU	P→S
atp6	523	175	1	C→U	CAU→UAU	H→Y
atp6	545	182	2	C→U	UCA→UUA	S→L
atp6	587	196	2	C→U	UCA→UUA	S→L
atp6	608	203	2	C→U	UCC→UUC	S→F
atp6	730	244	1	C→U	CAU→UAU	H→Y
atp6	737	246	2	C→U	UCU→UUU	S→F
atp6	746	249	2	C→U	UCA→UUA	S→L
atp8	47	16	2	C→U	UCA→UUA	S→L
atp8	58	20	1	C→U	CUC→UUC	L→F

atp8	76	26	1	C→U	CCC→UCC	P→S
atp9	50	17	2	C→U	UCA→UUA	S→L
atp9	59	20	2	C→U	GCU→GUU	A→V
atp9	200	67	2	C→U	GCC→GUC	A→V
ccmB	28	10	1	C→U	CAU→UAU	H→Y
ccmB	71	24	2	C→U	CCG→CUG	P→L
ccmB	80	27	2	C→U	UCG→UUG	S→L
ccmB	128	43	2	C→U	UCA→UUA	S→L
ccmB	133	45	1	C→U	CAU→UAU	H→Y
ccmB	137	46	2	C→U	UCC→UUC	S→F
ccmB	149	50	2	C→U	CCG→CUG	P→L
ccmB	160	54	1	C→U	CCU→UCU	P→S
ccmB	164	55	2	C→U	CCG→CUG	P→L
ccmB	172	58	1	C→U	CCU→UCU	P→S
ccmB	179	60	2	C→U	CCU→CUU	P→L
ccmB	181	61	1	C→U	CCC→UCC	P→S
ccmB	193	65	1	C→U	CCU→UCU	P→S
ccmB	194	65	2	C→U	UCU→UUU	S→F
ccmB	286	96	1	C→U	CGG→UGG	R→W
ccmB	304	102	1	C→U	CGU→UGU	R→C
ccmB	313	105	1	C→U	CGU→UGU	R→C
ccmB	367	123	1	C→U	CGG→UGG	R→W
ccmB	392	131	2	C→U	CCG→CUG	P→L
ccmB	424	142	1	C→U	CGU→UGU	R→C
ccmB	428	143	2	C→U	UCG→UUG	S→L
ccmB	467	156	2	C→U	UCG→UUG	S→L
ccmB	476	159	2	C→U	CCA→CUA	P→L
ccmB	485	162	2	C→U	UCA→UUA	S→L
ccmB	494	165	2	C→U	UCA→UUA	S→L
ccmB	503	168	2	C→U	CCA→CUA	P→L
ccmB	506	169	2	C→U	ACC→AUC	T→I
ccmB	512	171	2	C→U	UCU→UUU	S→F
ccmB	514	172	1	C→U	CGU→UGU	R→C
ccmB	551	184	2	C→U	UCA→UUA	S→L

ccmB	554	185	2	C→U	UCG→UUG	S→L
ccmB	566	189	2	C→U	UCC→UUC	S→F
ccmB	569	190	2	C→U	UCU→UUU	S→F
ccmB	572	191	2	C→U	CCG→CUG	P→L
ccmB	596	199	2	C→U	UCG→UUG	S→L
ccmC	103	35	1	C→U	CAU→UAU	H→Y
ccmC	115	39	1	C→U	CGG→UGG	R→W
ccmC	161	54	2	C→U	CCG→CUG	P→L
ccmC	179	60	2	C→U	GCG→GUG	A→V
ccmC	184	62	1	C→U	CGG→UGG	R→W
ccmC	266	89	2	C→U	UCU→UUU	S→F
ccmC	331	111	1	C→U	CGG→UGG	R→W
ccmC	395	132	2	C→U	UCG→UUG	S→L
ccmC	400	134	1	C→U	CUU→UUU	L→F
ccmC	436	146	1	C→U	CCU→UCU	P→S
ccmC	446	149	2	C→U	CCG→CUG	P→L
ccmC	458	153	2	C→U	UCA→UUA	S→L
ccmC	463	155	1	C→U	CGU→UGU	R→C
ccmC	467	156	2	C→U	GCU→GUU	A→V
ccmC	473	158	2	C→U	CCG→CUG	P→L
ccmC	497	166	2	C→U	UCU→UUU	S→F
ccmC	521	174	2	C→U	UCG→UUG	S→L
ccmC	548	183	2	C→U	UCU→UUU	S→F
ccmC	568	190	1	C→U	CCU→UCU	P→S
ccmC	575	192	2	C→U	CCC→CUC	P→L
ccmC	605	202	2	C→U	UCC→UUC	S→F
ccmC	608	203	2	C→U	CCC→CUC	P→L
ccmC	614	205	2	C→U	UCA→UUA	S→L
ccmC	619	207	1	C→U	CGU→UGU	R→C
ccmC	650	217	2	C→U	CCU→CUU	P→L
ccmC	665	222	2	C→U	CCC→CUC	P→L
ccmFC	38	13	2	C→U	UCC→UUC	S→F
ccmFC	50	17	2	C→U	CCU→CUU	P→L
ccmFC	52	18	1	C→U	CGU→UGU	R→C

ccmFC	100	34	1	C→U	CCC→UCC	P→S
ccmFC	104	35	2	C→U	ACA→AUA	T→I
ccmFC	116	39	2	C→U	UCU→UUU	S→F
ccmFC	119	40	2	C→U	UCC→UUC	S→F
ccmFC	143	48	2	C→U	CCU→CUU	P→L
ccmFC	148	50	1	C→U	CCU→UCU	P→S
ccmFC	152	51	2	C→U	UCA→UUA	S→L
ccmFC	157	53	1	C→U	CCU→UCU	P→S
ccmFC	160	54	1	C→U	CUU→UUU	L→F
ccmFC	293	98	2	C→U	UCA→UUA	S→L
ccmFC	298	100	1	C→U	CGU→UGU	R→C
ccmFC	322	108	1	C→U	CUU→UUU	L→F
ccmFC	335	112	2	C→U	UCC→UUC	S→F
ccmFC	379	127	1	C→U	CGU→UGU	R→C
ccmFC	394	132	1	C→U	CGU→UGU	R→C
ccmFC	400	134	1	C→U	CUC→UUC	L→F
ccmFC	407	136	2	C→U	UCU→UUU	S→F
ccmFC	587	196	2	C→U	UCG→UUG	S→L
ccmFC	701	234	2	C→U	CCA→CUA	P→L
ccmFC	872	291	2	C→U	UCU→UUU	S→F
ccmFC	925	309	1	C→U	CGG→UGG	R→W
ccmFC	1012	338	1	C→U	CAU→UAU	H→Y
ccmFC	1133	378	2	C→U	CCA→CUA	P→L
ccmFC	1172	391	2	C→U	UCC→UUC	S→F
ccmFC	1228	410	1	C→U	CGG→UGG	R→W
ccmFC	1262	421	2	C→U	UCG→UUG	S→L
ccmFC	1309	437	1	C→U	CGA→UGA	R→*
ccmFN	29	10	2	C→U	UCG→UUG	S→L
ccmFN	37	13	1	C→U	CCG→UCG	P→S
ccmFN	98	33	2	C→U	CCC→CUC	P→L
ccmFN	137	46	2	C→U	UCG→UUG	S→L
ccmFN	142	48	1	C→U	CGU→UGU	R→C
ccmFN	151	51	1	C→U	CCG→UCG	P→S
ccmFN	248	83	2	C→U	UCA→UUA	S→L

ccmFN	256	86	1	C→U	CGG→UGG	R→W
ccmFN	263	88	2	C→U	CCA→CUA	P→L
ccmFN	356	119	2	C→U	UCC→UUC	S→F
ccmFN	544	182	1	C→U	CCU→UCU	P→S
ccmFN	688	230	1	C→U	CCU→UCU	P→S
ccmFN	689	230	2	C→U	UCU→UUU	S→F
ccmFN	736	246	1	C→U	CGU→UGU	R→C
ccmFN	758	253	2	C→U	UCA→UUA	S→L
ccmFN	770	257	2	C→U	CCA→CUA	P→L
ccmFN	785	262	2	C→U	UCA→UUA	S→L
ccmFN	934	312	1	C→U	CGU→UGU	R→C
ccmFN	1055	352	2	C→U	CCG→CUG	P→L
ccmFN	1178	393	2	C→U	UCG→UUG	S→L
ccmFN	1181	394	2	C→U	CCU→CUU	P→L
ccmFN	1261	421	1	C→U	CGG→UGG	R→W
ccmFN	1289	430	2	C→U	CCA→CUA	P→L
ccmFN	1306	436	1	C→U	CAU→UAU	H→Y
ccmFN	1321	441	1	C→U	CGG→UGG	R→W
ccmFN	1339	447	1	C→U	CGG→UGG	R→W
ccmFN	1372	458	1	C→U	CGG→UGG	R→W
ccmFN	1390	464	1	C→U	CGU→UGU	R→C
ccmFN	1414	472	1	C→U	CUU→UUU	L→F
ccmFN	1433	478	2	C→U	UCG→UUG	S→L
ccmFN	1453	485	1	C→U	CUU→UUU	L→F
ccmFN	1481	494	2	C→U	UCA→UUA	S→L
ccmFN	1523	508	2	C→U	ACA→AUA	T→I
cox1	92	31	2	C→U	ACU→AUU	T→I
cox1	134	45	2	C→U	ACA→AUA	T→I
cox1	199	67	1	C→U	CAU→UAU	H→Y
cox1	205	69	1	C→U	CUU→UUU	L→F
cox1	281	94	2	C→U	UCU→UUU	S→F
cox1	293	98	2	C→U	UCU→UUU	S→F
cox1	391	131	1	C→U	CCA→UCA	P→S
cox1	485	162	2	C→U	GCA→GUA	A→V

cox1	491	164	2	C→U	UCU→UUU	S→F
cox1	496	166	1	C→U	CUU→UUU	L→F
cox1	554	185	2	C→U	UCC→UUC	S→F
cox1	590	197	2	C→U	UCA→UUA	S→L
cox1	614	205	2	C→U	CCA→CUA	P→L
cox1	629	210	2	C→U	CCA→CUA	P→L
cox1	754	252	1	C→U	CGG→UGG	R→W
cox1	785	262	2	C→U	CCC→CUC	P→L
cox1	800	267	2	C→U	UCC→UUC	S→F
cox1	1076	359	2	C→U	UCC→UUC	S→F
cox1	1118	373	2	C→U	CCG→CUG	P→L
cox1	1225	409	1	C→U	CAC→UAC	H→Y
cox1	1313	438	2	C→U	CCG→CUG	P→L
cox1	1318	440	1	C→U	CUC→UUC	L→F
cox2	229	77	1	C→U	CCU→UCU	P→S
cox2	260	87	2	C→U	CCC→CUC	P→L
cox2	326	109	2	C→U	GCU→GUU	A→V
cox2	346	116	1	C→U	CGG→UGG	R→W
cox2	521	174	2	C→U	GCA→GUA	A→V
cox2	535	179	1	C→U	CGU→UGU	R→C
cox2	550	184	1	C→U	CCU→UCU	P→S
cox2	704	235	2	C→U	ACG→AUG	T→M
cox3	262	88	1	C→U	CAC→UAC	H→Y
cox3	266	89	2	C→U	CCA→CUA	P→L
cox3	289	97	1	C→U	CUC→UUC	L→F
cox3	301	101	1	C→U	CUC→UUC	L→F
cox3	385	129	1	C→U	CAU→UAU	H→Y
cox3	469	157	1	C→U	CGG→UGG	R→W
cox3	473	158	2	C→U	GCU→GUU	A→V
cox3	524	175	2	C→U	CCA→CUA	P→L
cox3	547	183	1	C→U	CCU→UCU	P→S
cox3	548	183	2	C→U	UCU→UUU	S→F
cox3	613	205	1	C→U	CAU→UAU	H→Y
cox3	616	206	1	C→U	CAU→UAU	H→Y

cox3	815	272	2	C→U	UCG→UUG	S→L
cox3	916	306	1	C→U	CGG→UGG	R→W
cob	37	13	1	C→U	CCU→UCU	P→S
cob	38	13	2	C→U	UCU→UUU	S→F
cob	283	95	1	C→U	CUC→UUC	L→F
cob	295	99	1	C→U	CAC→UAC	H→Y
cob	355	119	1	C→U	CGG→UGG	R→W
cob	473	158	2	C→U	GCC→GUC	A→V
cob	853	285	1	C→U	CAU→UAU	H→Y
cob	875	292	2	C→U	CCU→CUU	P→L
cob	908	303	2	C→U	CCA→CUA	P→L
cob	1045	349	1	C→U	CCU→UCU	P→S
cob	1046	349	2	C→U	UCU→UUU	S→F
matR	166	56	1	C→U	CAC→UAC	H→Y
matR	236	79	2	C→U	UCC→UUC	S→F
matR	292	98	1	C→U	CCC→UCC	P→S
matR	317	106	2	C→U	UCG→UUG	S→L
matR	413	138	2	C→U	UCG→UUG	S→L
matR	481	161	1	C→U	CCC→UCC	P→S
matR	482	161	2	C→U	UCC→UUC	S→F
matR	899	300	2	C→U	UCA→UUA	S→L
matR	1519	507	1	C→U	CCC→UCC	P→S
matR	1655	552	2	C→U	UCC→UUC	S→F
matR	1676	559	2	C→U	CCU→CUU	P→L
matR	1732	578	1	C→U	CAC→UAC	H→Y
matR	1820	607	2	C→U	UCA→UUA	S→L
mttB	74	25	2	C→U	UCC→UUC	S→F
mttB	79	27	1	C→U	CGG→UGG	R→W
mttB	94	32	1	C→U	CUU→UUU	L→F
mttB	127	43	1	C→U	CCG→UCG	P→S
mttB	143	48	2	C→U	UCU→UUU	S→F
mttB	146	49	2	C→U	CCA→CUA	P→L
mttB	203	68	2	C→U	UCA→UUA	S→L
mttB	217	73	1	C→U	CCG→UCG	P→S

mttB	239	80	2	C→U	UCA→UUA	S→L
mttB	251	84	2	C→U	UCU→UUU	S→F
mttB	277	93	1	C→U	CAU→UAU	H→Y
mttB	346	116	1	C→U	CAU→UAU	H→Y
mttB	359	120	2	C→U	UCU→UUU	S→F
mttB	368	123	2	C→U	UCC→UUC	S→F
mttB	388	130	1	C→U	CCU→UCU	P→S
mttB	389	130	2	C→U	UCU→UUU	S→F
mttB	391	131	1	C→U	CCC→UCC	P→S
mttB	394	132	1	C→U	CGG→UGG	R→W
mttB	422	141	2	C→U	UCA→UUA	S→L
mttB	452	151	2	C→U	UCG→UUG	S→L
mttB	512	171	2	C→U	UCG→UUG	S→L
mttB	520	174	1	C→U	CCC→UCC	P→S
mttB	563	188	2	C→U	CCA→CUA	P→L
mttB	569	190	2	C→U	CCU→CUU	P→L
mttB	625	209	1	C→U	CCG→UCG	P→S
mttB	647	216	2	C→U	UCC→UUC	S→F
mttB	682	228	1	C→U	CGU→UGU	R→C
mttB	728	243	2	C→U	UCG→UUG	S→L
nad1	208	70	1	C→U	CCA→UCA	P→S
nad1	299	100	2	C→U	UCA→UUA	S→L
nad1	430	144	1	C→U	CGG→UGG	R→W
nad1	467	156	2	C→U	UCA→UUA	S→L
nad1	541	181	1	C→U	CGG→UGG	R→W
nad1	635	212	2	C→U	ACU→AUU	T→I
nad1	803	268	2	C→U	UCA→UUA	S→L
nad1	842	281	2	C→U	UCU→UUU	S→F
nad1	847	283	1	C→U	CUU→UUU	L→F
nad1	904	302	1	C→U	CUC→UUC	L→F
nad1	938	313	2	C→U	CCG→CUG	P→L
nad1	1045	349	1	C→U	CGG→UGG	R→W
nad2	347	116	2	C→U	ACA→AUA	T→I
nad2	482	161	2	C→U	UCA→UUA	S→L

nad2	578	193	2	C→U	UCG→UUG	S→L
nad2	604	202	1	C→U	CCC→UCC	P→S
nad2	770	257	2	C→U	GCA→GUA	A→V
nad2	793	265	1	C→U	CCU→UCU	P→S
nad2	815	272	2	C→U	CCC→CUC	P→L
nad2	818	273	2	C→U	ACC→AUC	T→I
nad2	881	294	2	C→U	UCC→UUC	S→F
nad2	1043	348	2	C→U	ACU→AUU	T→I
nad2	1100	367	2	C→U	UCA→UUA	S→L
nad2	1169	390	2	C→U	ACC→AUC	T→I
nad2	1310	437	2	C→U	GCC→GUC	A→V
nad3	56	19	2	C→U	UCU→UUU	S→F
nad3	77	26	2	C→U	CCG→CUG	P→L
nad3	167	56	2	C→U	UCC→UUC	S→F
nad3	229	77	1	C→U	CCU→UCU	P→S
nad3	230	77	2	C→U	UCU→UUU	S→F
nad3	245	82	2	C→U	ACC→AUC	T→I
nad3	271	91	1	C→U	CCC→UCC	P→S
nad3	272	91	2	C→U	UCC→UUC	S→F
nad3	287	96	2	C→U	CCG→CUG	P→L
nad3	296	99	2	C→U	UCU→UUU	S→F
nad3	338	113	2	C→U	UCU→UUU	S→F
nad3	340	114	1	C→U	CUC→UUC	L→F
nad4	212	71	2	C→U	CCU→CUU	P→L
nad4	328	110	1	C→U	CCU→UCU	P→S
nad4	329	110	2	C→U	UCU→UUU	S→F
nad4	349	117	1	C→U	CCC→UCC	P→S
nad4	350	117	2	C→U	UCC→UUC	S→F
nad4	362	121	2	C→U	CCA→CUA	P→L
nad4	436	146	1	C→U	CUU→UUU	L→F
nad4	448	150	1	C→U	CUU→UUU	L→F
nad4	487	163	1	C→U	CUU→UUU	L→F
nad4	497	166	2	C→U	ACA→AUA	T→I
nad4	521	174	2	C→U	UCA→UUA	S→L

nad4	613	205	1	C→U	CAU→UAU	H→Y
nad4	680	227	2	C→U	CCU→CUU	P→L
nad4	716	239	2	C→U	UCA→UUA	S→L
nad4	749	250	2	C→U	UCC→UUC	S→F
nad4	872	291	2	C→U	ACU→AUU	T→I
nad4	923	308	2	C→U	CCG→CUG	P→L
nad4	1045	349	1	C→U	CCU→UCU	P→S
nad4	1055	352	2	C→U	UCC→UUC	S→F
nad4	1061	354	2	C→U	UCU→UUU	S→F
nad4	1067	356	2	C→U	ACU→AUU	T→I
nad4	1085	362	2	C→U	UCA→UUA	S→L
nad4	1118	373	2	C→U	CCC→CUC	P→L
nad4	1160	387	2	C→U	ACA→AUA	T→I
nad4	1229	410	2	C→U	UCU→UUU	S→F
nad4	1268	423	2	C→U	CCA→CUA	P→L
nad4	1346	449	2	C→U	CCG→CUG	P→L
nad4L	35	12	2	C→U	UCU→UUU	S→F
nad4L	89	30	2	C→U	UCA→UUA	S→L
nad4L	104	35	2	C→U	UCA→UUA	S→L
nad4L	152	51	2	C→U	UCG→UUG	S→L
nad4L	182	61	2	C→U	UCA→UUA	S→L
nad4L	191	64	2	C→U	CCA→CUA	P→L
nad5	116	39	2	C→U	UCG→UUG	S→L
nad5	212	71	2	C→U	GCU→GUU	A→V
nad5	239	80	2	C→U	CCG→CUG	P→L
nad5	355	119	1	C→U	CUU→UUU	L→F
nad5	395	132	2	C→U	UCU→UUU	S→F
nad5	502	168	1	C→U	CCU→UCU	P→S
nad5	503	168	2	C→U	UCU→UUU	S→F
nad5	611	204	2	C→U	GCC→GUC	A→V
nad5	614	205	2	C→U	CCC→CUC	P→L
nad5	649	217	1	C→U	CUU→UUU	L→F
nad5	689	230	2	C→U	UCG→UUG	S→L
nad5	695	232	2	C→U	ACU→AUU	T→I

nad5	701	234	2	C→U	UCA→UUA	S→L
nad5	836	279	2	C→U	ACU→AUU	T→I
nad5	875	292	2	C→U	ACU→AUU	T→I
nad5	1366	456	1	C→U	CAU→UAU	H→Y
nad5	1375	459	1	C→U	CCC→UCC	P→S
nad5	1397	466	2	C→U	UCA→UUA	S→L
nad5	1523	508	2	C→U	UCC→UUC	S→F
nad5	1535	512	2	C→U	GCU→GUU	A→V
nad5	1586	529	2	C→U	ACU→AUU	T→I
nad5	1619	540	2	C→U	CCC→CUC	P→L
nad6	70	24	1	C→U	CAU→UAU	H→Y
nad6	86	29	2	C→U	CCC→CUC	P→L
nad6	92	31	2	C→U	CCA→CUA	P→L
nad6	143	48	2	C→U	UCC→UUC	S→F
nad6	158	53	2	C→U	CCA→CUA	P→L
nad7	38	13	2	C→U	UCG→UUG	S→L
nad7	77	26	2	C→U	UCA→UUA	S→L
nad7	83	28	2	C→U	UCA→UUA	S→L
nad7	200	67	2	C→U	UCU→UUU	S→F
nad7	224	75	2	C→U	ACG→AUG	T→M
nad7	244	82	1	C→U	CAU→UAU	H→Y
nad7	251	84	2	C→U	UCA→UUA	S→L
nad7	316	106	1	C→U	CGU→UGU	R→C
nad7	335	112	2	C→U	UCA→UUA	S→L
nad7	344	115	2	C→U	UCA→UUA	S→L
nad7	383	128	2	C→U	UCA→UUA	S→L
nad7	533	178	2	C→U	UCC→UUC	S→F
nad7	578	193	2	C→U	UCA→UUA	S→L
nad7	698	233	2	C→U	UCG→UUG	S→L
nad7	724	242	1	C→U	CAU→UAU	H→Y
nad7	734	245	2	C→U	UCG→UUG	S→L
nad7	739	247	1	C→U	CUU→UUU	L→F
nad7	769	257	1	C→U	CGC→UGC	R→C
nad7	926	309	2	C→U	UCA→UUA	S→L

nad7	973	325	1	C→U	CCU→UCU	P→S
nad7	1057	353	1	C→U	CGU→UGU	R→C
nad7	1079	360	2	C→U	UCU→UUU	S→F
nad7	1088	363	2	C→U	UCA→UUA	S→L
nad7	1103	368	2	C→U	UCU→UUU	S→F
nad7	1124	375	2	C→U	CCA→CUA	P→L
nad9	92	31	2	C→U	UCU→UUU	S→F
nad9	113	38	2	C→U	CCA→CUA	P→L
nad9	167	56	2	C→U	UCG→UUG	S→L
nad9	190	64	1	C→U	CAU→UAU	H→Y
nad9	328	110	1	C→U	CGG→UGG	R→W
nad9	368	123	2	C→U	UCC→UUC	S→F
nad9	398	133	2	C→U	UCA→UUA	S→L
nad9	439	147	1	C→U	CUU→UUU	L→F
rpl2	1271	424	2	C→U	GCG→GUG	A→V
rpl2	404	135	2	C→U	GCG→GUG	A→V
rpl5	7	3	1	C→U	CCA→UCA	P→S
rpl5	35	12	2	C→U	UCA→UUA	S→L
rpl5	59	20	2	C→U	CCG→CUG	P→L
rpl5	92	31	2	C→U	UCG→UUG	S→L
rpl5	169	57	1	C→U	CGC→UGC	R→C
rpl5	329	110	2	C→U	UCG→UUG	S→L
rpl5	512	171	2	C→U	CCA→CUA	P→L
rpl10	239	80	2	C→U	UCG→UUG	S→L
rpl16	37	13	1	C→U	CGU→UGU	R→C
rpl16	104	35	2	C→U	ACU→AUU	T→I
rpl16	176	59	2	C→U	UCC→UUC	S→F
rpl16	208	70	1	C→U	CUC→UUC	L→F
rpl16	377	126	2	C→U	CCA→CUA	P→L
rpl16	383	128	2	C→U	UCG→UUG	S→L
rps1	209	70	2	C→U	UCC→UUC	S→F
rps1	338	113	2	C→U	UCC→UUC	S→F
rps1	524	175	2	C→U	CCU→CUU	P→L
rps1	560	187	2	C→U	ACC→AUC	T→I

rps3	616	206	1	C→U	CUU→UUU	L→F
rps3	674	225	2	C→U	CCC→CUC	P→L
rps3	1481	494	2	C→U	UCA→UUA	S→L
rps4	38	13	2	C→U	UCA→UUA	S→L
rps4	49	17	1	C→U	CGG→UGG	R→W
rps4	464	155	2	C→U	UCU→UUU	S→F
rps4	482	161	2	C→U	UCA→UUA	S→L
rps4	529	177	1	C→U	CGG→UGG	R→W
rps4	721	241	1	C→U	CAU→UAU	H→Y
rps4	775	259	1	C→U	CCU→UCU	P→S
rps4	905	302	2	C→U	UCG→UUG	S→L
rps4	916	306	1	C→U	CAU→UAU	H→Y
rps4	926	309	2	C→U	CCA→CUA	P→L
rps4	941	314	2	C→U	UCU→UUU	S→F
rps7	116	39	2	C→U	CCA→CUA	P→L
rps7	277	93	1	C→U	CUU→UUU	L→F
rps7	332	111	2	C→U	UCA→UUA	S→L
rps7	335	112	2	C→U	UCU→UUU	S→F
rps10	235	79	1	C→U	CGG→UGG	R→W
rps10	313	105	1	C→U	CUC→UUC	L→F
rps12	284	95	2	C→U	UCC→UUC	S→F
rps14	188	63	2	C→U	UCC→UUC	S→F
rps14	199	67	1	C→U	CCU→UCU	P→S
rps19	163	55	1	C→U	CCU→UCU	P→S
rps19	164	55	2	C→U	UCU→UUU	S→F
rps19	242	81	2	C→U	UCG→UUG	S→L
sdh4	916	306	1	C→U	CGG→UGG	R→W
